# Synthesis of Morpholinoamido- and Ester-Disubstituted ε-Caprolactones and Their Ring-Opening (Co)Polymerization

**DOI:** 10.3390/ma18174067

**Published:** 2025-08-30

**Authors:** Maria Orehova, Ema Žagar, David Pahovnik

**Affiliations:** 1 National Institute of Chemistry, Department of Polymer Chemistry and Technology, Hajdrihova 19, 1000 Ljubljana, Slovenia; 2 National Institute of Chemistry, Slovenian NMR Centre, Hajdrihova 19, 1000 Ljubljana, Slovenia; 3 EN-FIST Center of Excellence, Trg Osvobodilne Fronte 13, 1000 Ljubljana, Slovenia

**Keywords:** ε-caprolactone, morpholinoamide substituent, functionalized polyesters, biodegradable polymers, ring-opening polymerization

## Abstract

The development of hydrophilic biodegradable polymers is crucial for a range of biomedical applications, including targeted drug delivery and prosthetics. Ring-opening polymerization of substituted ε-caprolactone monomers provides an efficient method for the synthesis of polyesters with tailored properties. In this work, a synthetic approach for the preparation of ester- and morpholinoamido-disubstituted ε-caprolactone monomers was developed. Poorly defined polymers were obtained from the monomers, bearing two ester groups due to the competitive transesterification of the pendant substituents. On the other hand, the disubstituted morpholinoamido-ε-caprolactone was polymerized in a controlled manner by ring-opening polymerization, and amorphous homopolymers with a high glass transition temperature (112 °C) and good solubility in water were obtained. Statistical and block copolymers with the unsubstituted ε-caprolactone were also prepared, and DLS analysis of the amphiphilic block copolymers in water shows the presence of self-assembled particles. These results demonstrate the potential of morpholinoamido-functionalized ε-caprolactone derivatives as building blocks for the development of biodegradable polymeric materials for biomedical applications.

## 1. Introduction

Synthetic hydrophilic (co)polymers are widely used materials in various fields of biomedicine, including controlled drug/gene delivery systems, orthopedics and tissue engineering [1]. Polyacrylamide [2] and polyethylene glycol (PEG) [3] are commonly used, but show limitations when the inherent degradability of polymeric support is required [4]. Therefore, a lot of efforts have been directed towards the development of biodegradable polymers, in particular appropriately functionalized aliphatic polyesters [5]. The commonly used aliphatic polyesters [6], such as poly(lactic acid), polyglycolide and poly(ε-caprolactone) (PCL), are hydrophobic without pendant functional groups, which limits the possibility of adapting their properties for advanced applications. This challenge has been addressed by the synthesis and (co)polymerization of functionalized ester monomers [7,8,9,10,11,12,13,14,15] that either contain the desired functionality or enable effective post-polymerization modifications.

Various functionalized ε-caprolactones (CLs) have been synthesized via the Baeyer–Villiger oxidation of substituted cyclohexanones [13,16,17,18] and subsequently (co)polymerized by ring-opening polymerization (ROP) with precise control over the polyester molar mass characteristics and architecture [7]. Many of the introduced CL monomer substituents are inherently hydrophilic, which is crucial for the potential biomedical application of their (co)polymers. For example, 5-ethylene ketal CL could be copolymerized with an unsubstituted CL, resulting in PCL with pendant hydroxyl groups after removing its ketal groups, which is dispersible in water [17]. γ-Oligo(ethylene glycol)-substituted CL forms a thermoresponsive water-soluble polymer, while the amphiphilic block copolymer prepared from the same substituted monomer and γ-octyloxy-ε-caprolactone self-assembles in water into micellar structures [19]. In addition to PCLs bearing nonionic hydrophilic functionalities in the structure, PCL polyesters containing ionic groups have also been designed. For instance, CL substituted with a protected amino group was copolymerized with polyethylene glycol as a macroinitiator to prepare amphiphilic block copolymers [20]. After deprotection of the amino groups, the water-soluble polycationic copolymers were produced for use as potential carriers for gene and drug delivery. An alternative approach for the synthesis of cationic PCL is the polymerization of halogen-substituted monomers and their further quaternization with pyridine [21]. To prepare PCL bearing pendant carboxyl groups, the corresponding monomer must contain properly protected carboxyl functional groups. Controlled polymerization of some CL monomers bearing pendant ester functionality was feasible without competitive transesterification of the pendant substituents [22,23,24,25,26]. However, in the case of γ-carboxy-substituted CL monomers, undesired transesterification occurred, resulting in a lower-than-predicted molar mass of the final product [27]. In addition, it is important that the removal of the protecting group after polymerization does not affect the polyester backbone [22].

Amide substituents on the polyester chain have also attracted considerable attention as they allow precise tuning of stimuli-responsive behavior and thus significantly increase the potential of these materials as drug carriers [28,29,30,31]. While a morpholinoamide pendant group has not yet been introduced into the polyester chain, it is being actively utilized for the development of polymers for biomedical applications. In particular, poly(*N*-acryloylmorpholine) (PNAM) is often proposed as an alternative to PEG [32]. PNAM shows no non-specific protein binding, making it a non-cytotoxic and weakly immunogenic polymer [33]. The morpholino functionality greatly increases the hydrophilicity of the macromolecule and provides a non-bonding electron pair at the oxygen atom, which enables the formation of a shell of coordinated water molecules [34]. PNAM serves as a hydrophilic block in amphiphilic polymers that form micelles and have been proposed for drug delivery [35,36,37]. For example, H_2_O_2_-redox responsive micelles for encapsulation of the hydrophobic anticancer drug paclitaxel were prepared from the block copolymer poly(*N*-acryloylmorpholine)-*b*-poly(2-acryloyloxyethyl ferrocene carboxylate) [36]. At the same time, PNAM-based hydrogels can be used for the controlled release of water-soluble drugs such as the antibiotic cyprofloxacin [38]. Although PNAM has shown advantages over PEG in some cases [37], both polymers have the disadvantage of being non-biodegradable. Consequently, there are concerns about accumulation in the body upon frequent use [3]. Therefore, there is a need for the development of a biodegradable alternative to PNAM.

In this work, we report the synthesis of disubstituted CL monomers bearing either ester or morpholinoamido pendant groups. While we assume that the ester pendant groups provide poor control over the polymerization process as they can be involved in extensive side reactions, we hypothesize that the morpholinoamido-substituted CL monomer on the other hand can be (co)polymerized in a well-controlled manner to produce water-soluble functionalized PCL. Since the incorporation of two substituents per monomer unit significantly increases the density of the functional groups in the resulting (co)polymers and thus provides good control over the tuning of hydrophilicity, we intend to copolymerize the disubstituted CL monomer with unmodified CL to produce both random and block copolymers with tunable properties. This would open a way to new materials with finely tuned properties potentially suitable for biomedical applications. The random copolymers could serve as scaffolds in prosthetics and tissue engineering, providing a matrix that supports cell adhesion, proliferation and interaction with the surrounding tissue, while gradually degrading in a controlled manner. The block copolymers could self-assemble into micellar structures, which would enable encapsulation and targeted delivery of hydrophobic drugs, and could in turn improve therapeutic efficacy.

## 2. Materials and Methods

### 2.1. Materials

Fumaric acid (≥99.0%), benzyl bromide (98%), triethylamine (≥99.0%), diethyl fumarate (97%), fumaryl chloride (95%), morpholine (≥99.0%), hydroquinone (≥99%), potassium carbonate (K_2_CO_3_, ≥99.0%), *m*-chloroperbenzoic acid (*m*CPBA, ≤77%), sodium thiosulfate (Na_2_S_2_O_3_, 99%), sodium hydrocarbonate (NaHCO_3_, ≥99.7%), sodium sulfate (Na_2_SO_4_, anhydrous, ≥99.0%), urea hydrogen peroxide (97%), trifluoroacetic anhydride (≥99.0%), acetic acid (≥99%), trimethylaluminium solution (2.0 M in toluene), benzyl alcohol (99.8%, anhydrous), potassium methoxide (KOMe, 95%) and 3-phenyl-1-propanol (98%) were purchased from Sigma Aldrich, St. Louis, MO, USA. Di-sodium hydrogen phosphate (Na_2_HPO_3_, ≥99.0%) and the solvents methanol (≥99.9%), acetonitrile (≥99.5%), ethyl acetate (≥99.5%), toluene (≥99.9%), ethanol (≥99.9%, anhydrous) were purchased from Supelco (Sigma-Aldrich), St. Louis, MO, USA. Acetonitrile (99.8%, anhydrous) and dichloromethane (≥99.8%, anhydrous) were purchased from Sigma Aldrich, St. Louis, MO, USA. Dichloromethane (DCM, 99.9%) and hexane (≥99.0%) were purchased from Honeywell Charlotte, NC, USA. Diethyl ether (99.7%) and *N*,*N*-dimethylformamide (DMF, 99.8%) were purchased from Merck, Darmstadt, Germany. All solvents were used as received. 1,2-Dichloroethane (≥99.5%, Supelco (Sigma-Aldrich), St. Louis, MO, USA) was distilled over phosphorous pentoxide and dried over molecular sieves of a 4 Å pore size. ε-Caprolactone (97%, Sigma-Aldrich, St. Louis, MO, USA) was dried over calcium hydride and distilled under vacuum. Tin(II) 2-ethylhexanoate (92.5–100.0%, Sigma-Aldrich, St. Louis, MO, USA) was distilled under reduced pressure two times. 2-Trimethylsiloxy-1,3-butadiene (**1**) was prepared according to the method reported by Pan et al. [39]. 1-Cyclohexyl-3-phenyl-2-thiourea was prepared according to the method reported by Wang et al. [40].

### 2.2. Characterization Methods

^1^H (299.9 MHz) and ^13^C (75.4 MHz) NMR spectra were recorded on an Agilent Technologies DD2 spectrometer (Agilent Technologies, Santa-Clara, CA, USA) in pulse Fourier transform mode. Chemical shifts are reported in parts-per-million (ppm) relative to tetramethylsilane (TMS) for ^1^H and to a CHCl_3_ or dimethyl sulfoxide (DMSO) solvent signal for ^13^C nuclear magnetic resonance (NMR) spectra. Fourier transform infrared spectra (FT-IR) spectra were recorded in attenuated total reflectance mode using a Perkin Elmer Spectrum One spectrometer (Perkin Elmer, Waltham, MA, USA) in the range of 4000–650 cm^−1^. Size-exclusion chromatography coupled to a multi-angle light-scattering photometer and refractive index detector (SEC/MALS-RI) was performed using a modulated liquid chromatograph consisting of an Agilent Technologies 1260 series pump and a degasser (Agilent Technologies, Santa-Clara, CA, USA), coupled to a Dawn Heleos-II multi-angle light-scattering photometer with a linearly polarized GaAs laser (λ = 661 nm) and an Optilab rEX interferometric refractometer (RI) operating at the same wavelength as the photometer (both instruments were from Wyatt Technology Co., Santa-Barbara, CA, USA). A PolarGel-L column with a precolumn (Agilent Technologies, Santa-Clara, CA, USA) was used to separate the samples according to their hydrodynamic volume. A solution of lithium bromide in *N*,*N*-dimethylacetamide (0.1 M) at a flow rate of 1 mL/min was used as a solvent and eluent. For the water-soluble morpholinoamido-substituted ε-caprolactone (MAPCL), the same type of SEC column was used with a 0.1 M sodium nitrate aqueous solution at a flow rate of 1 mL/min. The mass of the samples injected onto the column was typically 10.0 × 10^−3^ g, while the solution concentration was typically 10.0 × 10^−2^ g mL^−1^. The *d*n/*d*c values required to calculate the molar mass characteristics of the samples were determined assuming 100% recovery of the sample mass from the column. Astra 5.3.4 software (Wyatt Technology Co., Santa-Barbara, CA, USA) was used for data acquisition and evaluation. Matrix-assisted laser desorption/ionization time-of-flight mass spectrometry (MALDI-TOF MS) measurements were performed using a Bruker UltrafleXtreme MALDI-TOF mass spectrometer (Bruker Daltonik, Bremen, Germany). Samples were dissolved in acetonitrile (10 mg/mL) and mixed with a saturated solution of dithranol in acetonitrile as the matrix and sodium trifluoroacetate in acetonitrile (10 mg/mL) as the cationizing agent in a volume ratio of 1:10:2. A total of 1 µL of the sample solution prepared in this way was spotted onto a target plate (dried-droplet method). The mass spectra of the samples were recorded in reflective positive ion mode. Calibration was performed externally using a mixture of PMMA standards (MALDI validation set PMMA, Fluka Analytical, Seelze, Germany) covering the measured molecular weight range and using the nearest neighbor position method. Differential scanning calorimetry (DSC) measurements were performed using a Mettler Toledo DSC1 system (Mettler Toledo, OH, USA) in a nitrogen atmosphere (20 mL/min). The homopolymers were heated from −80 to 240 °C at a heating rate of 10 K/min and held at 240 °C for 1 min. They were then cooled to −80 °C at a cooling rate of 200 K/min, held at this temperature for 10 min, and finally reheated to 240 °C at a heating rate of 20 K/min. The copolymers were heated from −80 to 200 °C at a heating rate of 10 K/min and held at 200 °C for 1 min. They were then cooled to −80 °C at a cooling rate of 200 K/min, held at this temperature for 10 min and finally reheated again to 200 °C at a heating rate of 20 K/min. The size of the particles formed by the block copolymer in deionized H_2_O was measured at an angle of 173° via dynamic light scattering (DLS) using a Malvern Zetasizer Nano-ZS, Malvern, United Kingdom. The viscosity of the distilled water of 0.8863 mPa and the refractive index of 1.330 at 633 nm at 25 °C were used for data analysis.

### 2.3. Synthesis of Monomers

Dibenzyl fumarate (**2b**). Fumaric acid (5.0 g, 43.1 mmol) was dissolved in DMF (100 mL). Then, benzyl bromide (14.1 g, 9.8 mL, 82.5 mmol) and triethylamine (8.3 g, 11.5 mL, 82.5 mmol) were added. The reaction mixture was stirred at 100 °C for 16 h, then cooled down and precipitated in cold deionized water (800 mL). The product was filtered, washed with cold water and dried under reduced pressure. Afterward, the product was recrystallized from an ethanol/hexane mixture to obtain **2b** (6.4 g, 50%). ^1^H NMR (299.9 MHz, CDCl_3_), *δ* (mult, *J*, *n*H): 7.44–7.33 (m, 10H, Ar), 6.94 (s, 2H, C*H*C*H*) and 5.24 (s, 4H, C*H*_2_Ph).

(E)-1,4-dimorpholinobut-2-ene-1,4-dione (**2c**). A solution of morpholine (34.1 g, 33.8 mL, 392 mmol) and triethylamine (26.3 g, 36.4 mL, 260 mmol) in anhydrous acetonitrile (90 mL) was cooled to 0 °C and a solution of fumaryl chloride (20.0 g, 14.1 mL, 131 mmol) in anhydrous acetonitrile (45 mL) was added dropwise during a period of 1 h. The cooling was stopped and the reaction mixture was stirred for additional 1 h at RT. Afterwards, the solvent was removed under reduced pressure. The residue was recrystallized from ethanol two times, and the product was obtained as white crystals (27.9 g, 84%). ^1^H NMR (299.9 MHz, CDCl_3_), *δ* (mult, *J*, *n*H): 7.39 (s, 2H, C*H*C*H*) and 3.75–3.58 (m, 16H, morpholine fragment-mph). ^13^C NMR (75.4 MHz, CDCl_3_), *δ*: 163.83 (*C*O), 130.98 (*C*H*C*H), 66.73 (O*C*H_2_CH_2_N), 46.30 (OCH_2_*C*H_2_N) and 42.55 (OCH_2_*C*H_2_N).

Diethyl 4-oxocyclohexane-1,2-dicarboxylate (**3a**). A mixture of **1** (7.9 g, 9.6 mL, 55.6 mmol) and **2a** (4.8 g, 4.6 mL, 27.9 mmol) was heated to 130 °C for 24 h and then cooled down to RT. K_2_CO_3_ (40 mg) was then added to the reaction mixture and stirred at RT for 9 h, filtered and evaporated under reduced pressure. The resulting product was purified via column chromatography using an ethyl acetate/hexane mixture from a 10 to 20% gradient to obtain **3a** as a light-yellow liquid (5.0 g, 74%). ^1^H NMR (299.9 MHz, CDCl_3_), *δ* (mult, *J*, *n*H): 4.23–4.10 (m, 4H, CH_3_C*H*_2_O), 3.20–2.98 (m, 2H, C*H*), 2.72–2.62 (m, 1H, COC*H*_2_CH(A)), 2.53–2.24 (m, 4H, COC*H*_2_CH(B), COC*H*_2_CH*_2_*, COCH_2_C*H*_2_(A)), 2.00–1.83 (m, 1H, COCH_2_C*H*_2_(B)) and 1.30–1.21 (m, 6H, C*H*_3_CH_2_O). IR (ATR, cm^−1^): 2981, 1720, 1372, 1276 and 1175 cm^−1^.

Dibenzyl 4-oxocyclohexane-1,2-dicarboxylate (**3b**). A mixture of **1** (0.96 g, 1.17 mL, 6.76 mmol) and **2b** (1.00 g, 3.38 mmol) was heated to 140 °C for 24 h and then cooled down to RT. Then, K_2_CO_3_ (10 mg) was added and the mixture was stirred at RT for 5 h, filtered and evaporated under reduced pressure. The resulting product was purified via column chromatography using an ether/hexane mixture from a 10 to 40% gradient to obtain **3b** as white crystals (0.93 g, 75%). ^1^H NMR (299.9 MHz, CDCl_3_), *δ* (mult, *J*, *n*H): 7.40–7.26 (m, 10H, Ar), 5.16–5.01 (m, 4H, PhC*H*_2_), 3.30–3.07 (m, 2H, C*H*C*H*), 2.74–2.64 (m, 1H, COCH_2_CH(A)), 2.52–2.25 (m, 4H, COC*H*_2_CH(B), COCH_2_CH_2_, COCH_2_C*H*_2_(A)) and 2.00–1.84 (m, 1H, COCH_2_C*H*_2_(B)). ^13^C NMR (75.4 MHz, CDCl_3_), *δ*: 206.70 (*C*O), 172.76 (*C*(O)O), 172.31 (*C*(O)O), 135.40, 135.29, 128.56, 128.54, 128.37, 128.36, 128.18, 128.15 (Ar), 66.94 (Ph*C*H_2_), 66.86 (Ph*C*H_2_), 44.38 (CO*C*H_2_CH), 42.99 (*C*H), 41.54 (CO*C*H_2_CH_2_), 39.26 (*C*H) and 27.10 (COCH_2_*C*H_2_). IR (ATR, cm^−1^): 2947, 1732, 1387, 1271, 1176 and 1148.

Di-3,4-morpholinoamido cyclohexanone (**3c**). A solution of **1** (1.26 g, 1.54 mL, 8.87 mmol) and **2c** (1.00 g, 3.94 mmol) in acetonitrile (3.0 mL) was heated at 160 °C for 46 h in a closed vessel. The reaction mixture was cooled down and diluted with methanol (5 mL) and 50 mg of K_2_CO_3_ was added. After 2 h, the solution was filtered and the solvent was evaporated under reduced pressure. The residue was purified via column chromatography (ethyl acetate/methanol 8:1). The obtained product was colorless oil that slowly crystallizes upon storage (0.92 g, 72%). ^1^H NMR (299.9 MHz, CDCl_3_), *δ* (mult, *J*, *n*H): 3.80–3.40 (m, 18H, mph, C*H*C*H*), 2.65–2.35 (m, 4H, C*H*_2_C(O)C*H*_2_), 2.15–2.05 (m, 1H, C(O)CH_2_C*H*_2_(A)) and 2.00–1.80 (m, 1H, C(O)CH_2_C*H*_2_(B)). ^13^C NMR (75.4 MHz, CDCl_3_), *δ*: 207.80 (*C*O), 172.05 (*C*(O)N), 171.41 (*C*(O)N), 67.03 (mph), 66.99 (mph), 46.55 (mph), 46.50 (mph), 43.26 (C(O)*C*H_2_CH), 42.61 (mph), 42.53 (mph), 41.99 (*C*H), 41.03 (C(O)*C*H_2_CH_2_), 40.07 (*C*H) and 28.27 (C(O)CH_2_*C*H_2_). IR (ATR, cm^−1^): 2961, 2921, 2854, 1714, 1627, 1436, 1360, 1225 and 1110.

Diethyl 7-oxooxepane-3,4-dicarboxylate (**4a**) and diethyl 2-oxooxepane-4,5-dicarboxylate (**5a**). Procedure 1: To a solution of **3a** (726 mg, 3.0 mmol) in dichloromethane (25 mL) cooled to 0 °C, *m*CPBA (77%, 1.35 g, 6.0 mmol) was added. Cooling was stopped and the reaction mixture was then stirred at RT for 48 h, diluted with dichloromethane (10 mL) and washed with a Na_2_S_2_O_3_ solution (10%, 50 mL), saturated aqueous NaHCO_3_ (3 × 50 mL) and water (50 mL), dried over Na_2_SO_4_, and finally evaporated under reduced pressure. The resulting mixture of products was separated and purified via column chromatography using an ethyl acetate/hexane mixture from a 20 to 65% gradient to obtain **4a** (403 mg, 52%) as a light-yellow oil and **5a** (116 mg, 15%) as white needles. Procedure 2: Trifluoroacetic anhydride (1.16 g, 0.78 mL, 5.52 mmol) was added dropwise to a mixture of **3a** (0.20 g, 0.83 mmol), urea hydrogen peroxide (0.47 g, 5.00 mmol) and Na_2_HPO_4_ (0.82 g, 5.77 mmol) in CH_2_Cl_2_ (8 mL) at 0 °C. Cooling was stopped and the reaction mixture was stirred for 18 h at RT. Then, the mixture was diluted with dichloromethane and washed with Na_2_S_2_O_3_ solution, saturated aqueous NaHCO_3_ and water, and dried over Na_2_SO_4_. The extracts were filtered, and the solvent was removed under reduced pressure. The resulting mixture of products was separated and purified via column chromatography using an ethyl acetate/hexane mixture from a 20 to 65% gradient to obtain the mixture of isomers **4a** and **5a** (145 mg, 68%). (**4a**) ^1^H NMR (299.9 MHz, CDCl_3_), *δ* (mult, *J*, *n*H): 4.39–4.31 (m, 1H, C(O)OC*H*_2_CH_2_(A)), 4.26–4.13 (m, 5H, C(O)OC*H*_2_CH_2_(B), CH_3_C*H*_2_O), 3.16–3.02 (m, 2H, C*H*C*H*), 3.00–2.80 (m, 2H, C(O)C*H*_2_CH), 2.41–2.29 (m, 1H, C(O)OCH_2_C*H*_2_(A)), 2.11–1.99 (m, 1H, C(O)OCH_2_C*H*_2_(B)) and 1.30–1.24 (m, 6H, C*H*_3_CH_2_O). ^13^C NMR (75.4 MHz, CDCl_3_), *δ*: 172.49, 172.21, 172.13 (*C*O), 66.37 (C(O)O*C*H_2_CH_2_), 61.37 (CH_3_*C*H_2_O), 61.07 (CH_3_*C*H_2_O), 46.02 (*C*H), 40.77 (*C*H), 34.63 (C(O)*C*H_2_CH), 30.39 (C(O)OCH_2_*C*H_2_), 13.88 (*C*H_3_CH_2_O) and 13.84 (*C*H_3_CH_2_O). IR (ATR, cm^−1^): 2983, 1724, 1303, 1254, 1176 and 1027. (**5a**) ^1^H NMR (299.9 MHz, CDCl_3_), *δ* (mult, *J*, nH): 4.51–4.38 (m, 2H, C(O)OC*H*_2_CH), 4.22–4.13 (m, 4H, CH_3_C*H*_2_O), 3.26–3.21 (m, 1H, C*H*), 3.15–3.09 (m, 1H, C*H*), 2.80–2.62 (m, 2H, C(O)C*H*_2_CH_2_), 2.33–2.23 (m, 1H, C(O)CH_2_C*H*_2_(A)), 2.07–1.95 (m, 1H, C(O)CH_2_C*H*_2_(B)) and 1.29–1.23 (m, 6H, C*H*_3_CH_2_O). ^13^C NMR (75.4 MHz, CDCl_3_), *δ*: 174.02, 172.93, 170.35 (*C*O), 66.73 (C(O)O*C*H_2_CH), 61.60 (CH_3_*C*H_2_O), 61.28 (CH_3_*C*H_2_O), 46.27 (*C*H), 44.87 (*C*H), 31.54 (C(O)*C*H_2_CH_2_), 23.43 (C(O)CH_2_*C*H_2_), 14.08 (*C*H_3_CH_2_O) and 14.03 (*C*H_3_CH_2_O). IR (ATR, cm^−1^): 2991, 1720, 1307, 1171 and 1030.

Dibenzyl 7-oxooxepane-3,4-dicarboxylate (**4b**) and dibenzyl 2-oxooxepane-4,5-dicarboxylate (**5b**). Procedure 1: To a solution of **3b** (1.10 g, 3.0 mmol) in dichloromethane (25 mL) cooled to 0 °C, *m*CPBA (77%, 1.35 g, 6.0 mmol) was added. Cooling was stopped, and the reaction mixture was stirred at RT for 48 h, then diluted with dichloromethane (10 mL) and washed with Na_2_S_2_O_3_ (10%, 50 mL), saturated aqueous NaHCO_3_ (3 × 50 mL) and water (50 mL), then dried over Na_2_SO_4_, and finally evaporated under reduced pressure. The resulting mixture of products was purified via column chromatography using an ethyl acetate/toluene mixture from a 5 to 10% gradient to recover **3b** (340 mg, 31%), and to obtain **4b** (429 mg, 37%) as a light-yellow oil that slowly crystalized, as well as **5b** (84 mg, 7%) as white needles. Procedure 2: Trifluoroacetic anhydride (1.92 g, 1.29 mL, 9.12 mmol) was added dropwise to a mixture of **3b** (0.50 g, 1.37 mmol), urea hydrogen peroxide (0.77 g, 8.22 mmol) and Na_2_HPO_4_ (1.37 g, 9.62 mmol) in dichloromethane (13 mL) at 0 °C. Cooling was stopped and the reaction was stirred for 18 h at RT. Then, the reaction mixture was diluted with dichloromethane and washed with Na_2_S_2_O_3_ solution, saturated aqueous NaHCO_3_ and water, and dried over Na_2_SO_4_. The extracts were filtered, and the solvent was removed under reduced pressure. The resulting mixture of products was separated and purified via column chromatography using a toluene/ethyl acetate mixture (12/1) to obtain a mixture of isomers **4b** and **5b** (402 mg, 77%). (**4b**) ^1^H NMR (299.9 MHz, CDCl_3_), *δ* (mult, *J*, *n*H): 7.40–7.24 (m, 10H, Ar), 5.14–5.01 (m, 4H, PhC*H*_2_), 4.35–4.26 (m, 1H, C(O)OC*H*_2_CH_2_), 4.23–4.13 (m, 1H, C(O)OC*H*_2_CH_2_), 3.22–3.08 (m, 2H, C*H*C*H*) and 2.97–2.79 (m, 2H, C(O)C*H*_2_CH), 2.40–2.28 (m, 1H, C(O)OCH_2_C*H*_2_(A)), 2.09–1.97 (m, 1H, C(O)OCH_2_C*H*_2_(B)). ^13^C NMR (75.4 MHz, CDCl_3_), *δ*: 172.29, 172.05, 171.96 (*C*O), 135.15, 135.03, 128.59, 128.55, 128.48, 128.45, 128.36, 128.24 (Ar), 67.33, 67.03, 66.40 (Ph*C*H_2_, C(O)O*C*H_2_CH_2_), 46.21 (*C*H), 40.93 (*C*H), 34.69 (C(O)*C*H_2_CH) and 30.48 (C(O)OCH_2_*C*H_2_). IR (ATR, cm^−1^): 2954, 1726, 1455, 1303, 1253 and 1163. (**5b**) ^1^H NMR (299.9 MHz, CDCl_3_), *δ* (mult, *J*, nH): 7.45–7.24 (m, 10H, Ar), 5.22–5.04 (m, 4H, PhC*H*_2_), 4.52–4.37 (m, 2H, C(O)OC*H*_2_CH), 3.36–3.28 (m, 1H, C*H*), 3.26–3.18 (m, 1H, C*H*), 2.76–2.59 (m, 2H, C(O)C*H*_2_CH_2_), 2.35–2.24 (m, 1H, C(O)CH_2_C*H*_2_ (A)) and 2.09–1.97 (m, 1H, C(O)CH_2_C*H*_2_ (B)). ^13^C NMR (75.4 MHz, CDCl_3_), *δ*: 173.84, 172.65, 170.12 (*C*O), 135.22, 135.07, 128.67, 128.64, 128.57, 128.52, 128.30, 128.29 (Ar), 67.40 (PhCH_2_), 67.10 (PhCH_2_), 66.57 (C(O)O*C*H_2_CH), 46.30 (*C*H), 44.86 (*C*H), 31.47 (C(O)*C*H_2_CH_2_) and 23.40 (C(O)CH_2_*C*H_2_). IR (ATR, cm^−1^): 1714, 1457, 1300, 1268, 1156 and 1139.

Di-3,4-morpholinoamido ε-caprolactone (**4c**, **5c**). A solution of **2** (3.67 g, 11.3 mmol) and *m*CPBA (77%, 6.34 g, 28.3 mmol) in dichloromethane was stirred for 48 h at RT, then the solvent was removed under reduced pressure. The residue was extracted with diethyl ether (50 mL), then purified with column chromatography (ethyl acetate/ethanol 20:1 to 8:1). A total of 3.24 g (84%) of a **4c** and **5c** mixture was obtained. Further recrystallization of the product from ethanol and a diethyl ether mixture afforded the **4c** (2.40 g, 62%) with traces (1–3%) of **5c**. The **4c** was dissolved in a minimal amount of dry acetonitrile at 65 °C. Then, the solvent was evaporated under reduced pressure in order to remove residual ethanol. The procedure was repeated three times. (**4c**) ^1^H NMR (299.9 MHz, CDCl_3_), *δ* (mult, *J*, *n*H): 4.45–4.22 (m, 2H, C(O)OC*H*_2_CH_2_), 3.80–3.32 (m, 18H, mph, C*H*C*H*), 3.03–2.87 (m, 1H, C(O)C*H*_2_CH_2_(A)), 2.58 (d, 1H, *J* 14.1 Hz, C(O)C*H*_2_CH_2_(B)) and 2.10–1.97 (m, 2H, C(O)OCH_2_C*H*_2_). ^13^C NMR (75.4 MHz, CDCl_3_), *δ*: 173.01 (*C*(O)O), 171.87 (*C*(O)N), 171.54 (*C*(O)N), 67.22 (C(O)O*C*H_2_CH_2_), 66.98, 66.88, 66.78 (mph), 46.57 (mph), 44.40 (*C*H), 42.62 (mph), 42.50 (mph), 37.90 (*C*H), 36.17 (C(O)*C*H_2_CH) and 31.82 (C(O)OCH_2_*C*H_2_). IR (ATR, cm^−1^): 2960, 2922, 2852, 1721, 1637, 1622, 1447, 1299, 1255, 1215 and 1110. (**5c**) ^1^H NMR (299.9 MHz, CDCl_3_), *δ* (mult, *J*, nH): 4.40–4.30 (dd, *J* 9.0 Hz, *J* 13.5 Hz, 1H, C(O)O*C*H_2_CH(A)), 4.19–4.13 (dd, *J* 1.4 Hz, *J* 13.5 Hz, 1H, C(O)O*C*H_2_CH(B)), 3.80–3.35 (m, 18H, mph, C*H*C*H*), 2.83–2.69 (m, 2H, C(O)C*H*_2_CH_2_) and 2.10–1.80 (m. 2H, C(O)CH_2_C*H*_2_). ^13^C NMR (75.4 MHz, CDCl_3_), *δ*: 173.85 (*C*(O)O), 171.82 (*C*(O)N), 169.71 (*C*(O)N), 68.37 (C(O)O*C*H_2_CH), 67.02, 66.94, 66.92 (mph), 46.76 (mph), 46.61 (mph), 44.78 (*C*H), 44.70 (*C*H), 42.50 (mph), 32.60 (C(O)*C*H_2_CH_2_) and 25.50 (C(O)CH_2_*C*H_2_). IR (ATR, cm^−1^): 2961, 2922, 2853, 1734, 1631, 1436, 1262, 1223 and 1110.

### 2.4. General Procedure of ***4c*** Polymerization

Procedure 1. Dry 1,2-dichloroethane was added to monomer **4c** (1 mL per 250 mg), followed by the addition of benzyl alcohol and a solution of trimethylaluminium (2.0 M in toluene). The reaction mixture was heated to 60 °C and then quenched with acetic acid solution in dichloromethane (0.1 M). The reaction mixture was diluted with dichloromethane. Then, the polymer was precipitated in cold diethyl ether. The precipitate was centrifuged and dried under reduced pressure at 100 °C.

Procedure 2. The **4c** mixture was added to the solution of 1-cyclohexyl-3-phenyl-2-thiourea and potassium methoxide (0.25 M/0.05 M) in dichloromethane, and the reaction was kept at RT. The reaction was stopped with an acetic acid solution in dichloromethane (0.1 M). The reaction mixture was diluted with dichloromethane. Then, the polymer was precipitated in cold diethyl ether, centrifuged and dried under reduced pressure at 100 °C.

MAPCL: ^1^H NMR (299.9 MHz, CDCl_3_), *δ* (mult, *J*, *n*H): 7.37–7.23 (m, Ar), 5.15–5.03 (m, PhC*H*_2_), 4.15–3.35 (m, 19H, mph, C(O)OC*H*_2_CH_2_, C*H*), 3.18–2.98 (m, 1H, C*H*), 2.85–2.68 (m, 1H, C(O)C*H*_2_CH(A)), 2.64–2.44 (m, 1H, C(O)C*H*_2_CH(B)) and 2.07–1.74 (m, 2H, C(O)CH_2_C*H*_2_). ^13^C NMR (75.4 MHz, CDCl_3_), *δ*: 172.47, 172.41, 171.77, 171.68 (*C*O), 128.77, 128.45 (Ar), 67.08, 66.85 (mph), 47.17, 46.83, 42.61, 42.48 (mph), 39.82, 39.54 (*C*H), 38.96 (*C*H), 35.17 (C(O)*C*H_2_CH) and 29.61 (OCH_2_*C*H_2_). IR (ATR, cm^−1^): 2962, 2900, 2857, 1730, 1635, 1439, 1355, 1300, 1267, 1227, 1163 and 1112.

### 2.5. Copolymerization Procedure

Statistical copolymer: Procedure 1. Dry 1,2-dichloroethane was added to a mixture of **4c** and CL (200 mg of combined monomers per 1 mL), followed by addition of benzyl alcohol and trimethylaluminium solution (2.0 M in toluene). The reaction mixture was heated to 60 °C and then stopped by addition of acetic acid solution in dichloromethane (0.1 M) and diluted with dichloromethane. The polymer was precipitated in a cold mixture of hexane and diethyl ether. The precipitate was centrifuged and dried under reduced pressure at 100 °C.

Block copolymer: Procedure 2. CL was added to the solution of 1-cyclohexyl-3-phenyl-2-thiourea and potassium methoxide (5/1 molar ratio) in toluene, so the final monomer concentration was 3.0 M. The reaction mixture was kept at RT until CL conversion had reached more than 95% (according to NMR spectrum of reaction mixture aliquot). Afterwards, **4c** was added. The reaction mixture was kept at RT, then stopped with acetic acid solution in dichloromethane (0.1 M). The reaction mixture was diluted with dichloromethane. Then, the polymer was precipitated in cold diethyl ether. The precipitate was centrifuged and dried under reduced pressure at 100 °C.

Procedure 3. The procedure is analogous to procedure 1, except that a PCL macroinitiator (M_n_ 4.3, Ð 1.03), prepared using the method described in the literature [41], was used instead of the alcohol.

MAPCL-PCL: ^1^H NMR (299.9 MHz, CDCl_3_), *δ* (mult, *J*, *n*H): 4.19–3.20 (m, 19H, C(O)OC*H*_2_CH_2_ (PCL), mph, C(O)OC*H*_2_CH_2_ (MAPCL), C*H*), 3.18–2.98 (m, 1H, C*H*), 2.85–2.64 (m, 1H, C(O)C*H*_2_CH(A)), 2.64–2.41 (m, 1H, C(O)C*H*_2_CH(B)), 2.38–2.22 (t, *J* 7.5 Hz, 2H, C(O)C*H*_2_CH_2_ (PCL)), 2.07–1.74 (m, 2H, C(O)CH_2_C*H*_2_ (MAPCL)), 1.71–1.55 (m, 4H, C(O)CH_2_C*H*_2_CH_2_C*H*_2_) and 1.45–1.30 (m, 2H, C(O)CH_2_CH_2_C*H*_2_CH_2_).

### 2.6. Micelle Preparation

A total of 5 mg of the PCL-*b*-MAPCL copolymer was dissolved in 100 μL of acetone. Then, 5 mL of deionized water was added. The solution was stirred under reduced pressure (40 mbar) at 30 °C for 2 h to remove the acetone. Afterwards, the solution was filtered and analyzed via DLS.

## 3. Results and Discussion

**Scheme 1:**
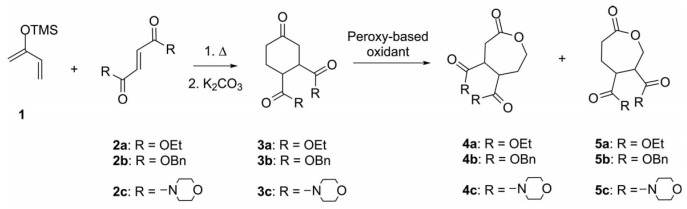
Diels–Alder reaction with deprotection followed by Baeyer–Villiger oxidation to yield a mixture of disubstituted ε-caprolactone regioisomers.

We chose the Diels–Alder reaction to prepare the 3,4-disubstituted cyclohexanones. The diene component **1** was prepared from 3-butenone according to a known procedure [39]. In combination with various dienophiles, it could be used for the preparation of 3,4-disubstituted cyclohexanones. Such a synthetic approach is beneficial for two reasons: (i) the usual oxidation step of terpene-based substrates is omitted, since the ketone group, masked as a protected enol, is introduced directly into the ring, and (ii) there is a wide selection of differently substituted dienophiles suitable for the Diels–Alder reaction, which greatly expands the possible functionalities that can be introduced into the 3,4-disubstituted cyclohexanones. In our case, we used fumaric acid derivatives (Scheme 1), where we investigated three different substituents, i.e., (i) the ethyl ester group as a model for ester-substituted monomers, (ii) the benzyl ester group, which can be removed after polymerization by hydrogenation without affecting the polyester backbone to obtain carboxylated polyesters [25,27], and (iii) the morpholinoamide group for the synthesis of nonionic hydrophilic polyesters. The morpholinoamide group makes the monomer and its precursor sufficiently soluble in polar organic solvents, which simplifies manipulation during monomer synthesis, as the introduction of the unsubstituted fumaroamide proved to be impracticable due to its poor solubility in organic solvents.

The Diels–Alder cycloaddition of **1** and **2a** was performed according to a known method [39] with the neat diethyl fumarate at 130 °C, followed by deprotection of the crude silyl-protected enol (K_2_CO_3_ in EtOH), which gave us the desired **3a** in a 74% yield (Scheme 1). Using the benzyl ester of fumaric acid, the same procedure afforded **3b** in a 75% yield (Scheme 1). The cycloaddition of (E)-1,4-dimorpholinebut-2-ene-1,4-dione **2c** and **1** required a solvent due to the high melting temperature of **2c**. The reaction was performed in acetonitrile at 160 °C in a closed vessel. The deprotection of the ketone group resulted in the ketone **3c** with a 72% yield (Scheme 1). We assume that maleic acid derivatives that allow Diels–Alder cycloaddition under milder conditions could also be used in this scheme. As a result, the polyester chain would have a different stereochemistry, which would probably not affect the hydrophilicity; however, conformational effects could result in shifts in glass transition temperature and crystallinity. Nevertheless, in this study, we focus on fumarate derivatives, as they offer a more sustainable alternative, which could be derived from lignocellulosic biomass.

Transformation of the disubstituted cyclohexanones **3a**, **3b** and **3c** into the disubstituted ε-caprolactones was carried out using the Baeyer–Villiger oxidation reaction with *m*CPBA (Table 1, Procedures 1, 2 and 3) at RT in dichloromethane. This resulted in a complete conversion of **3a** and **3c** into the lactones **4a**, **5a** and **4c**, **5c**, respectively, after 48 h of reaction time. However, under the same reaction conditions, a significant amount of the starting compound **3b** (38%) was still present in the reaction mixture. The reaction products consisted of β,γ-disubstituted (**4**) and γ,δ-disubstituted (**5**) regioisomers, with **4** being at least four times more abundant than **5**, as determined by the ^1^H NMR spectra of the reaction mixture prior to separation via column chromatography (Table 1). The relative amounts of regioisomers **4** and **5** formed are consistent with the electronic effects of the Baeyer–Villiger reaction, in which the preferential migration of the more electron-rich methylene fragment normally occurs in a rearrangement step. All monomers obtained were characterized by ^1^H and ^13^C NMR, where the characteristic signals of the –CH_2_O– fragments formed appeared as multiplets between 4.0 and 4.5 ppm in the ^1^H spectra and between 66 and 69 ppm in the ^13^C spectra (Figures S9–S24).

In order to improve the reaction yield of the Baeyer–Villiger reaction, we studied different reaction conditions and also the type of oxidizing agent. For this purpose, the reaction was carried out with *m*CPBA as an oxidant in CHCl_3_ at elevated temperature (65 °C). Under these reaction conditions, the two ester-substituted derivatives **3a** and **3b** were completely converted; however, in contrast to the reactions performed at RT, a considerable amount of unidentified side products were also formed, which made the separation and purification of the two regioisomers very difficult. Due to the moderate reaction rate of the Baeyer–Villiger oxidation of the disubstituted cyclohexanones, we next tested the method developed for the macrocyclic ketones, which are known to be oxidized very slowly. This method includes trifluoroperacetic acid (TFPAA) [42] as one of the most active reagents for this type of reaction. TFPAA, which is not commercially available, was prepared in situ from the complex of trifluoroperacetic anhydride and urea–hydrogen peroxide with Na_2_HPO_4_ as a buffer [43]. Under these conditions, both **3a** and **3b** were completely converted in only 16 h without formation of any side products (Table 1, Procedures 4 and 5). The regioselectivity of the oxidation did not change significantly compared to the reaction with *m*CPBA. The morpholine-substituted ketone **3c** was not treated with this method due to its solubility in water and the resulting issues with the isolation of **4c** and **5c**.

Although the synthesis of these monomers requires a comparatively large amount of oxidizing agent and can occasionally lead to side reactions, overall, the approach offers efficient and straightforward access to CL monomers in high yields from readily available precursors, including those derived from lignocellulosic biomass processing.

**Figure 1:**
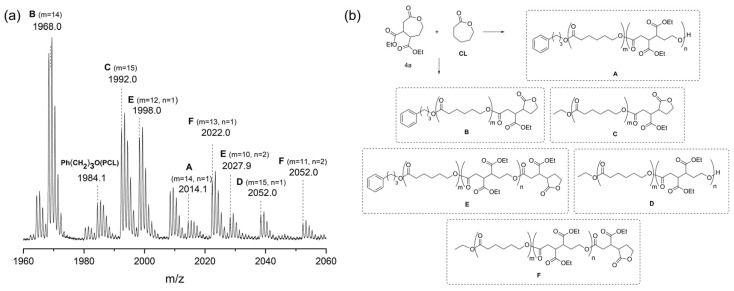
(**a**) MALDI-TOF mass spectrum of the aliquot of the copolymerization mixture **4a**, **5a** and CL catalyzed by Sn(Oct)_2_; (**b**) proposed structures of the polymer chains obtained by ROP of **4a** and CL. For the sake of simplicity, only the main isomer **4a** is shown, since **5a** undergoes similar transformations.

After successful synthesis of the disubstituted CL monomers, we performed their (co)polymerization using ROP. The first attempt was made with a mixture of the diethyl ester monomers **4a** and **5a**, which were copolymerized with CL in toluene. For this purpose, tin(II) 2-ethylhexanoate [44] was used as a catalyst and 3-phenyl-1-propanol as an initiator. The MALDI-TOF mass spectrum of the reaction mixture aliquot shows several peak populations as a consequence of the uncontrolled polymerization process due to the extensive transesterification involving pendant ester substituents (Figure 1). While some chains are initiated by the 3-phenyl-1-propanol (A), some are instead initiated by ethanol (D), and the majority of chains appear to have lost an ethanol (B, C, E, F), most likely due to a nucleophilic attack of the terminal hydroxyl of the disubstituted ε-caprolactone moiety on its own pendant ester group. As a result, a five-membered ring is formed, which is stable under the given reaction conditions and leads to in-chain termination. The released ethanol acts as an initiator for new macromolecular chain. Similar problems have already occurred in the polymerization of γ-hydroxy ε-caprolactones with an ester protecting group [45,46]. In this case, the part of the monomer structure was rearranged into a stable five-membered monomer cycle, resulting in a loss of yield. In our case, cyclization to a five-membered ring caused polymerization termination. It is likely that a similar side reaction was involved in the polymerization of ε-caprolactones with γ-pendant ester groups previously reported by Hedrick’s research group, where only oligomers with significantly lower molar masses than expected were formed [27]. Fukushima’s group also encountered the problem of five-membered ring formation in the polymerization of γ-carbonyl-substituted CL [26].

To try to avoid the side reaction with the pendant group, we tested different catalytic systems, which usually provide much better control over the polymerization process, e.g., diphenyl phosphate [41], 1-cyclohexyl-3-phenyl-2-thiourea/1,3-dimesitylimidazol-2-ylidene [47] complex and methylaluminium bis(2,6-di-phenylphenolate) [48], but no improvement was achieved. The same results were obtained when we copolymerized single regioisomers **4a** or **5a** with CL, as well as when **4a** was homopolymerized. Copolymerization of CL with a mixture of monomers **4b** and **5b** with the benzyl ester pendant groups also resulted in the same side reactions. While the controllable polymerization of the CL monomer with the ester pendant group using diphenyl phosphate as a catalyst was successfully performed by the Fukushima group, the presence of two pendant groups per monomer unit in our case dramatically increased the propensity for a side reaction [26].

Next, we focused on the morpholinoamide-substituted CL monomer. As already mentioned, Baeyer–Villiger oxidation produces the two isomers **4c** and **5c**, which are isolated as a mixture by column chromatography. However, further recrystallization of the mixture of **4c** and **5c** affords the main isomer **4c** in a 62% yield with only 1–3% admixture of **5c**. For further polymerization experiments, we only used **4c**, since the polymerization rate of the two isomers could be different.

**Scheme 2:**
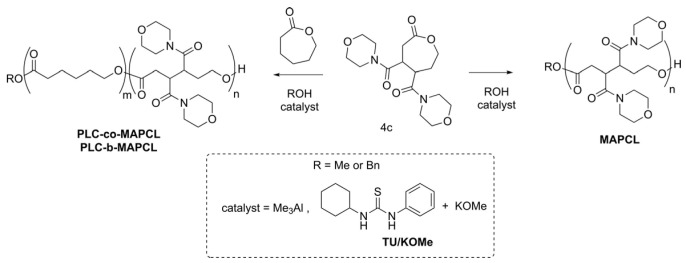
Ring opening (co)polymerization of **4c**.

**Figure 2:**
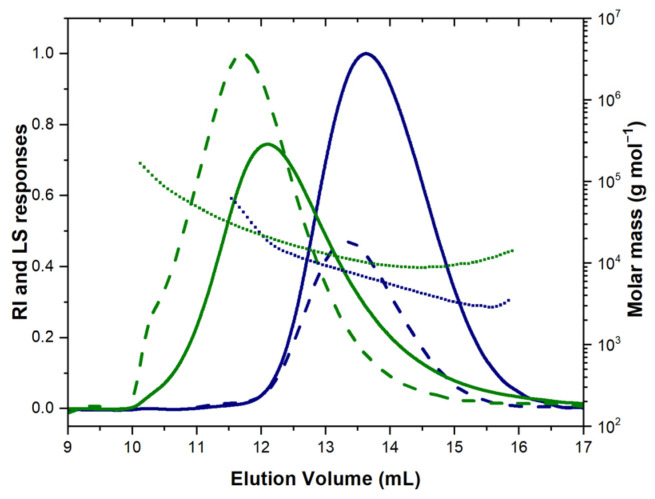
SEC/MALS-RI chromatograms of the homopolymers MAPCL1 (blue lines) and MAPCL2 (green lines). Solid lines: refractive index detector responses; dashed lines: 90° light-scattering detector responses; squares: molar mass vs. elution volume.

While ester-disubstituted monomers proved impractical due to the pronounced side reactions of the pendant ester group, the amide group in the morpholinoamide substituent should be much more resistant to attack by the hydroxyl group under ROP conditions. We expected that monomer **4c** would polymerize slowly due to the presence of bulky morpholinoamide substituents, similarly to what has been observed for bulky carbohydrate-derived permethoxylated CL [49]. The polymerization experiments with diphenylphosphate and tin(II) 2-ethylhexanoate resulted in almost no monomer conversion, so we used the more active alkylaluminium catalyst to polymerize **4c**. The trimethylaluminium-assisted reaction, initiated with benzyl alcohol in dichloromethane at RT, led to a 20% conversion in 4 days. Performing the reaction in 1,2-dichloroethane at 60 °C for 5.5 h, followed by precipitation of the product to remove the oligomers, gave the homopolymer MAPCL1 a weight-average molar mass (*M*_w_) of 7.1 kg mol^−1^ and a dispersity (Đ) of 1.20, as determined by SEC/MALS-RI (Scheme 2, Table 2). Under the same experimental conditions as for MAPCL1 and after 5 days of reaction, a homopolymer with an *M*_w_ of 20 kg mol^−1^ and a dispersity (Đ) of 1.34 was obtained (MAPCL2, Table 2). Both MAPCL homopolymers show a monomodal molar mass distribution (Figure 2) with low dispersity values, while their number-average molar masses (*M*_n_) determined experimentally by SEC/MALS-RI or ^1^H NMR are in good agreement with the target molar masses defined by the monomer/initiator ratio (*M*_theor_; Table 2). The ^13^C NMR spectrum of MAPCL2 (Figure S27), recorded at elevated temperature (50 °C) in order to improve resolution, shows that most of the carbon nuclei show double resonances, indicating slow conformational dynamics most likely caused by bulky substituents and slow rotations around amide bonds. The much longer polymerization time required by MAPCL2 compared to MAPCL1 to achieve high monomer conversion is probably related to the bulky pendant substituents on the growing chain, which hinder the accessibility of the active center for reactions with the monomer by displacing the monomer from its vicinity [50].

As expected, the presence of two morpholinoamido groups per monomer unit significantly increased the hydrophilicity of the polyester compared to the unsubstituted PCL, resulting in good solubility of the MAPCL homopolymers in water (up to several hundred milligrams per mL; ^1^H NMR spectrum of the polymer in D_2_O is shown in Figure S26). The MAPCL homopolymers have an amorphous structure, as indicated by DSC analysis. The DSC thermogram of MAPCL2 shows a very high glass transition temperature of 112 °C (Figure S32), which is a consequence of the limited conformational mobility of the polyester chains due to the presence of bulky morhpolinoamido groups in the structure.

**Figure 3:**
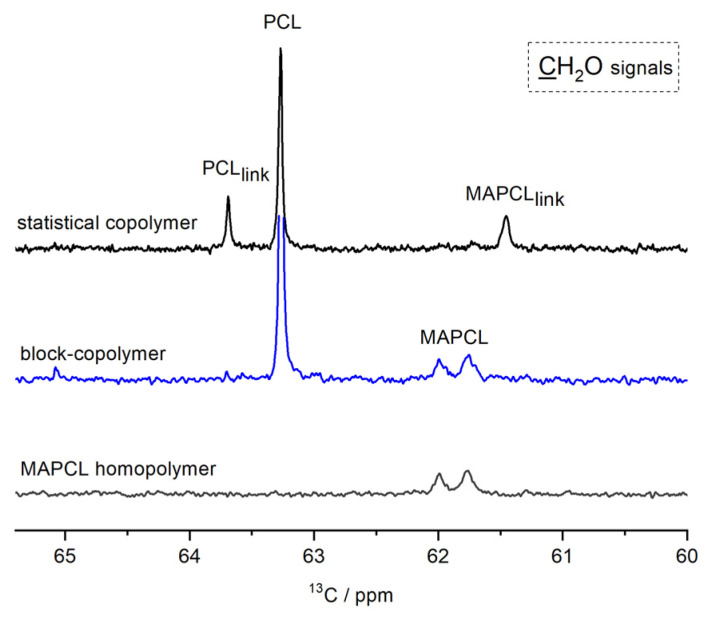
Enlarged ^13^C NMR spectra of the homopolymer (MAPCL3), the statistical copolymer (PCL-*co*-MAPCL) and the block copolymer (PCL-*b*-MAPCL1), showing the signals of the –*C*H_2_O– fragments in the different dyads.

Subsequently, the lactone **4c** was copolymerized with CL using a trimethylaluminium catalyst. The mixture of 75 mol% CL and 25 mol% **4c** was successfully copolymerized to a statistical copolymer PCL-*co*-MAPCL with the same ratio of comonomer units as originally introduced into the reaction mixture. Under the reaction conditions used, a very similar conversion rate of both monomer types was observed according to ^1^H NMR. The ^13^C spectrum of the product (Figure 3 and Figure S35) recorded in DMSO-*d*_6_ at elevated temperature (50 °C) shows three –*C*H_2_O– carbon signals at 63.3, 63.7 and 61.45 ppm, corresponding to the three types of dyads: PCL-to-PCL, PCL-to-MAPCL and MAPCL-to-PCL, respectively. Two signals at 62.0 and 61.8 ppm, corresponding to MAPCL-to-MAPCL dyads, are almost absent, which indicates that the copolymer does not have a blocky structure. The DSC thermogram of PCL-*co*-MAPCL shows no melting transition and a single glass transition at −7.8 °C (Figure S36).

The PCL-*b*-MAPCL1 block copolymer was synthesized from the PCL macroinitiator (*M*_n_ 4.3 kg mol^−1^, Đ 1.03) and **4c** using a trimethylaluminium catalyst. The ^13^C NMR spectrum of PCL-*b*-MAPCL1, in contrast to the spectrum of the statistical copolymer, shows high-intensity signals for –*C*H_2_O– units typical for the PCL and MAPCL blocks, while the signal for PCL-to-MAPCL linking units is of very low intensity (Figure 3 and Figure S41). The DLS histogram of amphiphilic PCL-*b*-MAPCL1 in water shows the presence of self-assembled particles with a bimodal size distribution and average particle diameters of 43 nm and 207 nm (Figure S44). The DSC thermogram of PCL-*b*-MAPCL1 shows two glass transition temperatures at −47.7 °C and 60.5 °C, which most likely correspond to domains reached in the PCL- and MAPCL-block, and almost no melting transition due to the largely hindered stacking of the PCL blocks (Figure S42).

**Figure 4:**
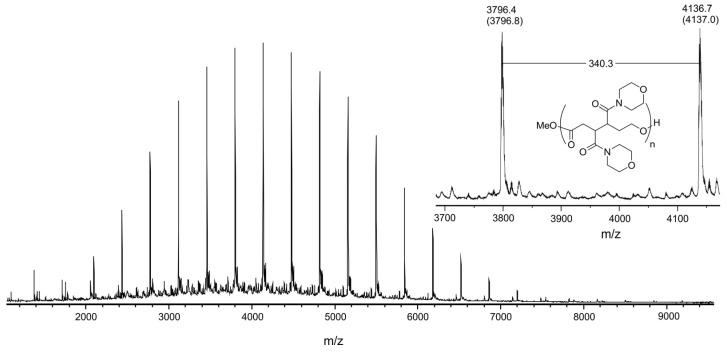
MALDI-TOF mass spectrum of MAPCL3 initiated with methanol. Measured monoisotopic signals are denoted in the enlarged region of the mass spectrum together with the calculated exact masses for [M + Na]^+^ (in parentheses) for the proposed structure.

After the successful (co)polymerizations catalyzed with trimethylaluminium catalyst, we tested another catalytic system, i.e., 3-phenyl-1-cyclohexyl-2-thiourea with sodium methoxide [47]. In this case, **4c** formed the homopolymer MAPCL3 with an *M*_w_ of 5.4 kg mol^−1^ and a Đ of 1.15 (Table 2). The MALDI-TOF mass spectrum of MAPCL3 shows a single set of signals corresponding to methanol-initiated homopolymer chains (Figure 4). However, we were not able to produce high-molar-mass polymers using this method, possibly due to large substituents preventing access of the thiourea-coordinated monomers to the active sites of the growing chains.

The sequential copolymerization of CL and **4c**, catalyzed by 3-phenyl-1-cyclohexyl-2-thiourea with sodium methoxide, was performed in situ. First, CL was polymerized to a conversion of 97%, followed by the addition of the **4c** monomer to the reaction mixture. The ^13^C NMR spectrum shows high-intensity carbon signals for PCL-to-PCL and MAPCL-to-MAPCL –*C*H_2_O– groups at 63.3 pm, 62.0 and 61.8 ppm, respectively, while the signal for PCL-to-MAPCL linking units at 63.7 ppm are of negligible intensity (Figure S46). The SEC/MALS-RI analysis of the obtained copolymer PCL-*b*-MAPCL2 shows a monomodal molar mass distribution with a Đ of 1.07 (Figure S48) while the *M*_w_ of the MAPCL-*b*-PCL2 is 8.2 kg mol^−1^. The MAPCL-*b*-PCL2 is water-soluble and its DLS histogram shows a unimodal size distribution of the self-assembled particles with an average diameter of 34 nm. In the first heating scan, the DSC thermogram of the MAPCL-*b*-PCL2 copolymer shows a crystallization and melting transition of the PCL block at 17.5 °C and 50.5 °C and its glass transition at −61 °C (Figure S47), which is close to the values of the PCL homopolymer. The glass transition of the amorphous MAPCL block is barely seen. The results show that the 3-phenyl-1-cyclohexyl-2-thiourea/potassium methoxide catalytic system affords synthesis of well-defined MAPCL-*b*-PCL block copolymers, but the length of the MAPCL block is limited.

## 4. Conclusions

A synthetic approach towards β,γ- and γ,δ-diesters and morholinoamido-disubstituted ε-caprolactones was developed. The approach includes Diels–Alder cycloaddition and Baeyer–Villiger oxidation of the obtained cyclohexanone. Our efforts to polymerize the monomers bearing two ethyl ester or two benzyl ester substituents failed due to the transesterification reaction involving pendant ester groups. In contrast, the morpholinoamido-disubstituted CL was successfully polymerized in the presence of a trimethylaluminium catalyst or a complex of substituted thiourea and potassium methoxide, resulting in MAPCL homopolymers with controlled molar mass characteristics. MAPCL was found to be an amorphous and water-soluble polymer with a high glass transition temperature of 112 °C. The statistical copolymerization of morpholinoamido-disubstituted CL with unsubstituted CL was also feasible with a trimethylaluminium catalyst and resulted in copolymers with randomly distributed comonomer units. Amphiphilic PCL-*b*-MAPCL block copolymers were also synthesized from the PCL macroinitiator using either a trimethylaluminium catalyst or 3-phenyl-1-cyclohexyl-2-thiourea with sodium methoxide. The DLS histogram of PCL-*b*-MAPCL copolymers shows the presence of self-assembled particles.

Our approach provides a route to the morpholinoamido-disubstituted CL monomer and enables its ring opening polymerization in a controlled manner. The resulting MAPCL could serve as a hydrophilic block of block copolymers. Since such copolymers are able to form micelles in aqueous solution, they could be used for the encapsulation and delivery of hydrophobic drug molecules, such as the anticancer agents paclitaxel or doxorubicin. Morpholinoamido-disubstituted CL repeating units could also be used to tune the properties of polyesters as components of statistical copolymers, which is important for the development of prosthetic materials, where hydrophilicity is essential for cell adhesion. In addition, the incorporation of MAPCL into cross-linked materials could be used to design systems for the controlled release of hydrophilic drugs. Therefore, MAPCL has the potential to be a degradable alternative to non-degradable, water-soluble PEG and PNAM for use in drug delivery.

## Figures and Tables

**Table 1:** Optimal reaction conditions and oxidants used for the Baeyer–Villiger oxidation of **3a**–**3c**.

Entry	R	Oxidant	Time, h	Reaction Mixture Composition, mol % ^1^		
				3	4	5
1	OEt	*m*CPBA	48	-	83	17
2	OBn	*m*CPBA	48	38	53	9
3	morpholine	*m*CPBA	48	-	81	19
4	OBn	TFPAA	16	-	83	17
5	OEt	TFPAA	16	-	80	20

^1^ Determined from the integral intensities of the –C(O)OC*H*_2_– signals in the ^1^H NMR spectra of product mixtures.

**Table 2:** Reaction conditions for ring opening (co)polymerization of **4c** together with characterization results of the reaction products.

Name	Monomer	Initiator	Catalyst (I:C:M Ratio) ^1^	Solvent/ Temperature	Reaction Time	Conversion/Yield (%)	*M*_theor_ (g mol^−1^)	^1^H NMR *M*_n_ (g mol^−1^)	SEC/MALS-RI		Đ
									*M*_n_ (g mol^−1^)	*M*_w_ (g mol^−1^)	
MAPCL1	**4c**	BnOH	Me_3_Al (1:1:15)	ClCH_2_CH_2_Cl/ 60 °C	5.5 h	84/50	4400	4500	5900	7100	1.20
MAPCL2	**4c**	BnOH	Me_3_Al (1:4:58)	ClCH_2_CH_2_Cl/ 60 °C	5 days	85/79	16,800	14,800	14,900	20,000	1.34
PCL-*co*-MAPCL	**4c** (25 mol%) + CL (75 mol%)	BnOH	Me_3_Al (1:4:74)	ClCH_2_CH_2_Cl/ 60 °C	16 h	98 (CL) 98 (**4c**)/93	12,300	13,100	12,800	17,100	1.34
PCL-*b*-MAPCL1	**4c**	PCL Mn = 4.3 kg mol^−1^ *Đ* = 1.03)	Me_3_Al (1:1.6:24)	ClCH_2_CH_2_Cl/ 60 °C	14 h	85/83	9800	12,400	9800	10,900	1.11
MAPCL3	**4c**	-	TU/KOMe (1:21)	CH_2_Cl_2_/RT	2 h	70/62	5000	- ^2^	4700	5400	1.15
PCL-*b*-MAPCL2	1. CL	-	TU/KOMe (44:1 (CL))	Toluene/RT	3 h	97 (CL)	4900	- ^2^	4500	5300	1.18
	2. **4c**		(12:1 (4c))	Toluene-CH_2_Cl_2_/RT		80 (**4c**)/78	8900	- ^2^	7600	8200	1.07

^1^ Initiator/catalyst/monomer ratio. ^2^ Cannot be calculated due to the overlapping of the initiator and copolymer signals in ^1^H NMR spectra.

## Data Availability

The original contributions presented in this study are included in the article or Supplementary Materials. Further inquiries can be directed to the corresponding author.

## Supplementary Materials

The following supporting information can be downloaded at https://www.mdpi.com/article/10.3390/ma18174067/s1. Supporting information contains characterization data for products including 1D and 2D NMR spectra (Figures S1–S30, S33–S35, S38–S41, S45 and S46), FTIR spectrum (Figure S31), DSC thermograms (Figures S32, S36, S42 and S47), SEC/MALS-RI chromatograms (Figures S37, S43 and S48) and DSC histograms (Figures S44 and S49).
