# Supplementary material for: Synthesis of Morpholinoamido- and Ester-Disubstituted ε-Caprolactones and Their Ring-Opening (Co)Polymerization
Source: Materials (Basel). 2025 Aug 30;18(17):4067. doi: 10.3390/ma18174067 (PMC12430100; doi:10.3390/ma18174067)
Supplement: Supplementary file 1 [file materials-18-04067-s001.zip › materials-3835602-supplementary.pdf]

Supporting Information

Synthesis of morpholinoamido- and ester-disubstituted ε-caprolactones and their ring-opening (co)polymerization

Maria Orehova [1,2,3], Ema Žagar [3] and David Pahovnik [3,*]

[1] National Institute of Chemistry, Department of Polymer Chemistry and Technology, Hajdrihova 19, 1000 Ljubljana, Slovenia;
[2] Slovenian NMR Centre, National Institute of Chemistry, Hajdrihova 19, 1000 Ljubljana, Slovenia;
[3] EN-FIST Center of Excellence, Trg Osvobodilne Fronte 13, 1000 Ljubljana, Slovenia;
* Correspondence: david.pahovnik@ki.si

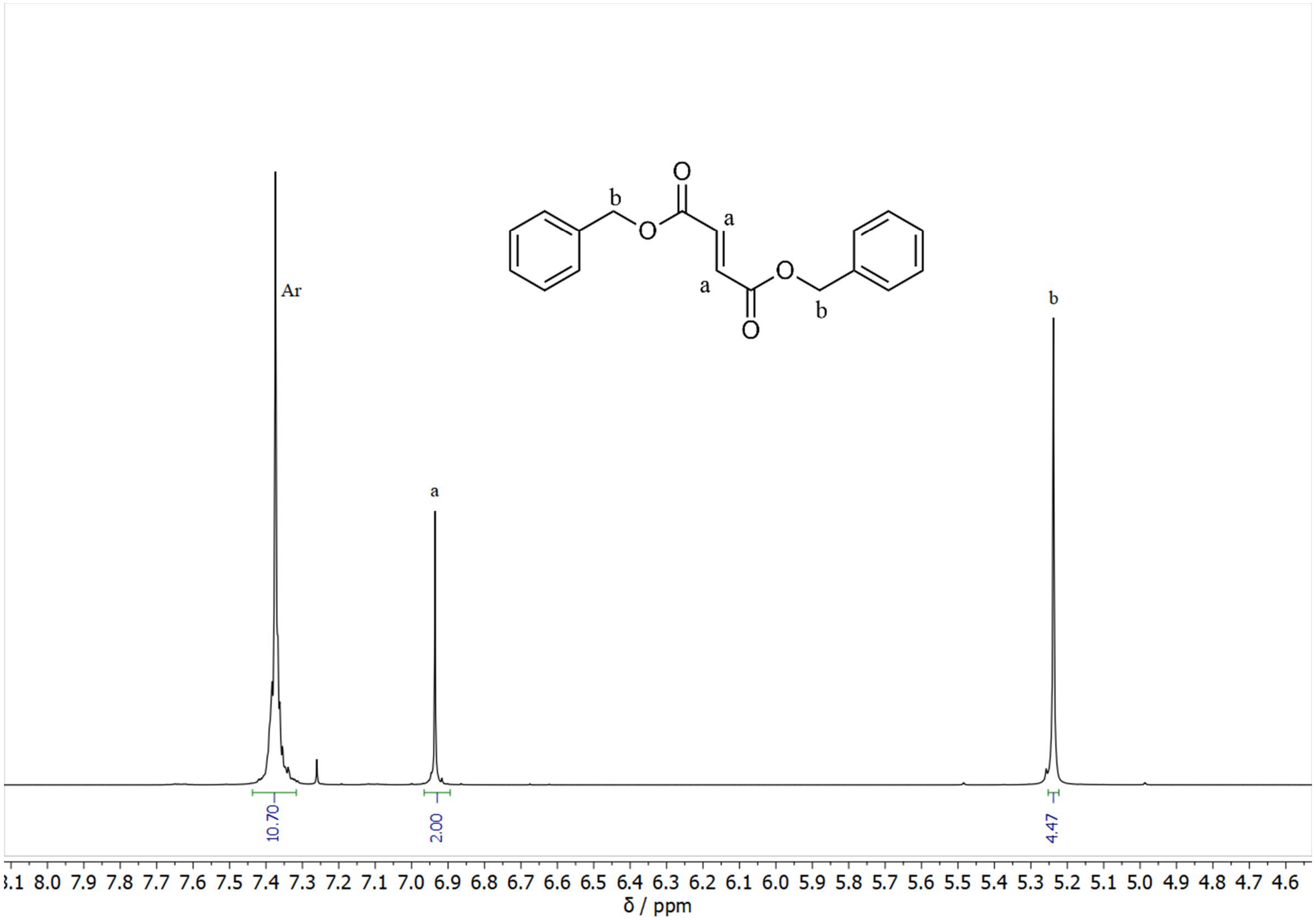

Figure S1. ^{1}H NMR spectrum of **2b** in $CDCl_3$ at 25 °C.

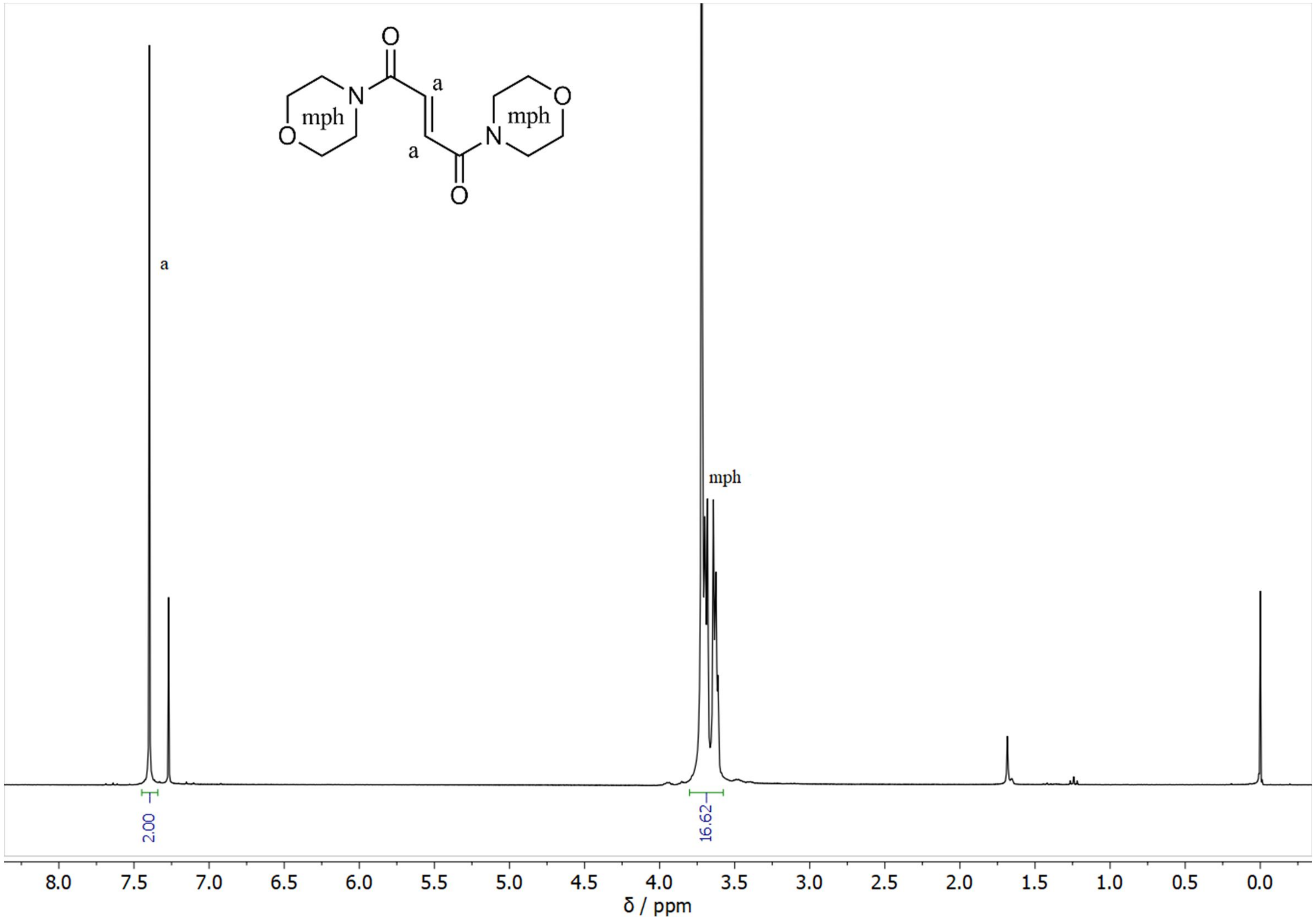

Figure S2. ^{1}H NMR spectrum of **2c** in $CDCl_3$ at 25 °C.

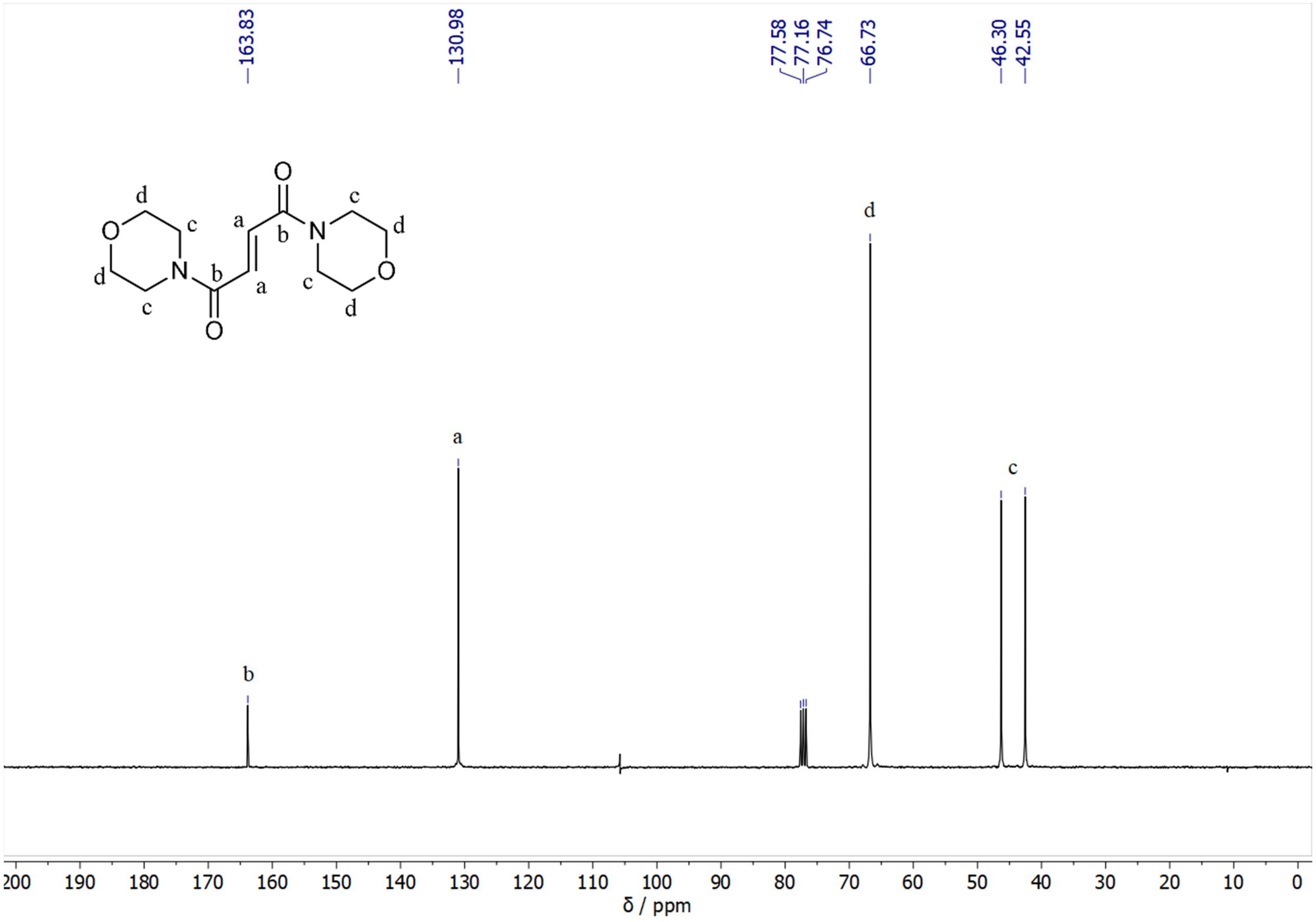

Figure S3. ^{13}C NMR spectrum of **2c** in $CDCl_3$ at 25 °C.

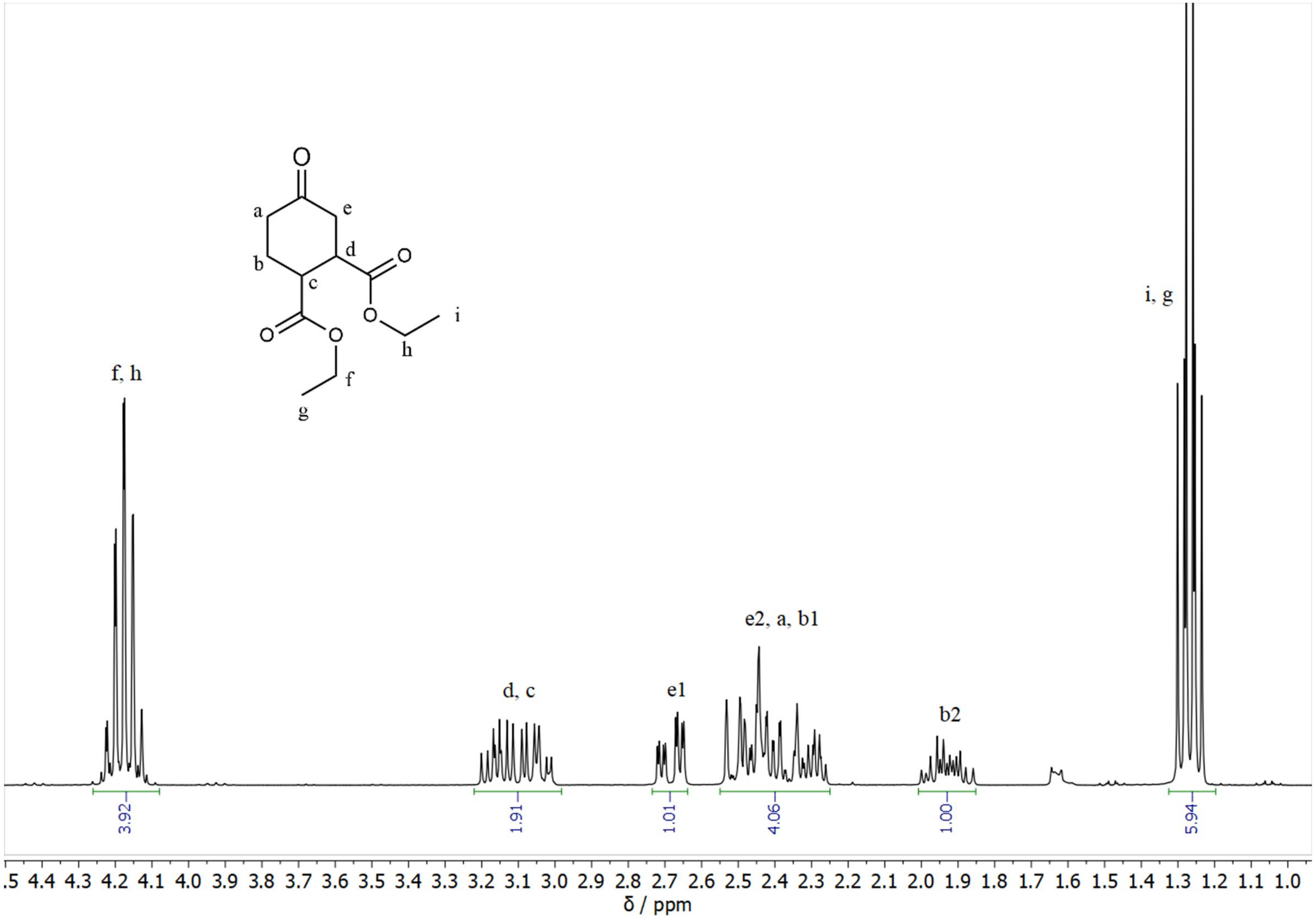

Figure S4. ^{1}H NMR spectrum of **3a** in $CDCl_3$ at 25 °C.

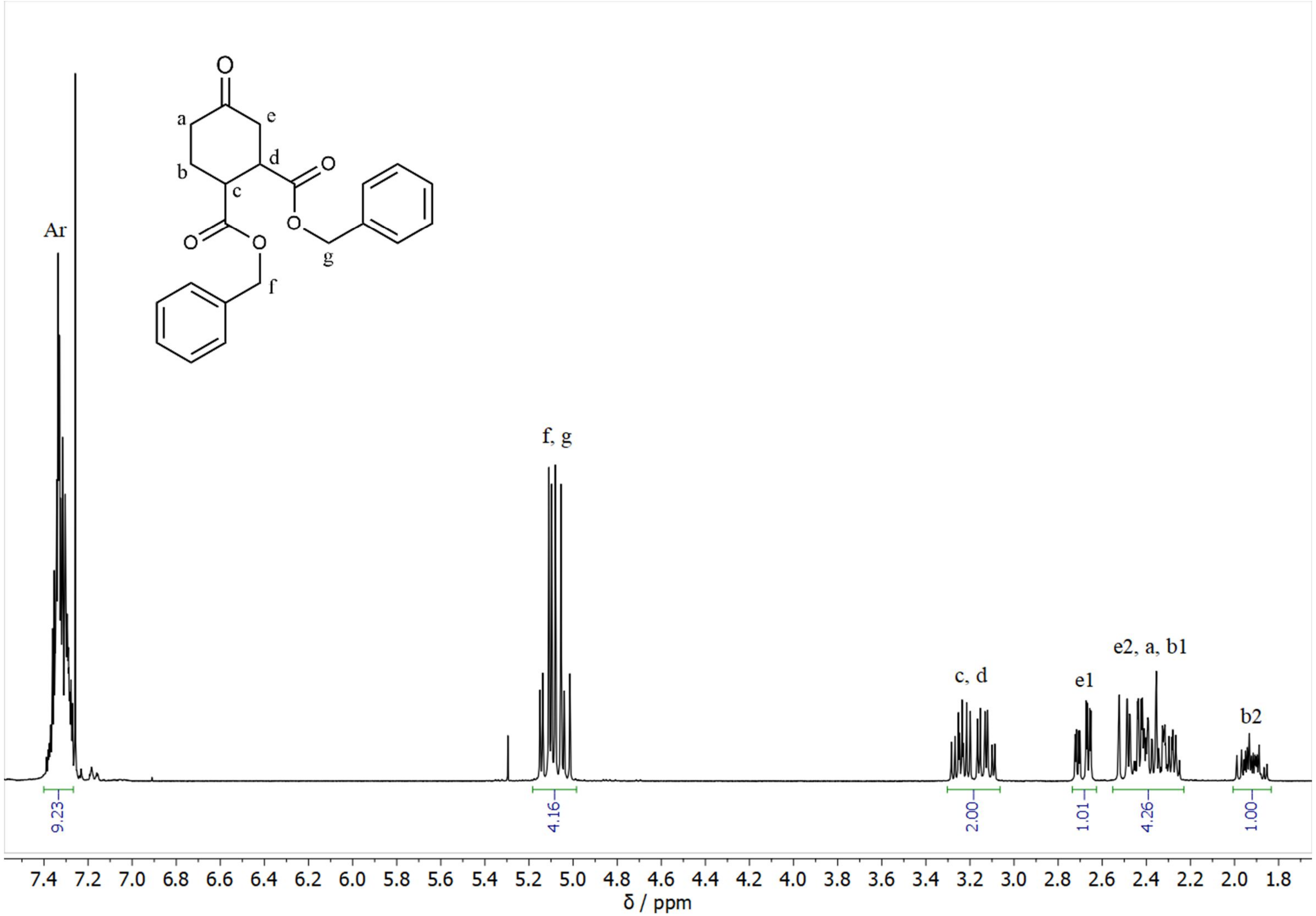

Figure S5. ^{1}H NMR spectrum of **3b** in $CDCl_3$ at 25 °C.

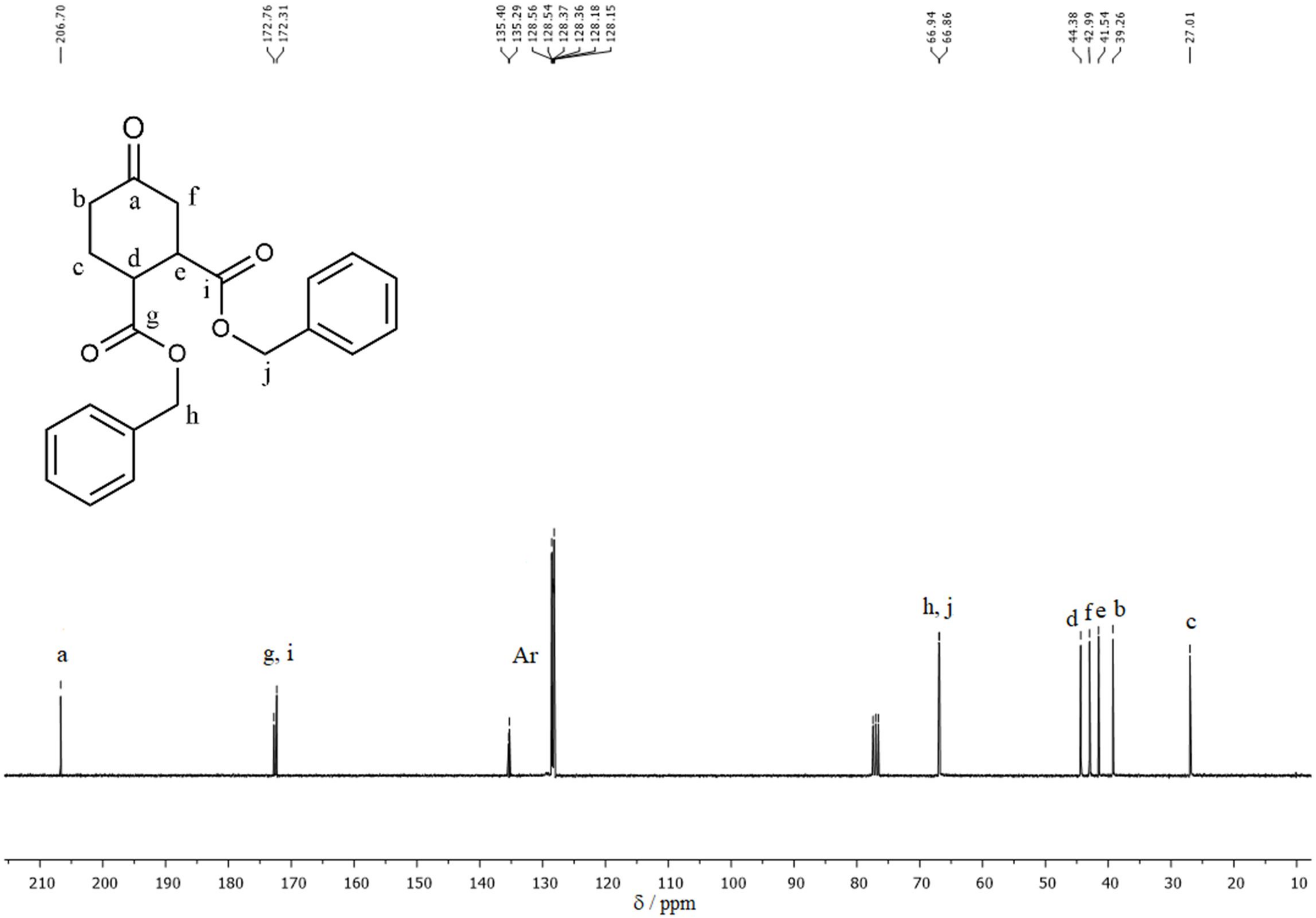

Figure S6. ^{13}C NMR spectrum of **3b** in $CDCl_3$ at 25 °C.

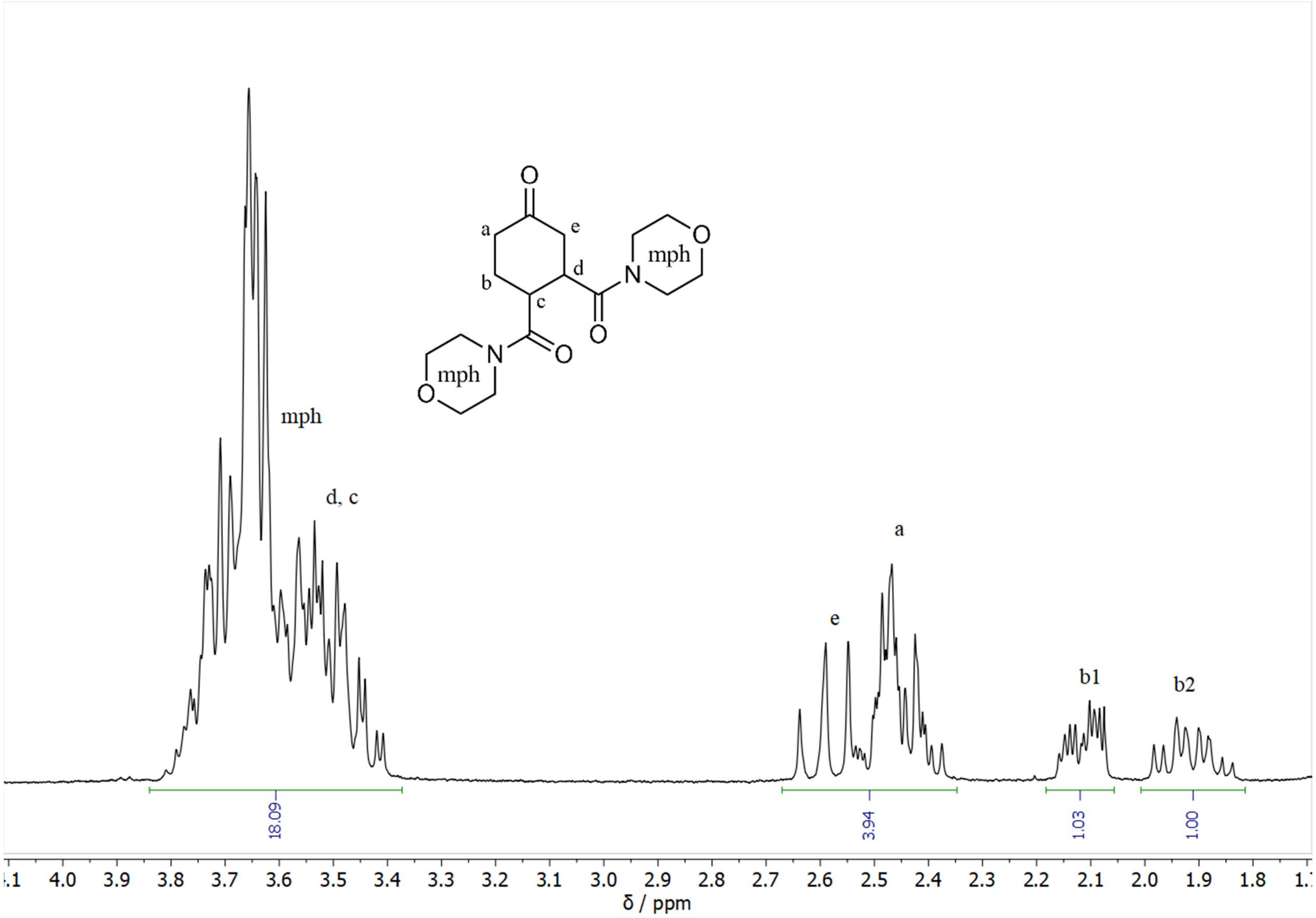

Figure S7. ^{1}H NMR spectrum of **3c** in $CDCl_3$ at 25 °C.

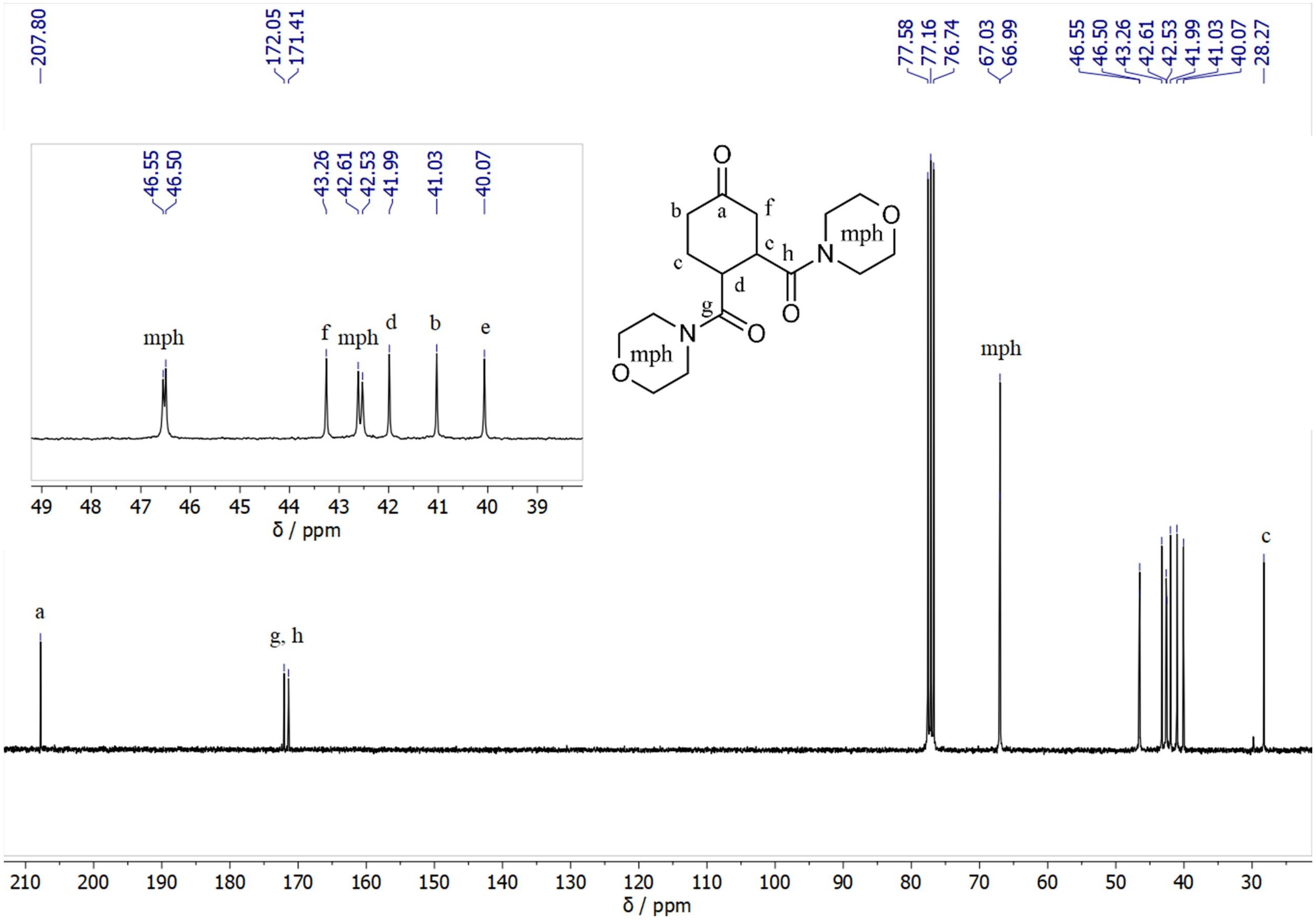

Figure S8. ^{13}C NMR spectrum of **3c** in $CDCl_3$ at 25 °C.

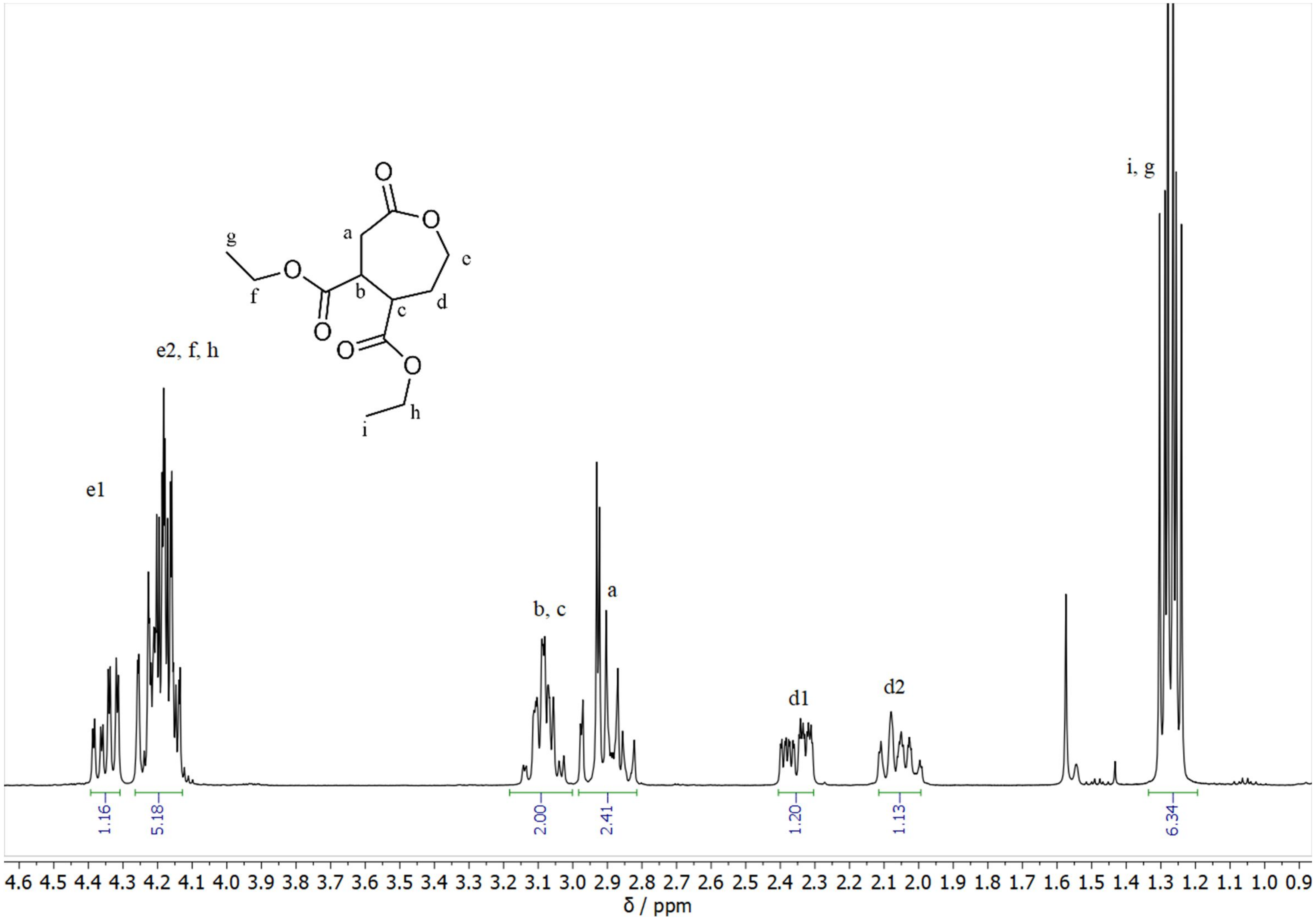

Figure S9. ^{1}H NMR spectrum of **4a** in $CDCl_3$ at 25 °C.

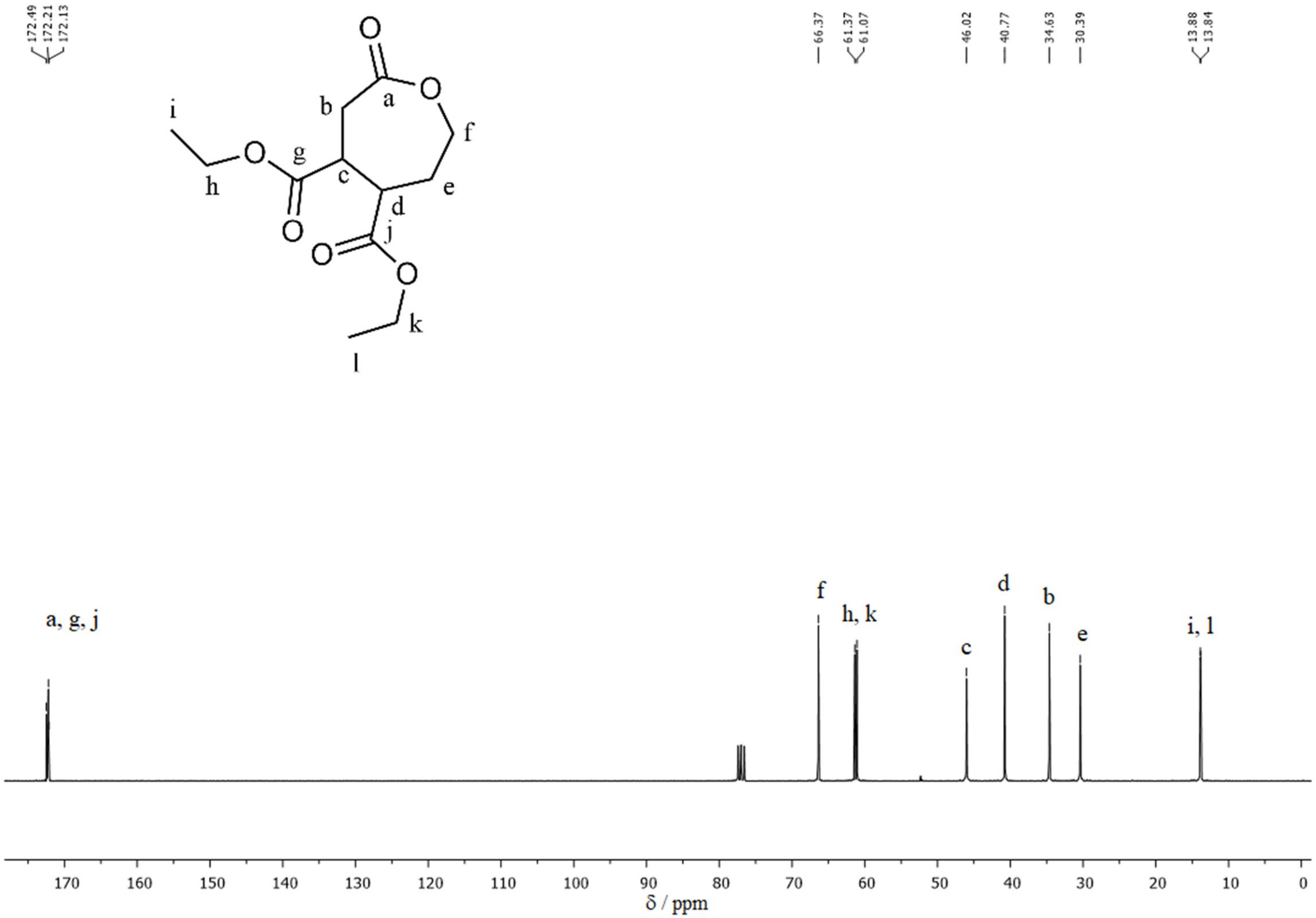

Figure S10. ^{13}C NMR spectrum of **4a** in $CDCl_3$ at 25 °C.

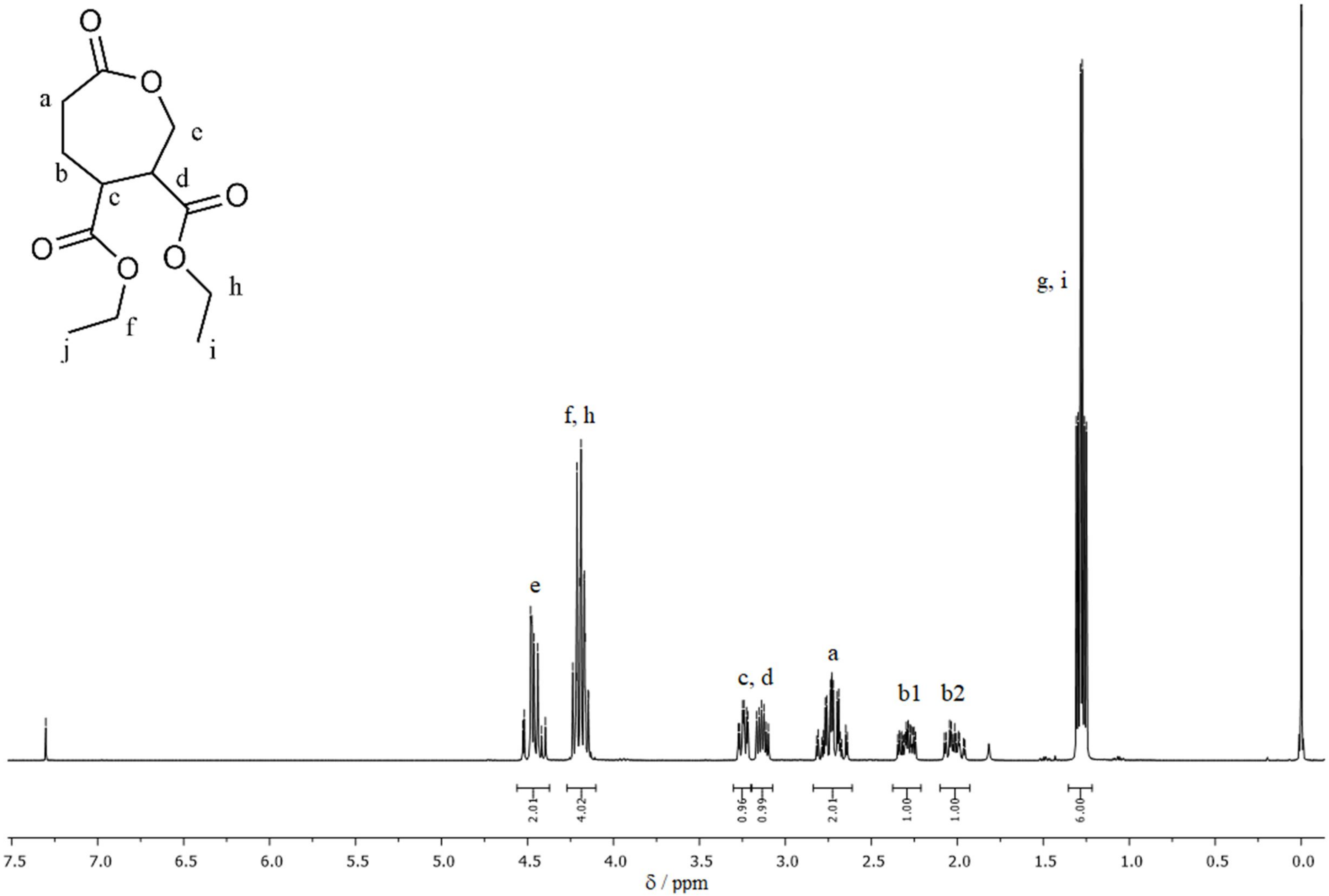

Figure S11. ^{1}H NMR spectrum of **5a** in $CDCl_3$ at 25 °C.

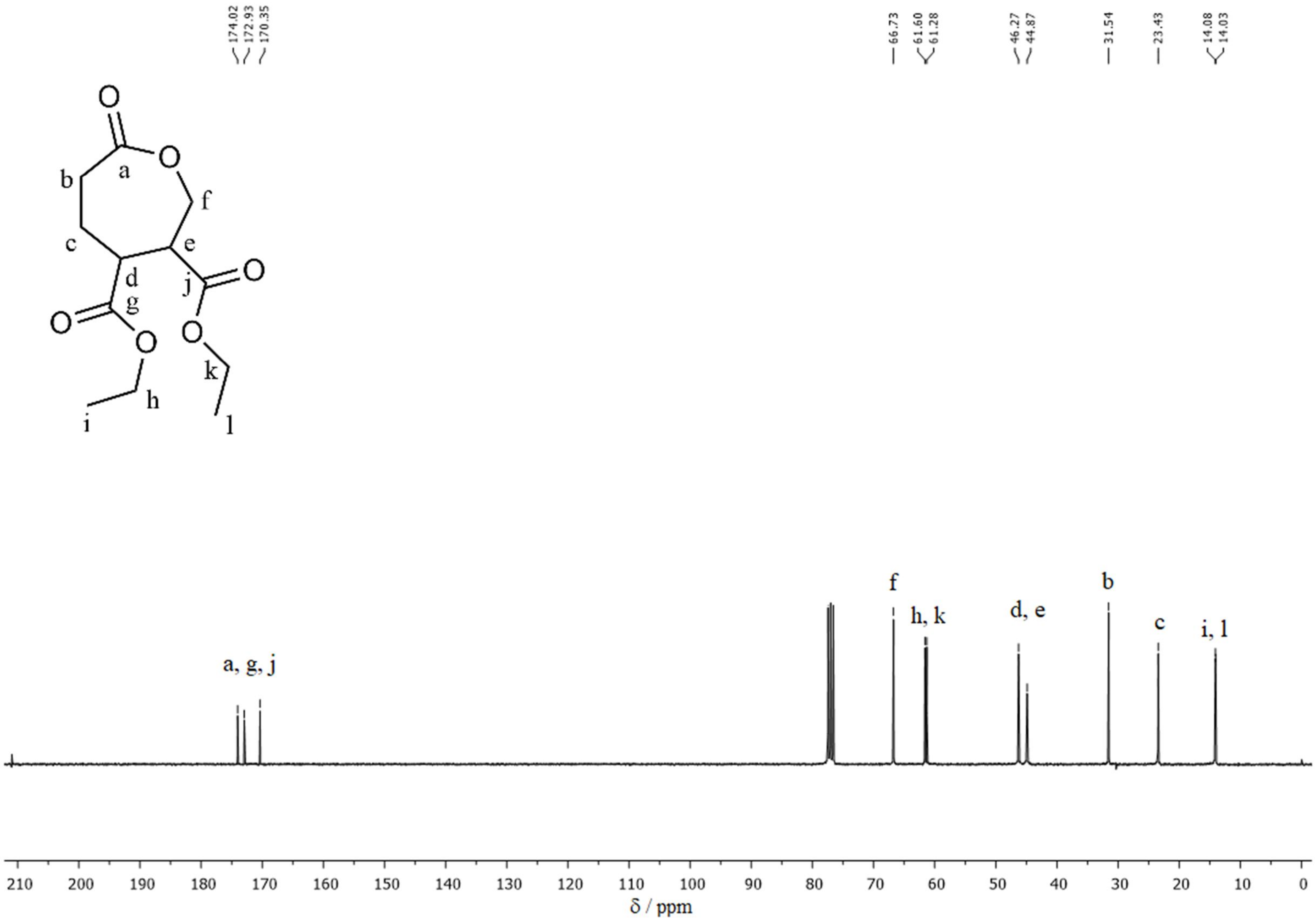

Figure S12. ^{13}C NMR spectrum of **5a** in $CDCl_3$ at 25 °C.

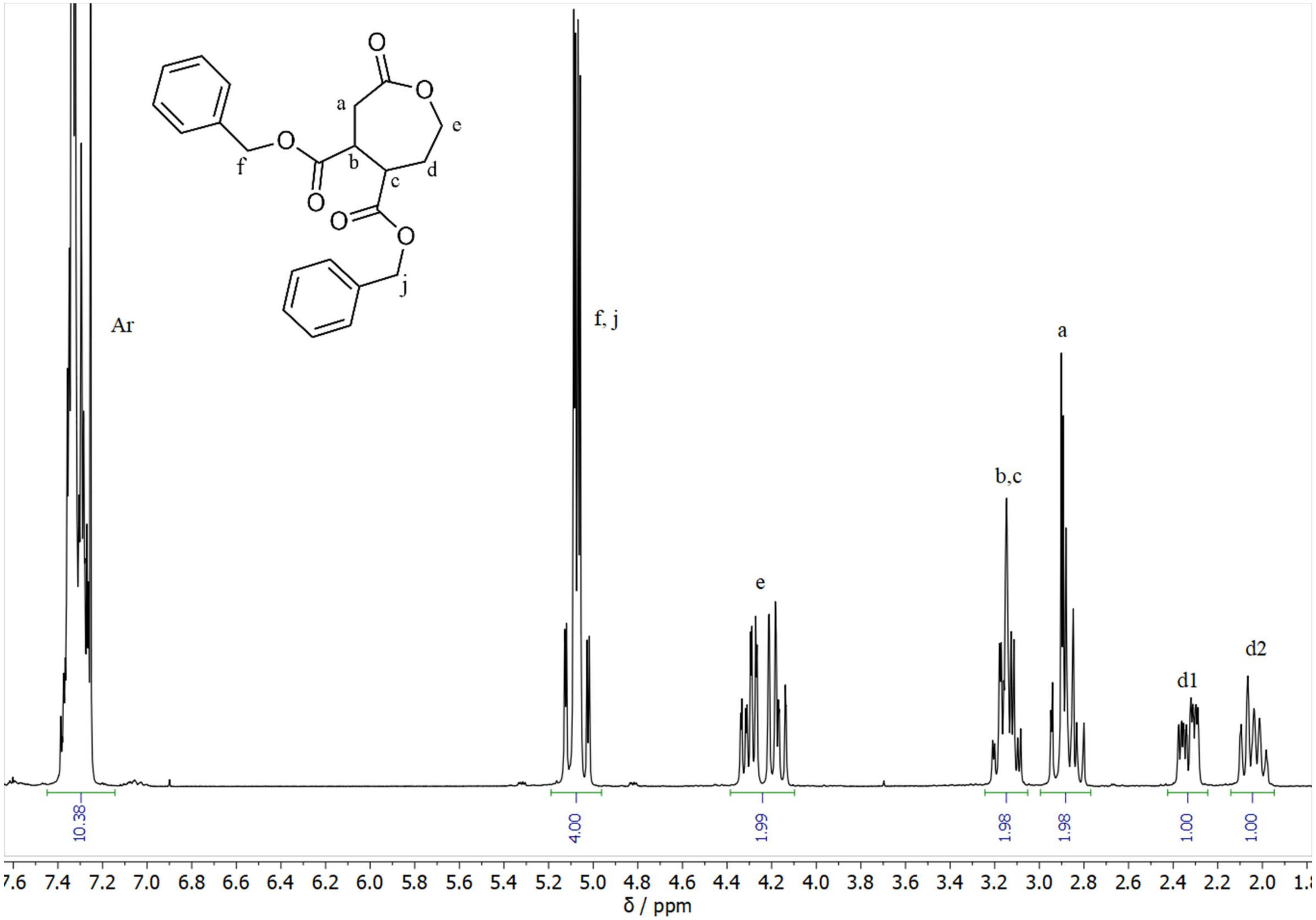

Figure S13. ^{1}H NMR spectrum of **4b** in $CDCl_3$ at 25 °C.

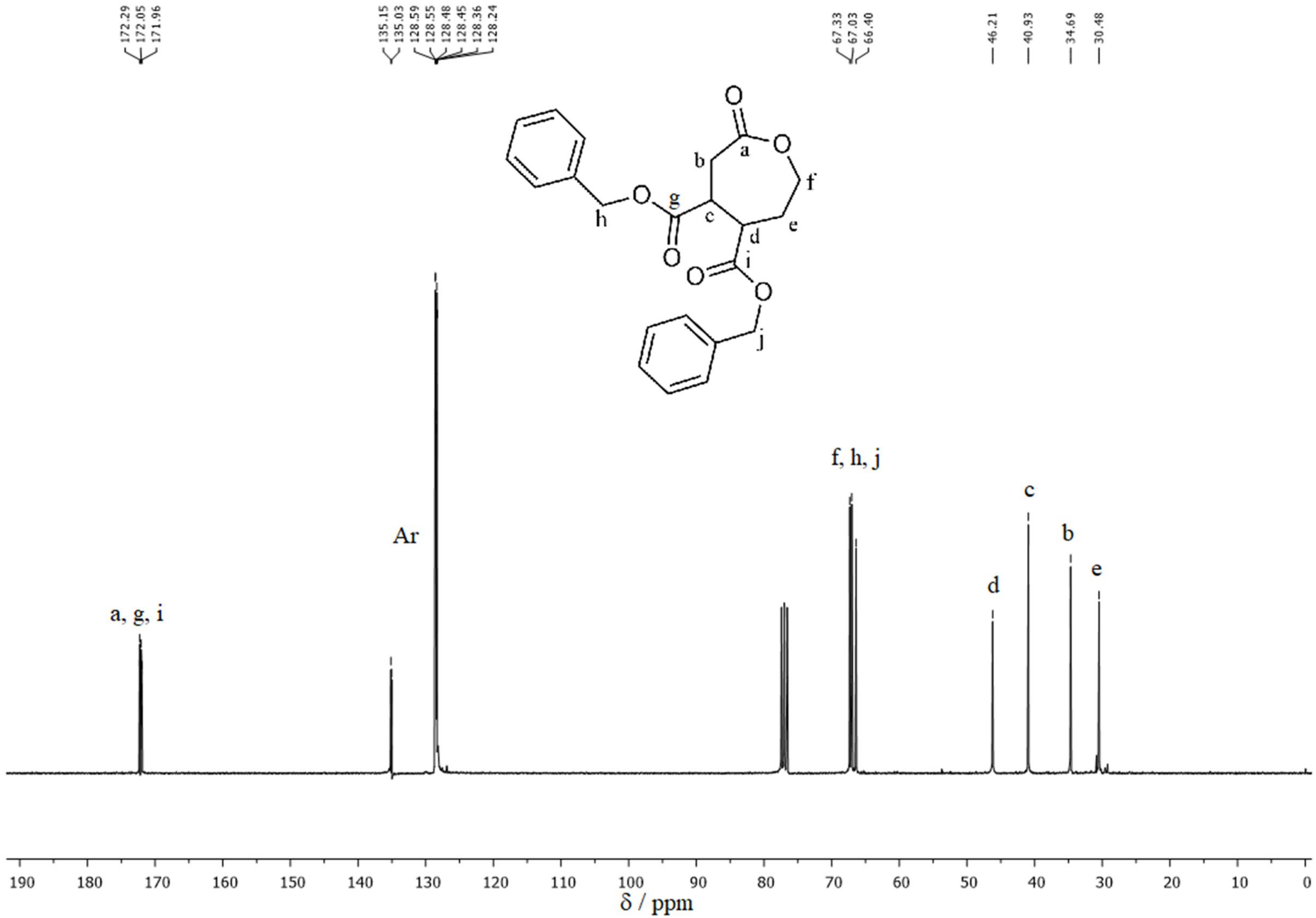

Figure S14. ^{13}C NMR spectrum of **4b** in $CDCl_3$ at 25 °C.

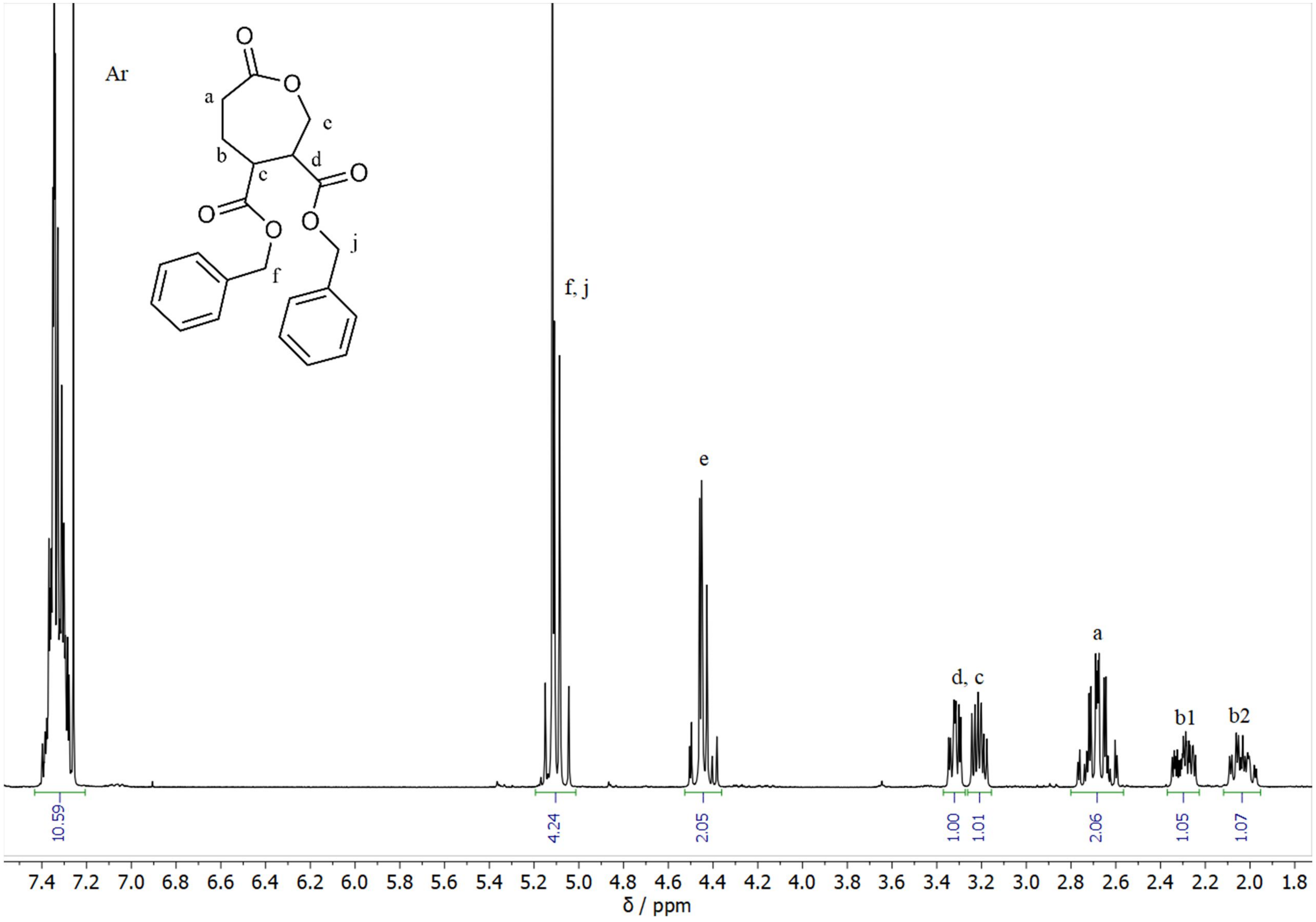

Figure S15. ^{1}H NMR spectrum of **5b** in $CDCl_3$ at 25 °C.

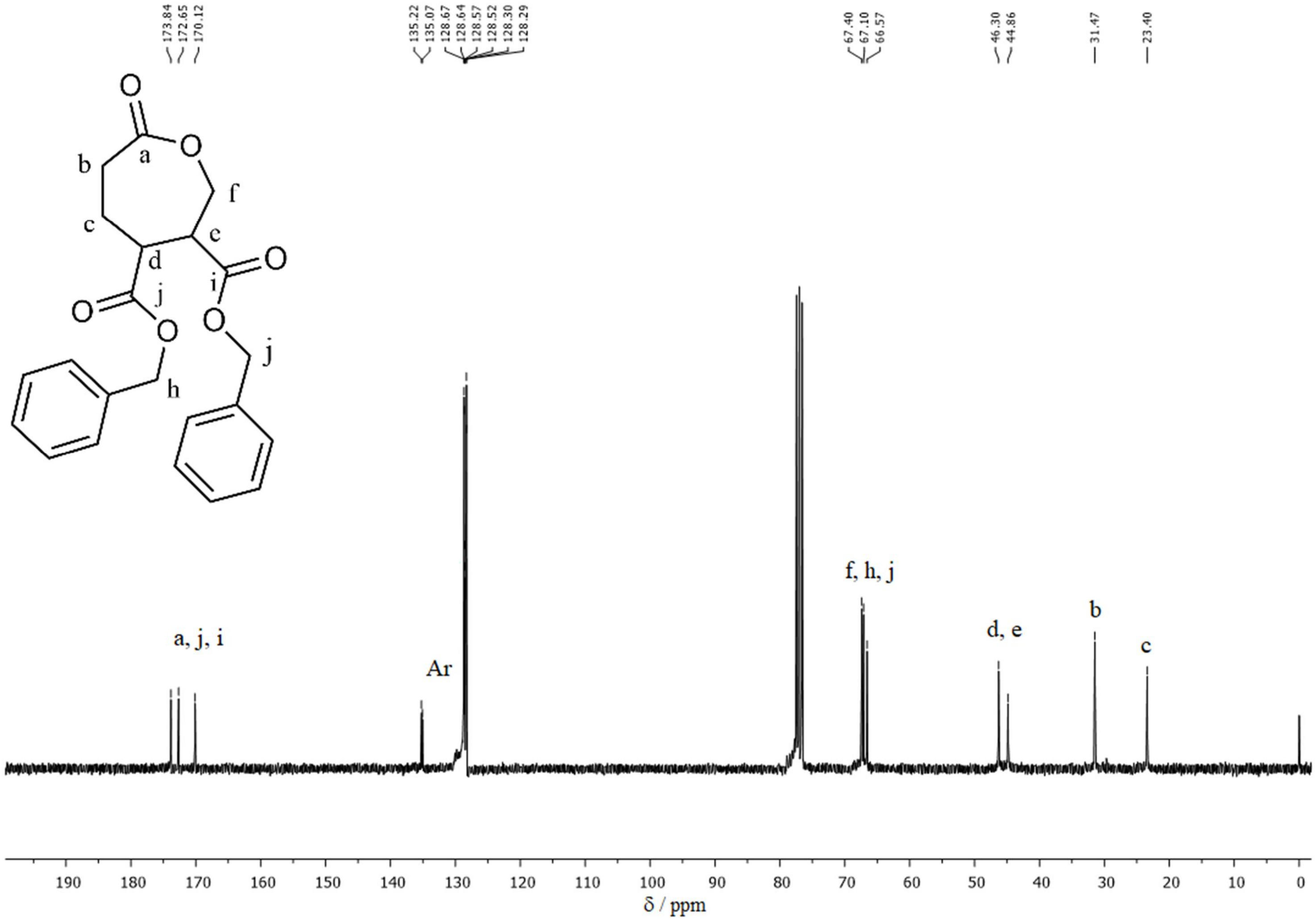

Figure S16. ^{13}C NMR spectrum of **5b** in $CDCl_3$ at 25 °C.

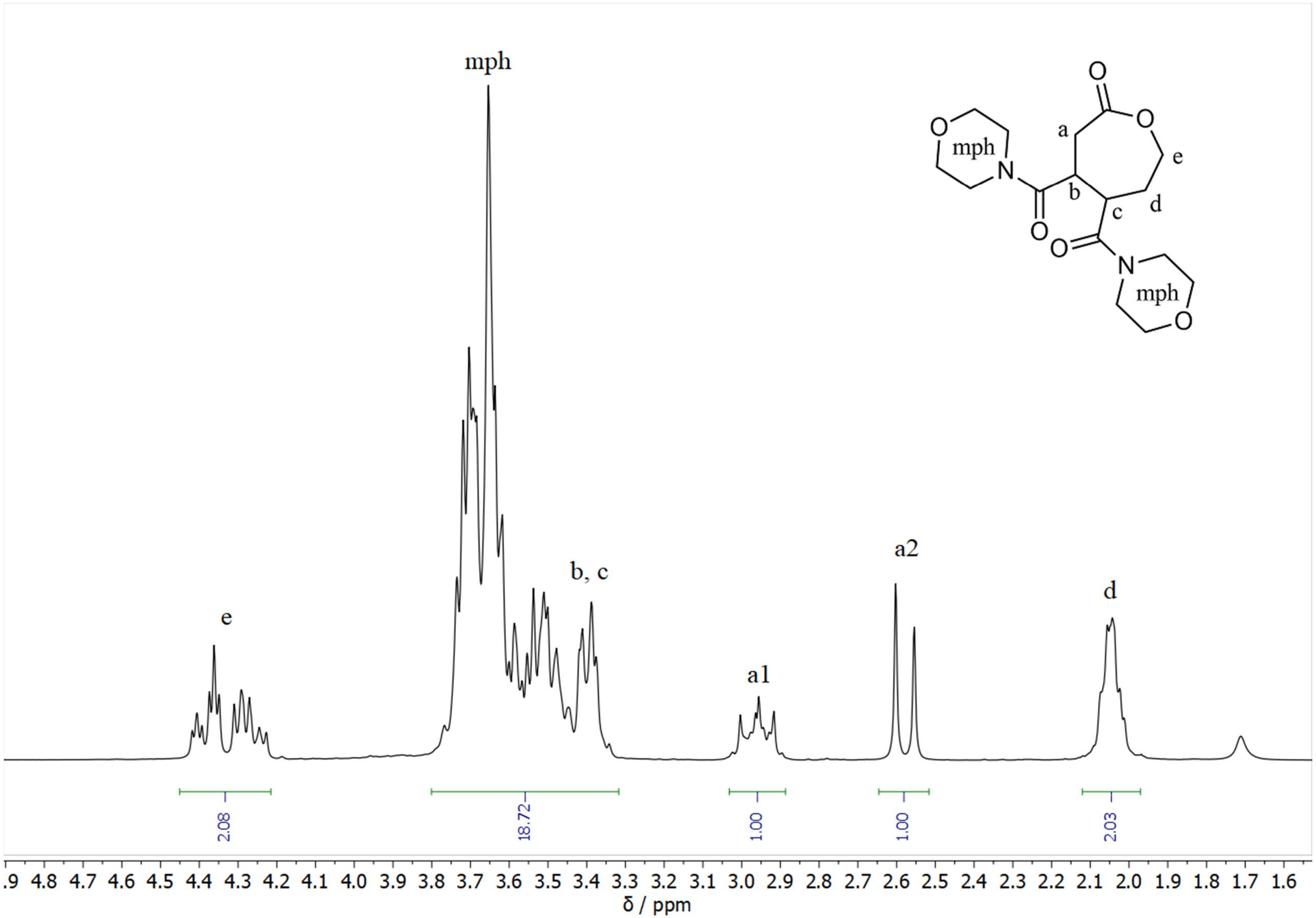

Figure S17. ^{1}H NMR spectrum of **4c** in $CDCl_3$ at 25 °C.

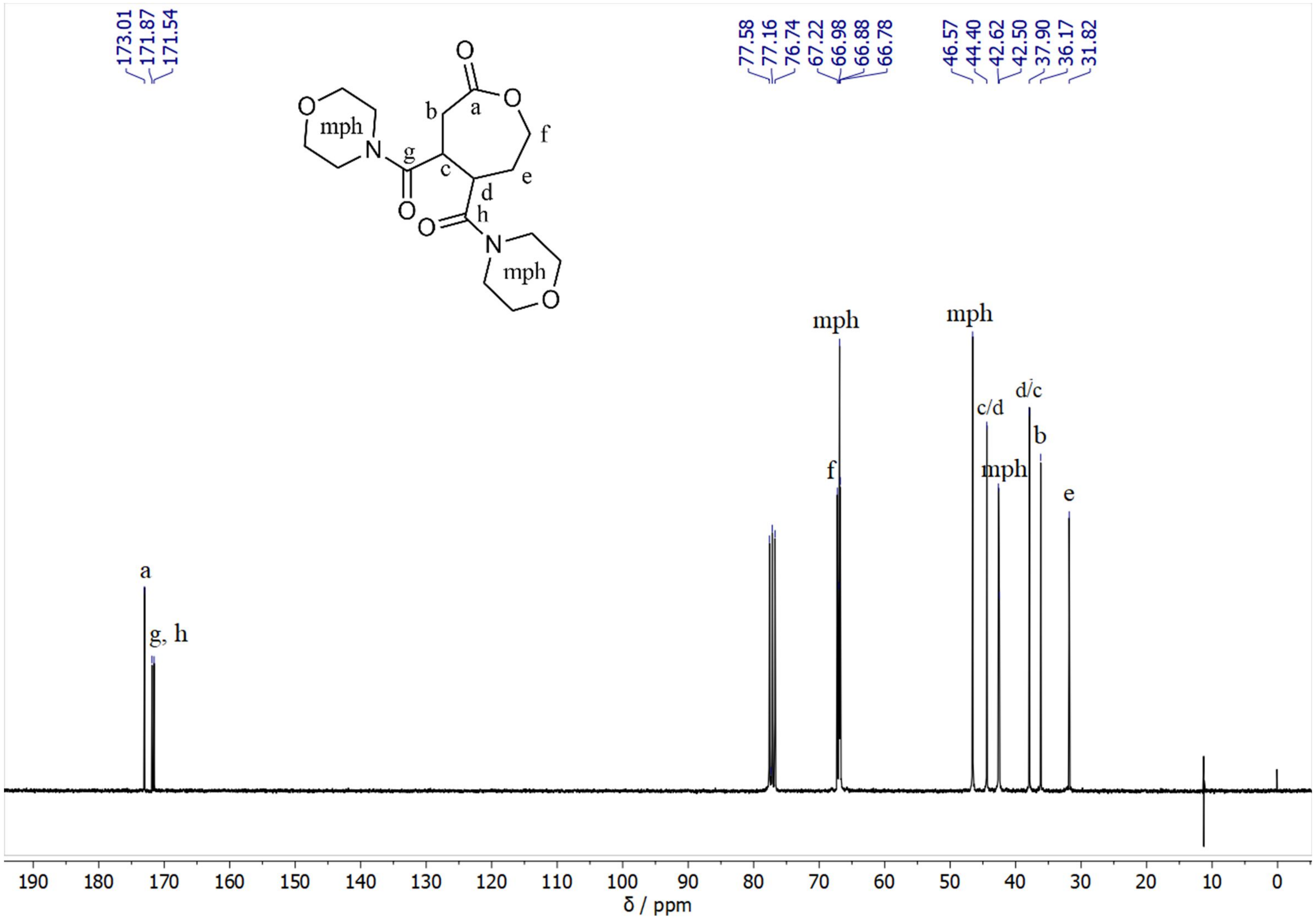

Figure S18. ^{13}C NMR spectrum of **4c** in $CDCl_3$ at 25 °C.

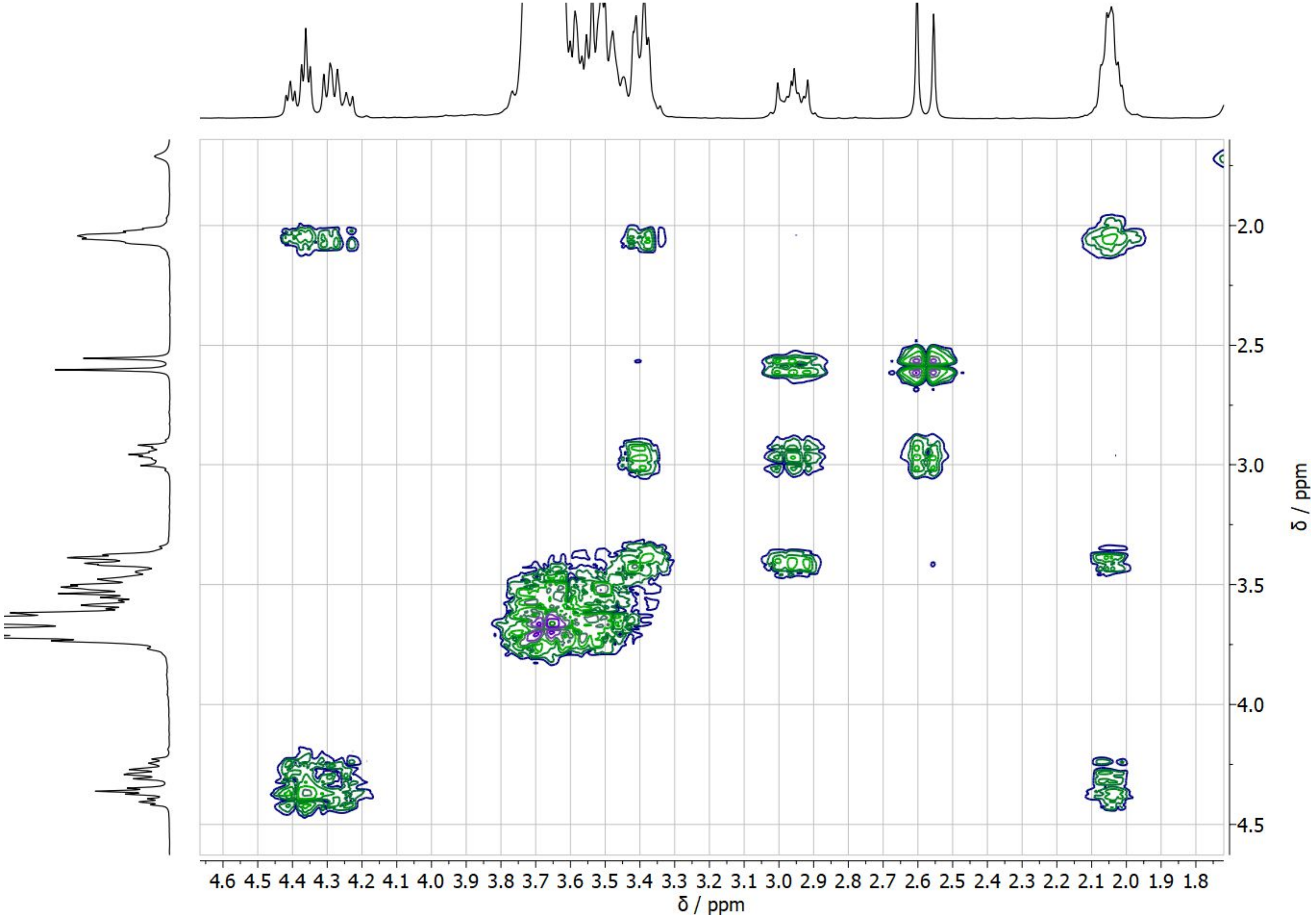

Figure S19. ^{1}H-^{1}H COSY NMR spectrum of **4c** in $CDCl_3$ at 25 °C.

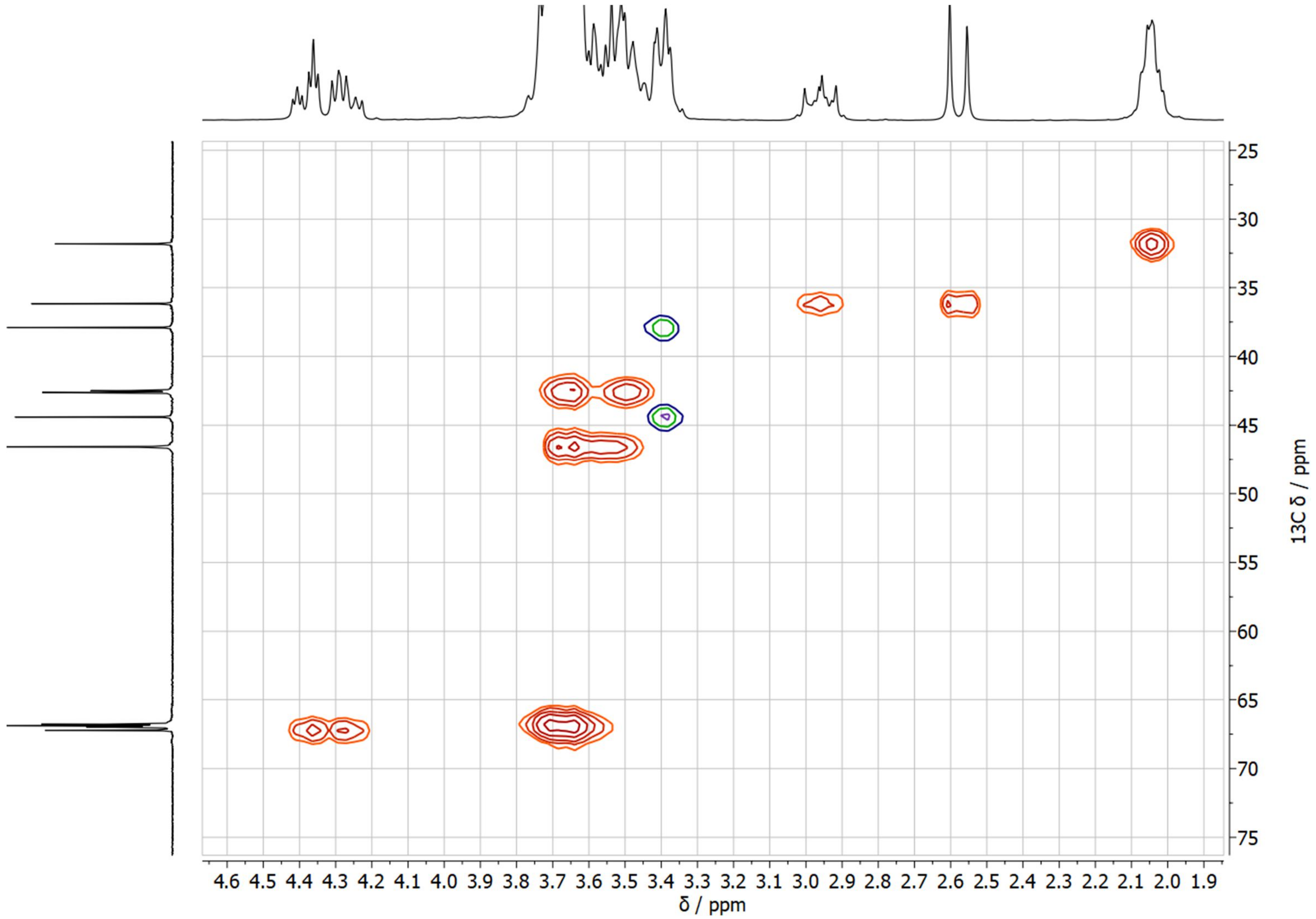

Figure S20. ^{1}H-^{13}C HSQC NMR spectrum of **4c** in $CDCl_3$ at 25 °C.

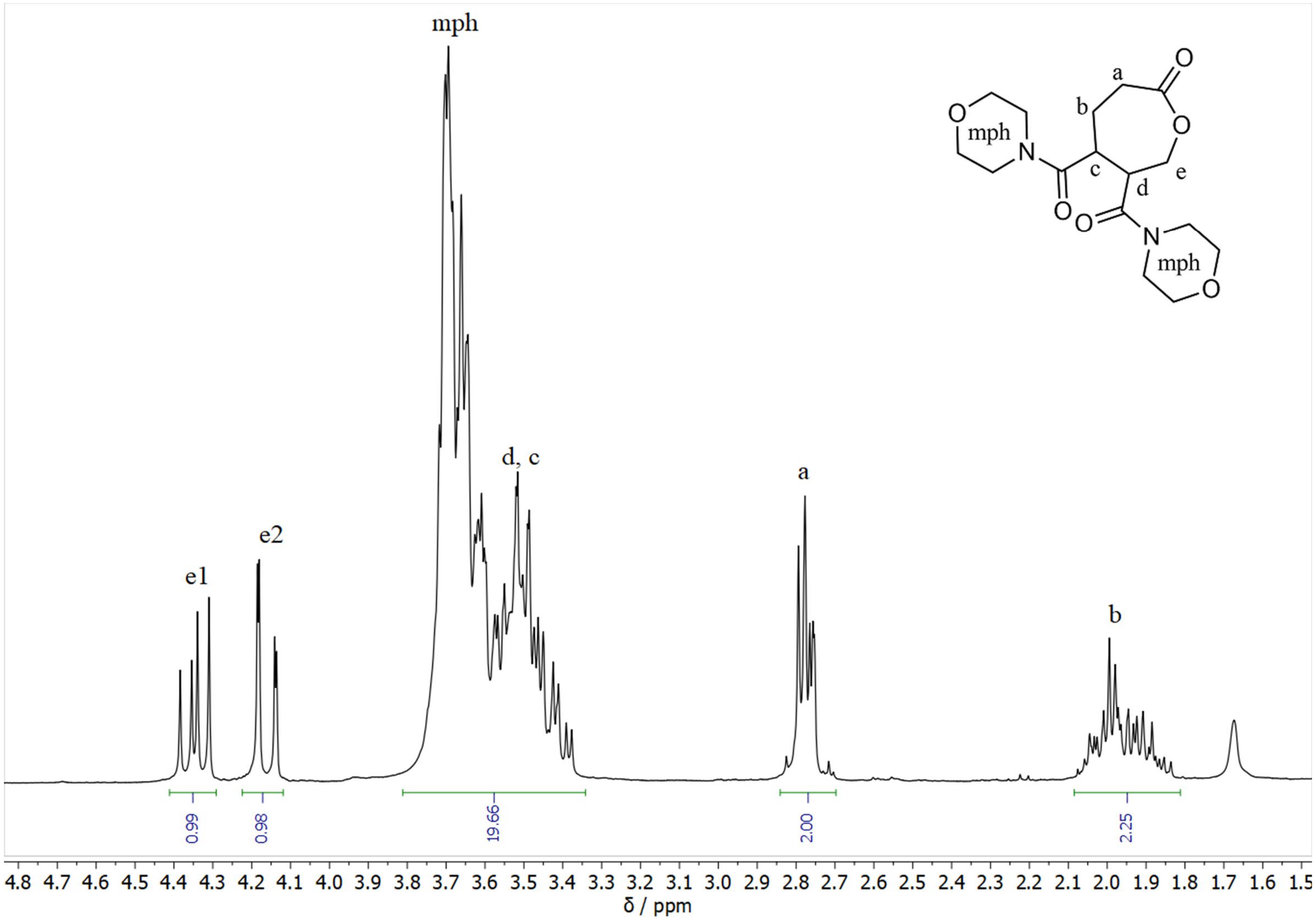

Figure S21. ^{1}H NMR spectrum of **5c** in $CDCl_3$ at 25 °C.

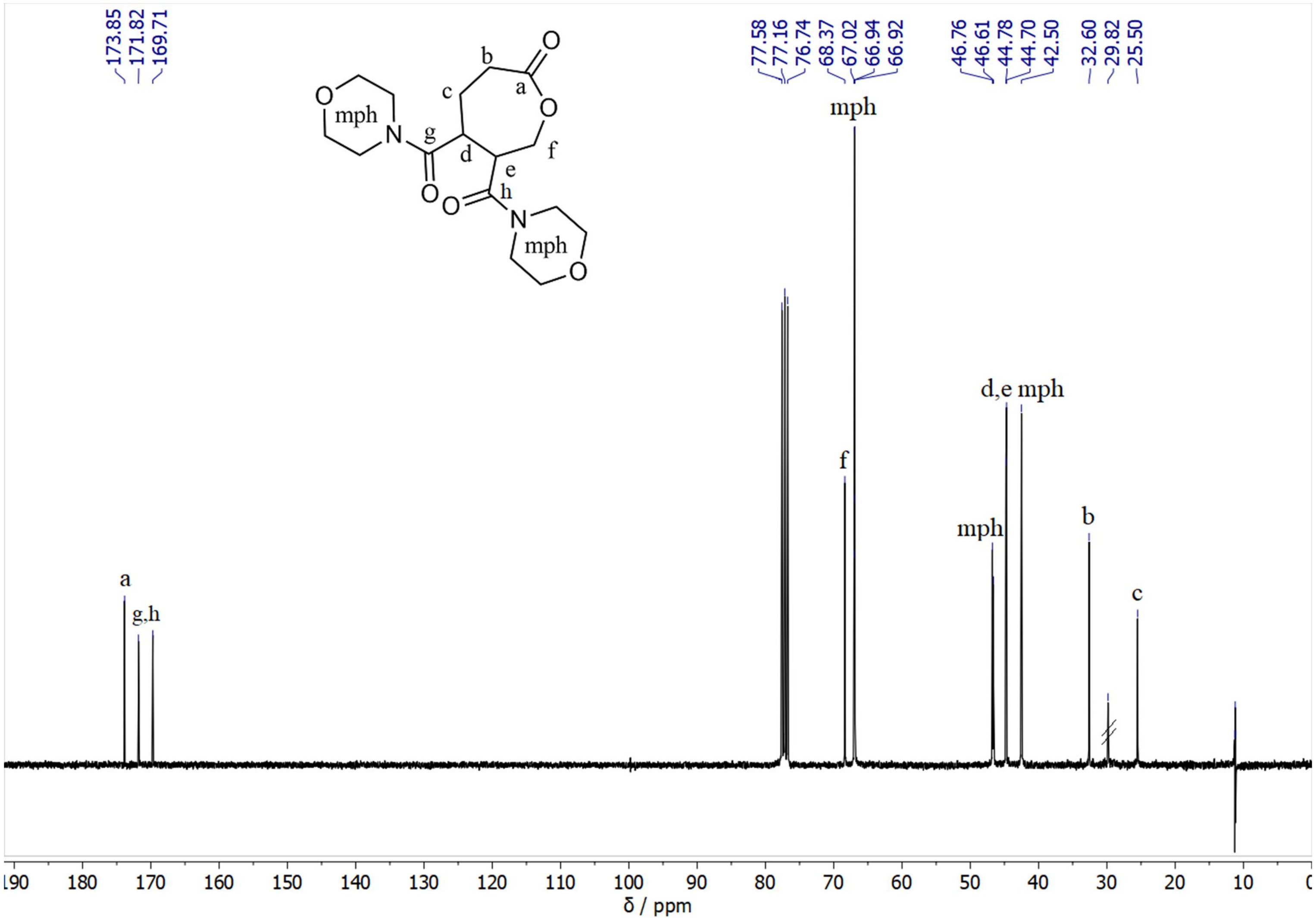

Figure S22. ^{13}C NMR spectrum of **5c** in $CDCl_3$ at 25 °C.

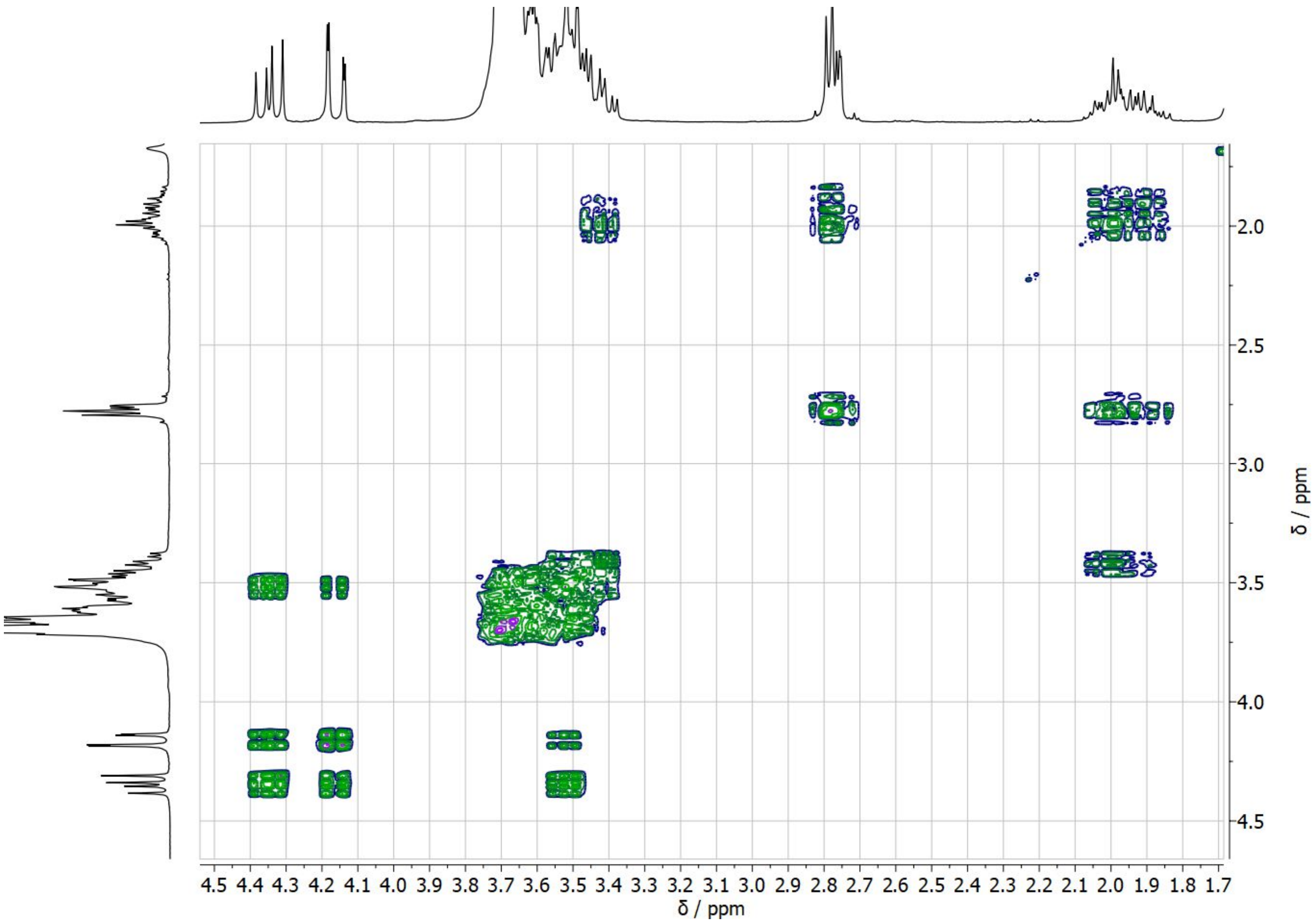

Figure S23. ^{1}H-^{1}H COSY NMR spectrum of **5c** in $CDCl_3$ at 25 °C.

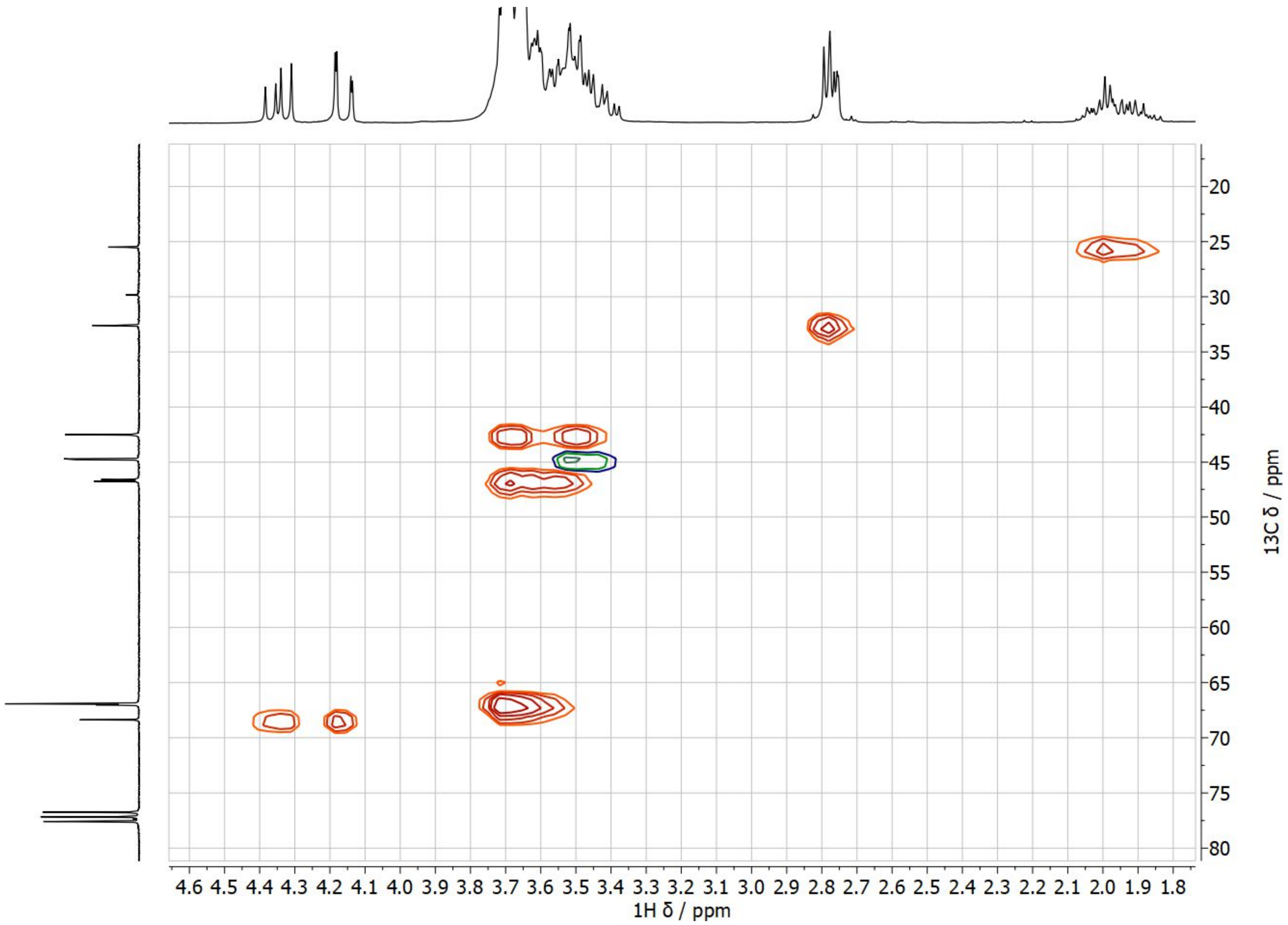

Figure S24. ^{1}H-^{13}C HSQC NMR spectrum of **5c** in $CDCl_3$ at 25 °C.

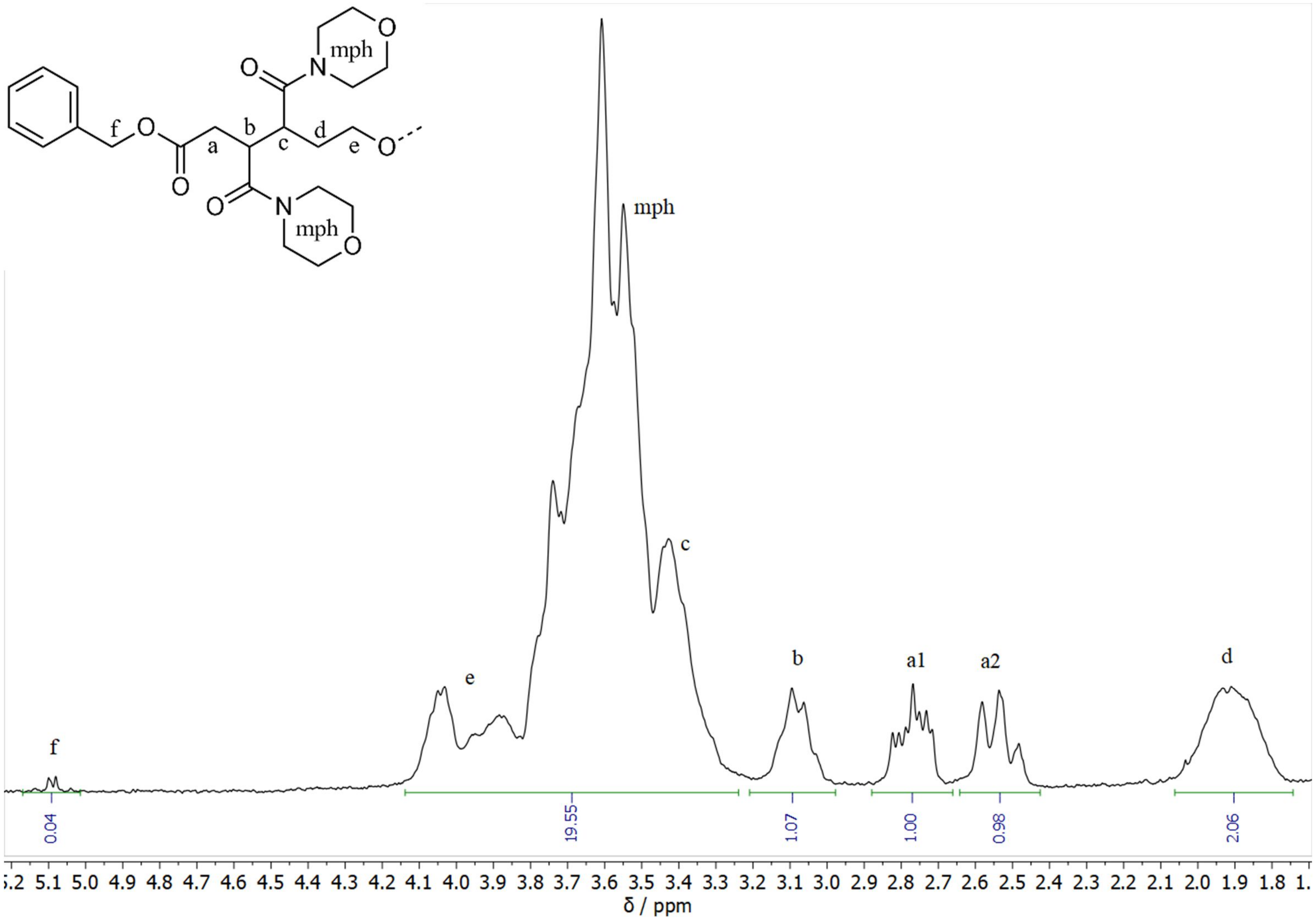

Figure S25. ^{1}H NMR spectrum of MAPCL2 homopolymer in $CDCl_3$ at 25 °C.

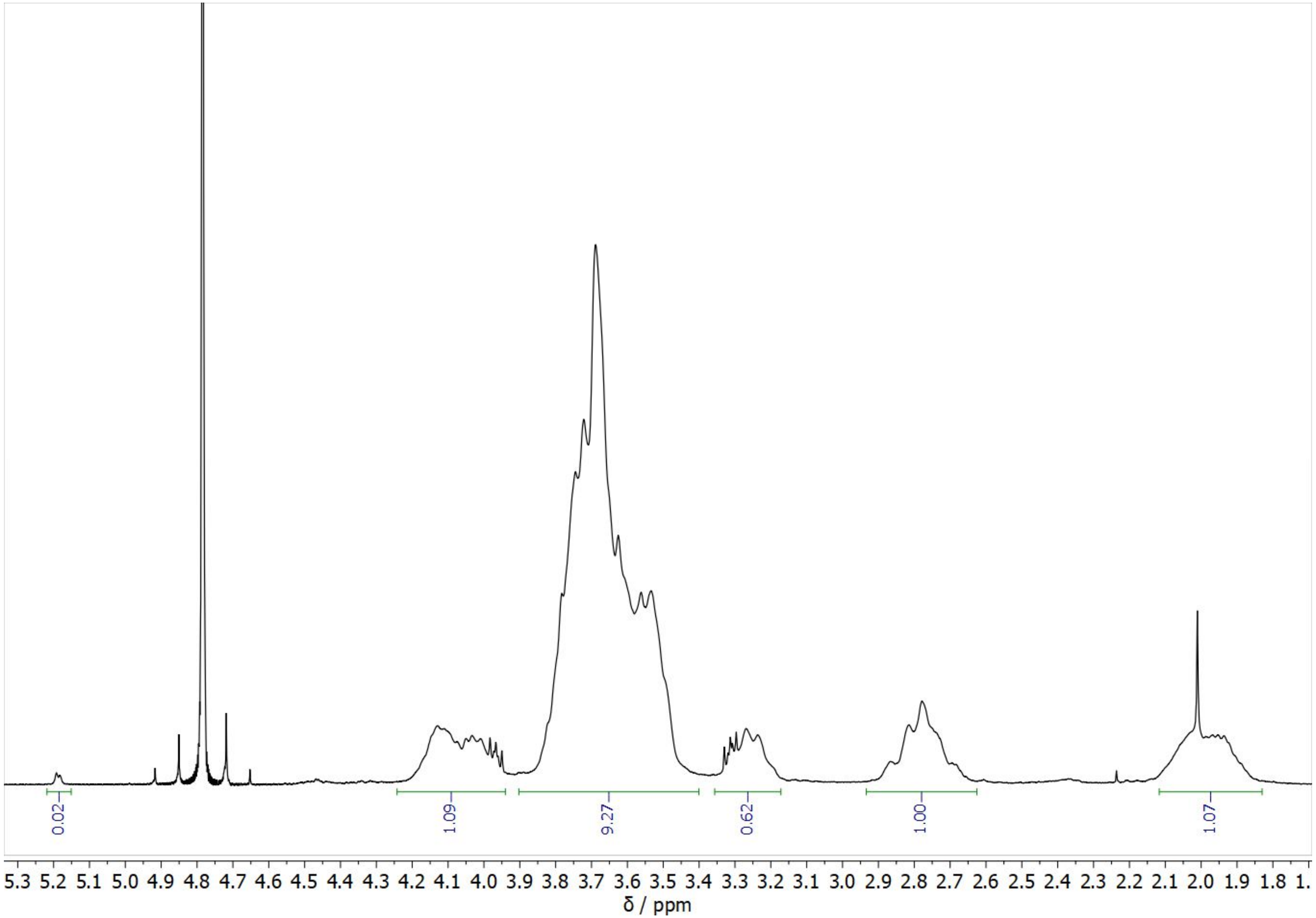

Figure S26. ^1H NMR spectrum of MAPCL2 homopolymer in D_2O at 25 °C.

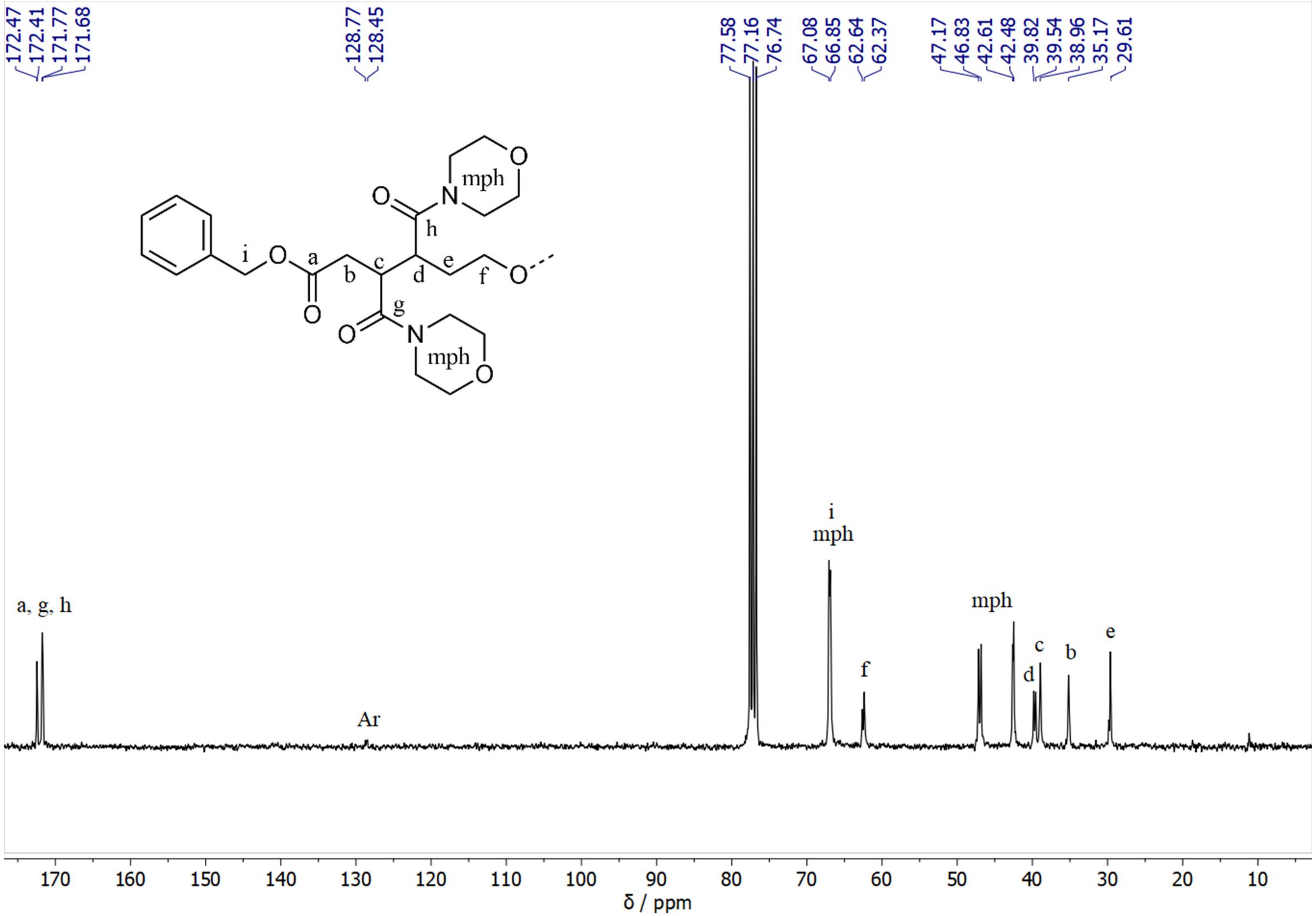

Figure S27. ^{13}C NMR spectrum of MAPCL2 homopolymer in $CDCl_3$ at 25 °C.

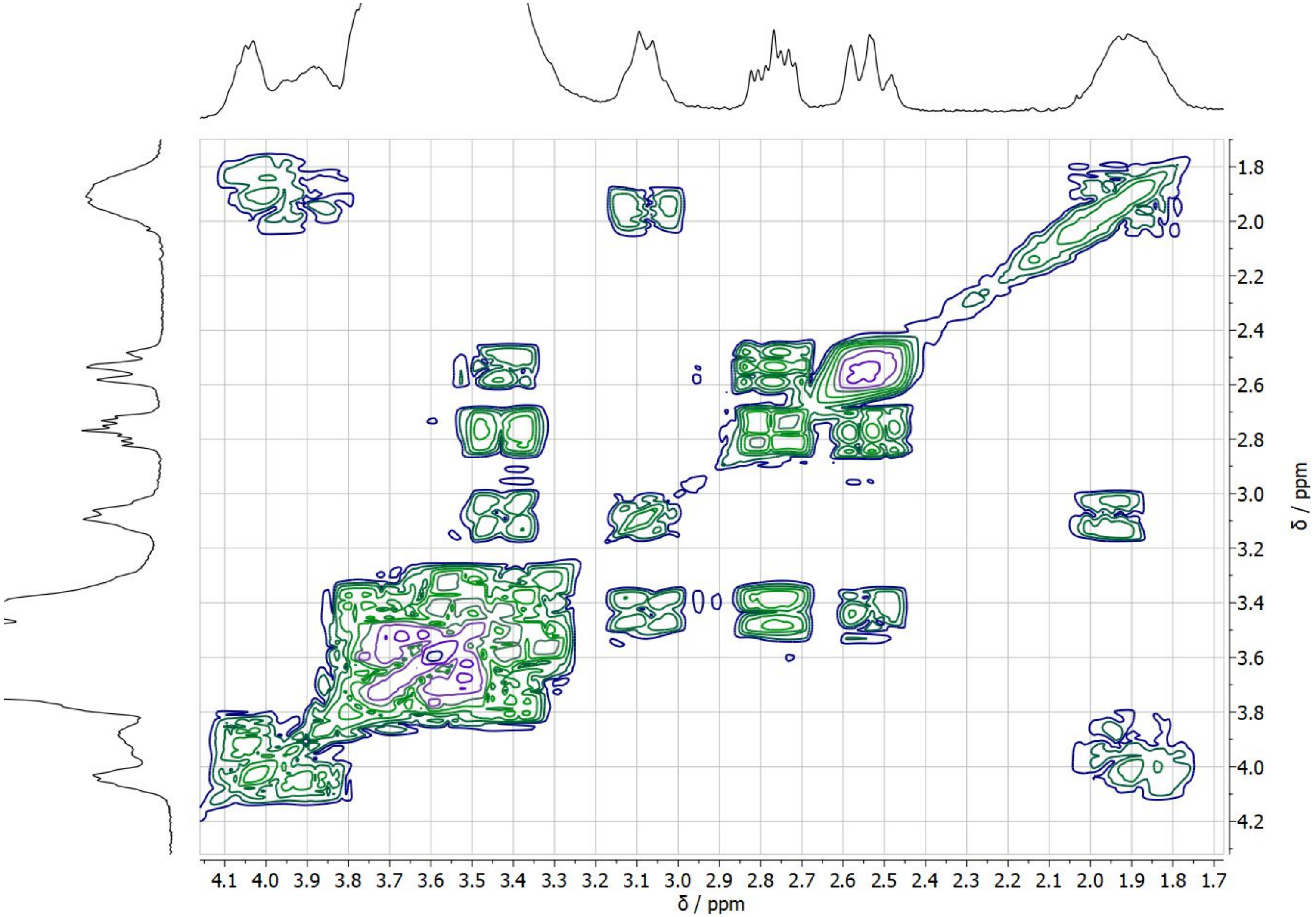

Figure S28. ^1H-^1H COSY NMR spectrum of MAPCL2 homopolymer in $CDCl_3$ at 25 °C.

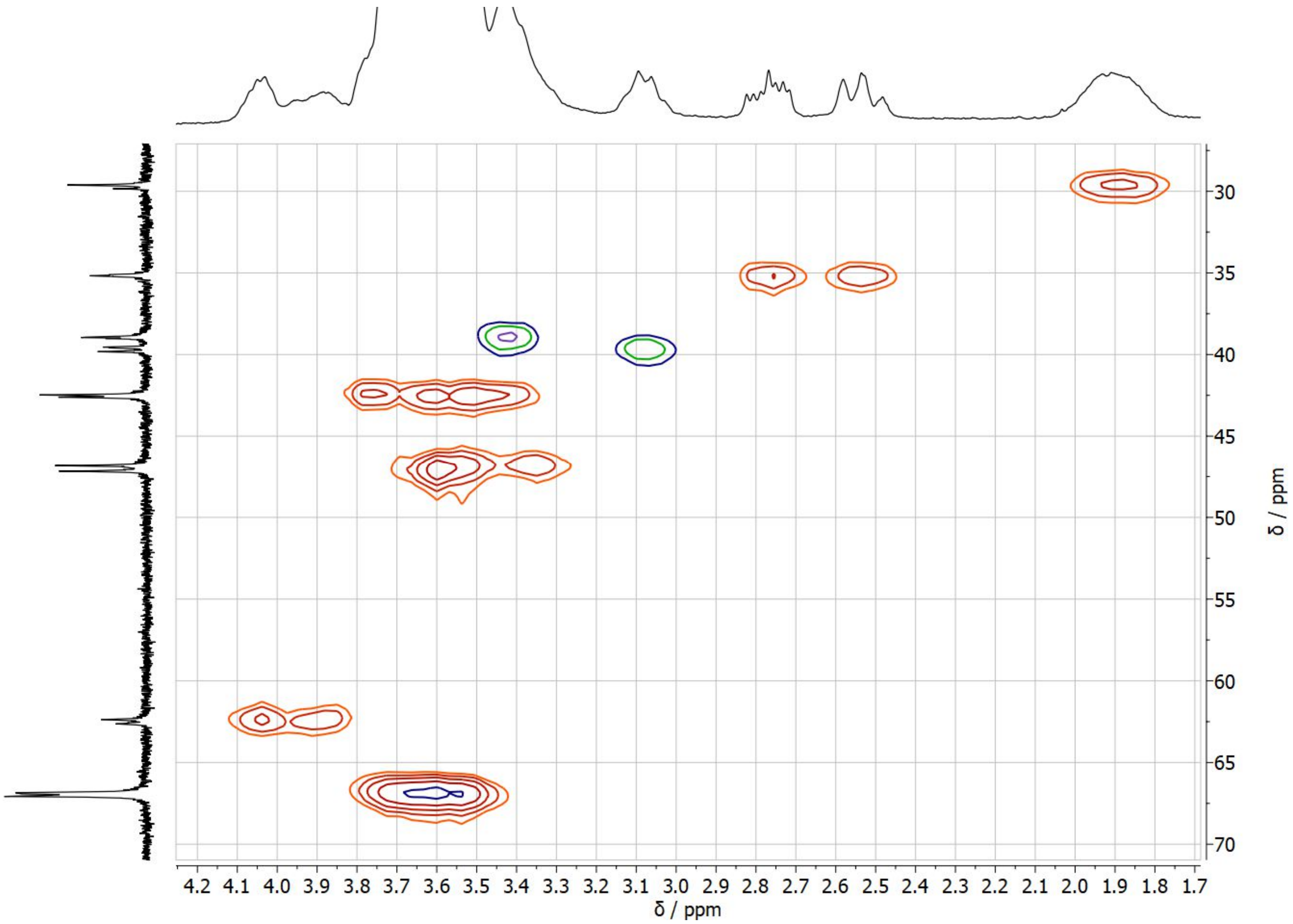

Figure S29. ^{1}H-^{13}C HSQC NMR spectrum of MAPCL2 homopolymer in $CDCl_3$ at 25 °C.

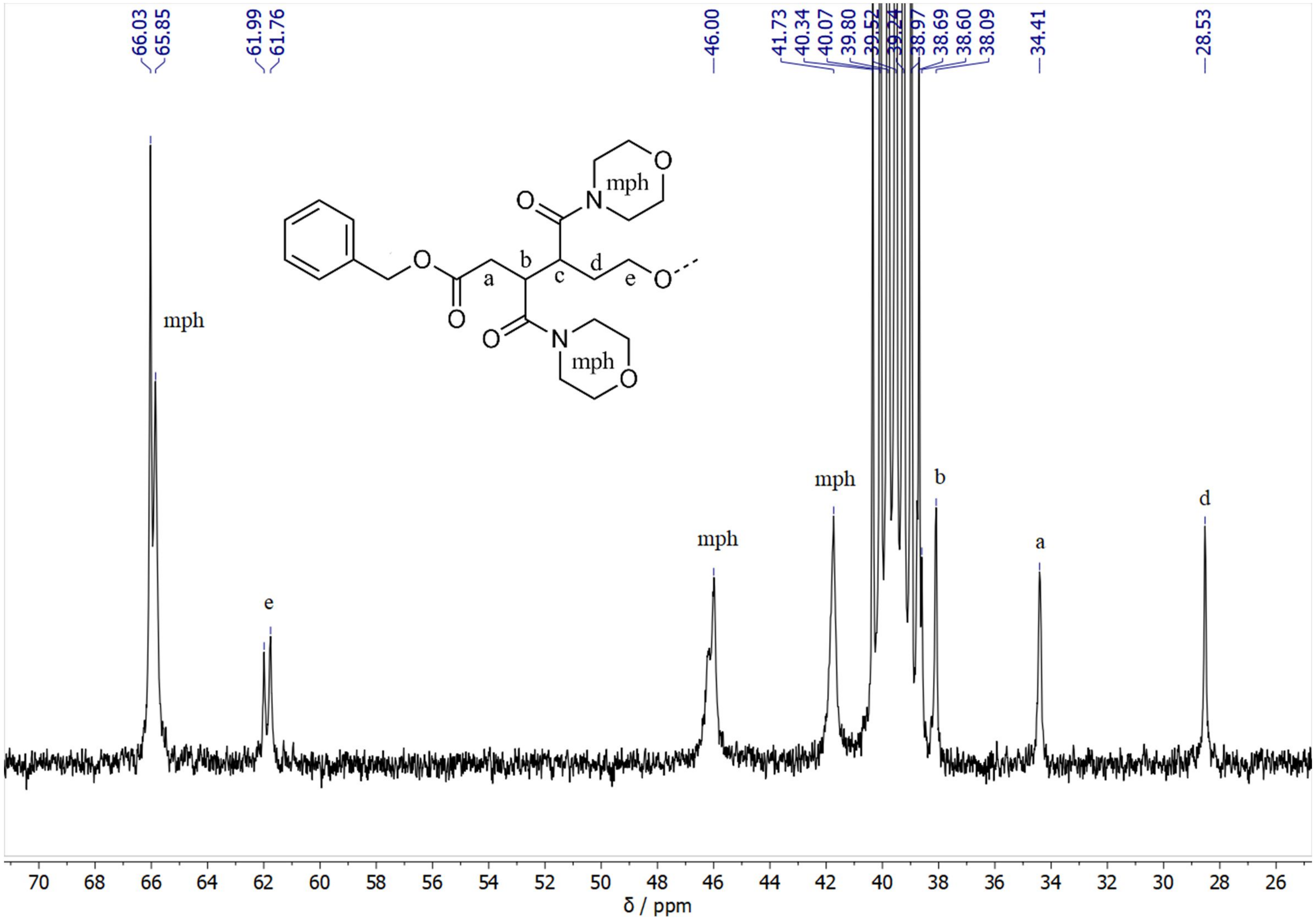

Figure S30. ^{13}C NMR spectrum of MAPCL2 homopolymer in DMSO-d_6 at 50 °C.

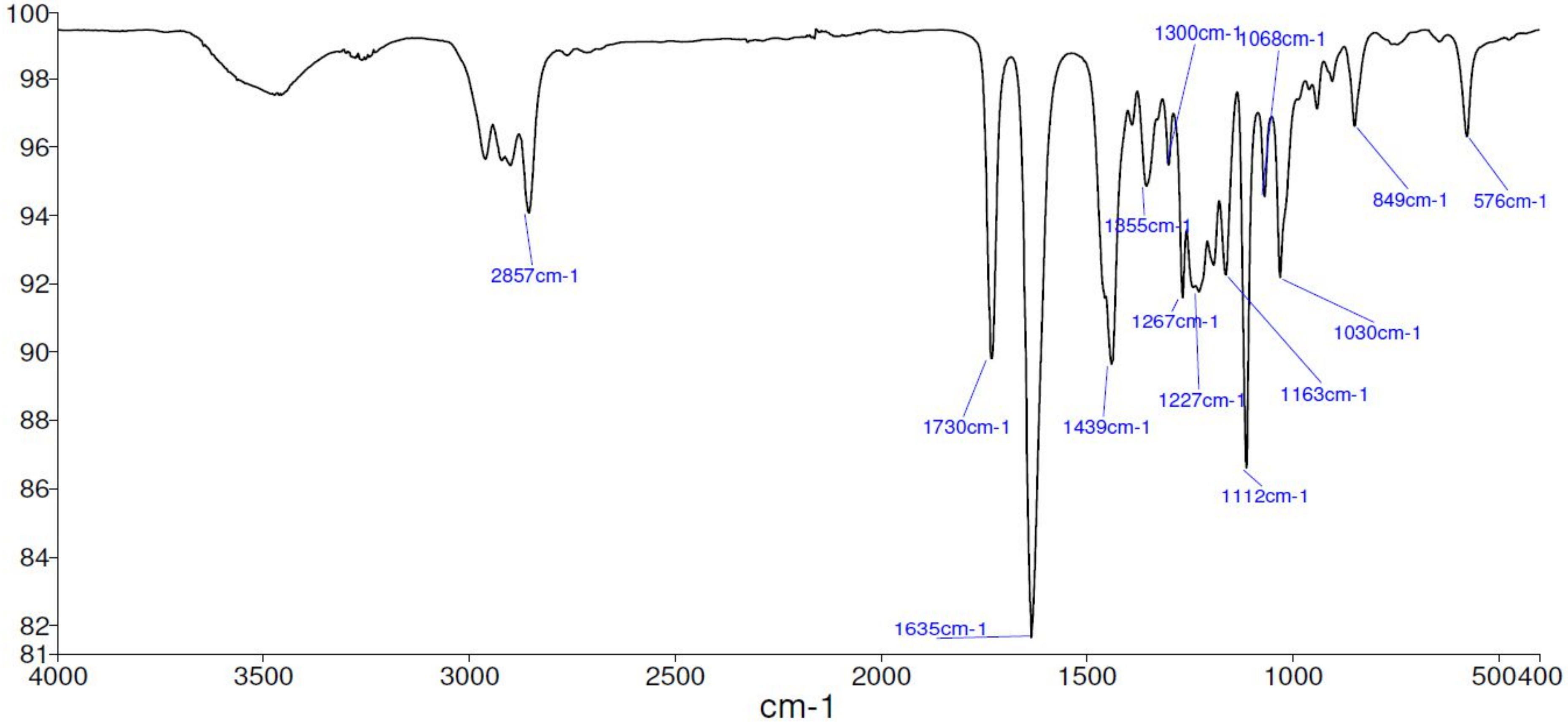

Figure S31. FTIR spectrum of MAPCL2 homopolymer.

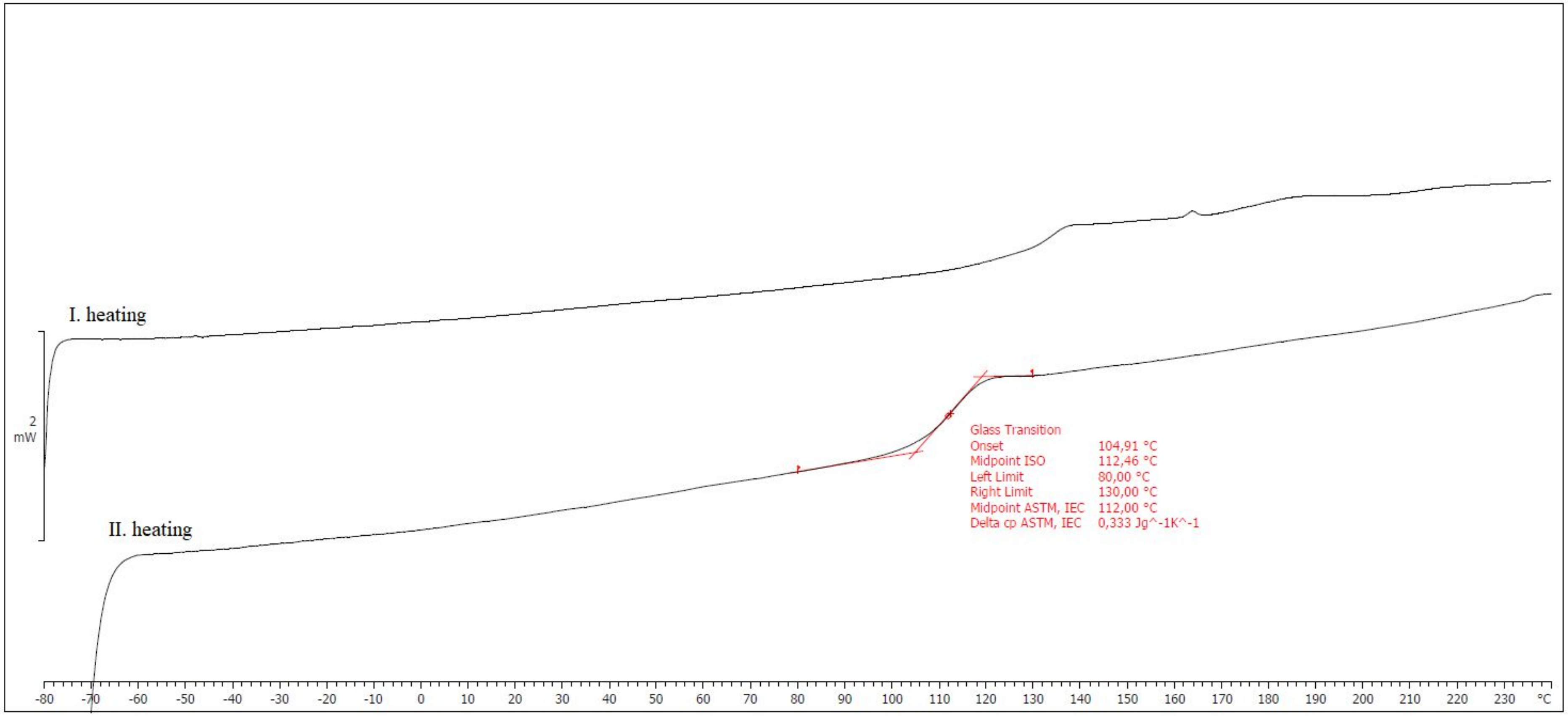

Figure S32. DSC thermogram of MAPCL2 homopolymer.

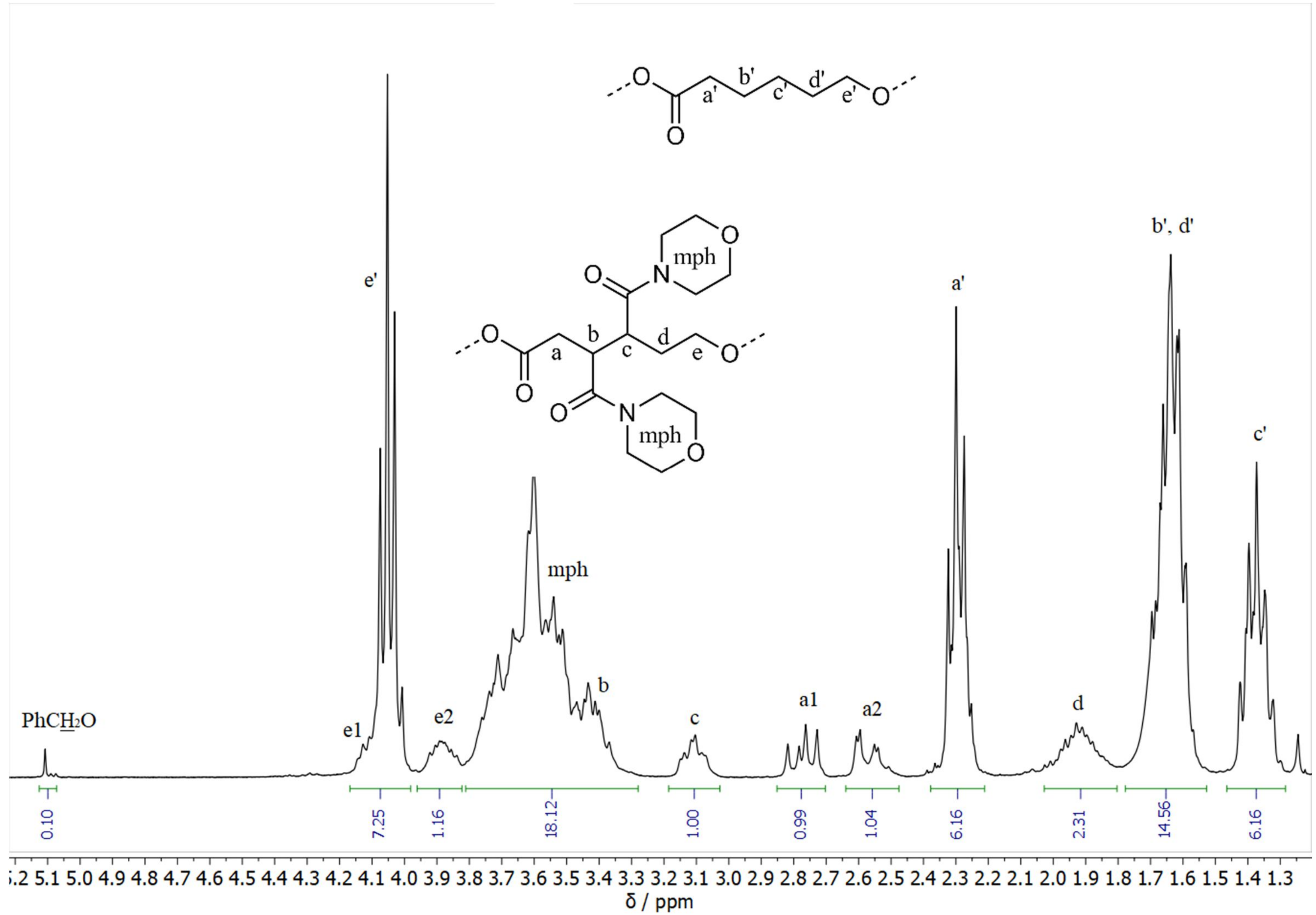

Figure S33. ^{1}H NMR spectrum of PCL-*co*-MAPCL statistical copolymer in $CDCl_3$ at 25 °C.

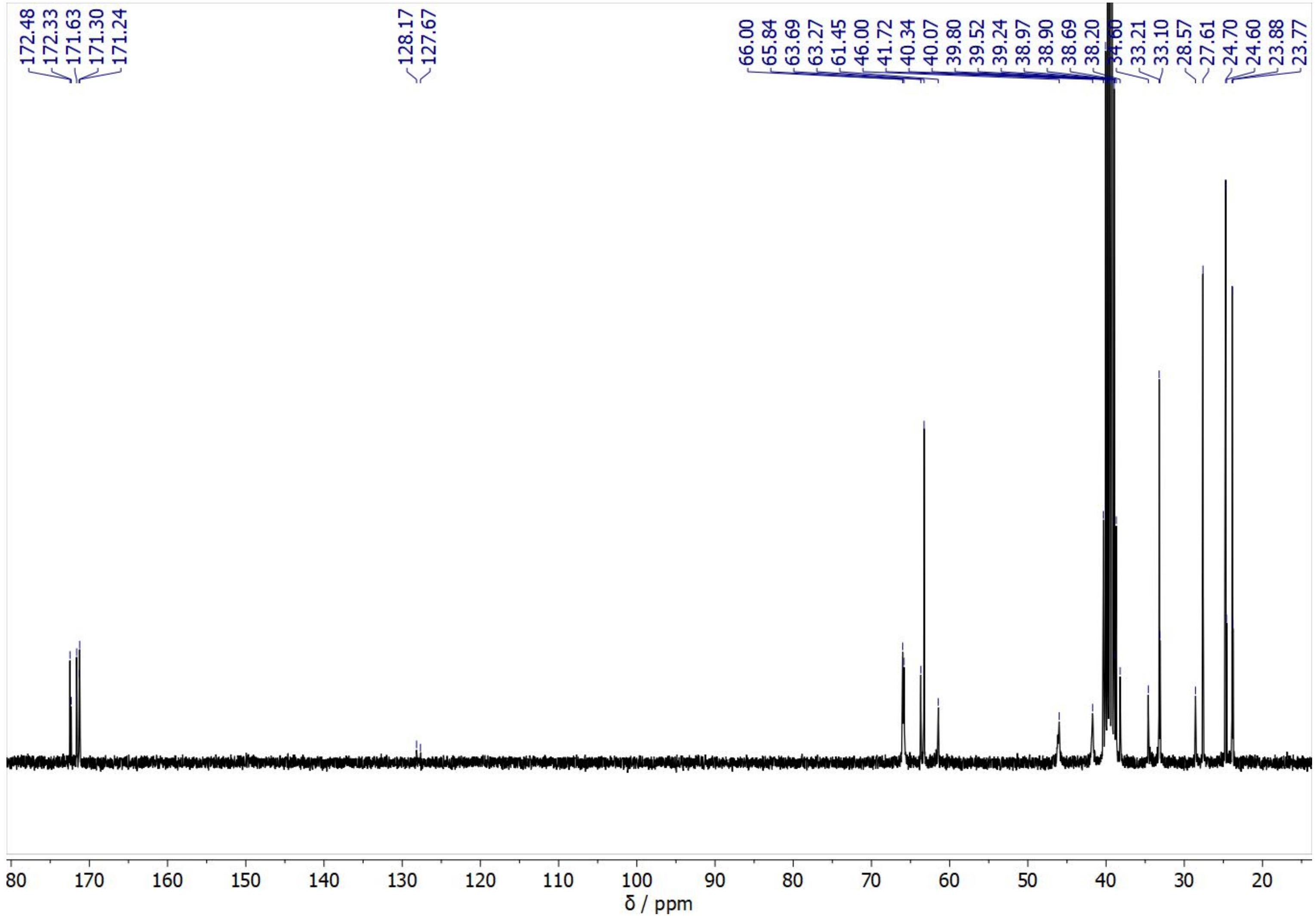

Figure S34. ^{13}C NMR spectrum of PCL-*co*-MAPCL statistical copolymer in DMSO-d_6 at 50 °C.

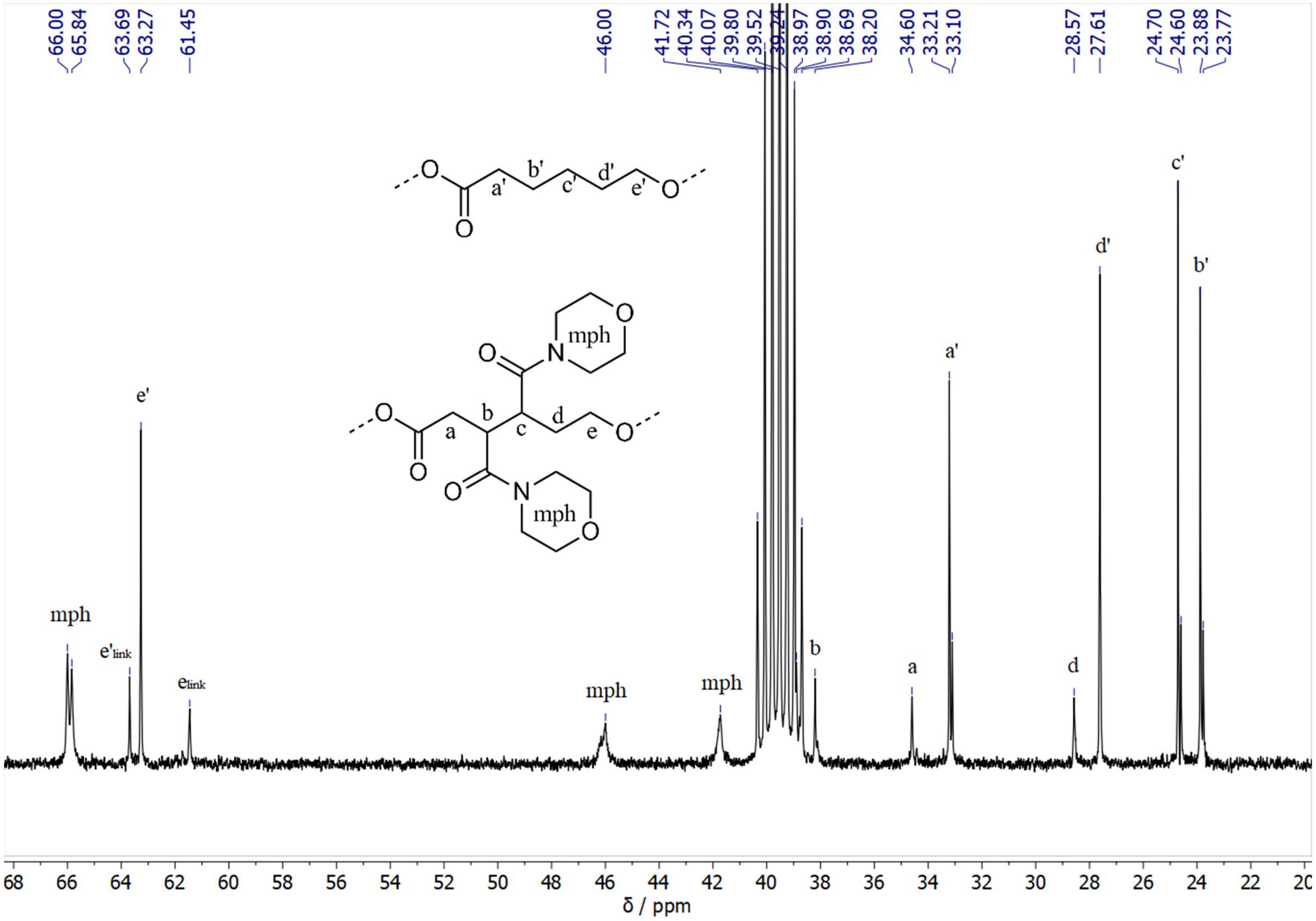

Figure S35. Enlarged ^{13}C NMR spectrum of PCL-*co*-MAPCL statistical copolymer in DMSO-d_6 at 50 °C.

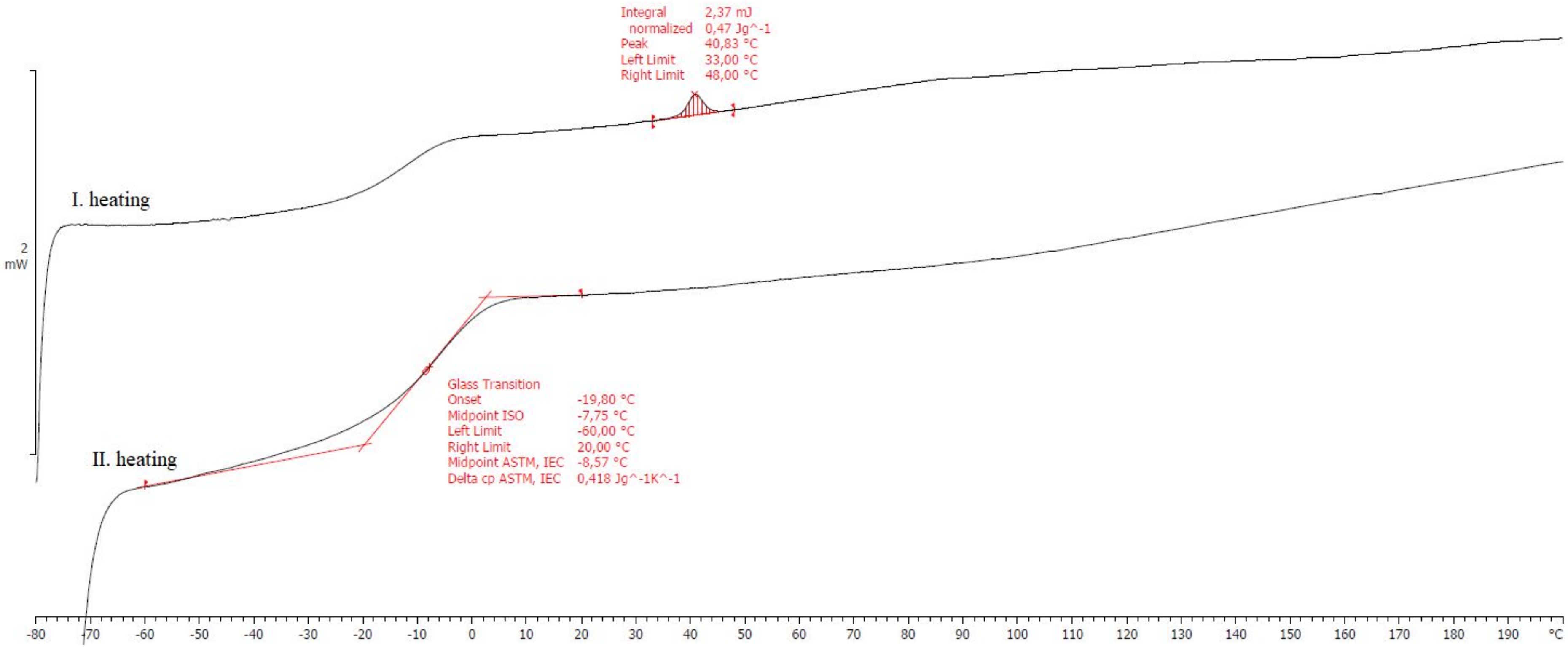

Figure S36. DSC thermogram of PCL-*co*-MAPCL statistical copolymer.

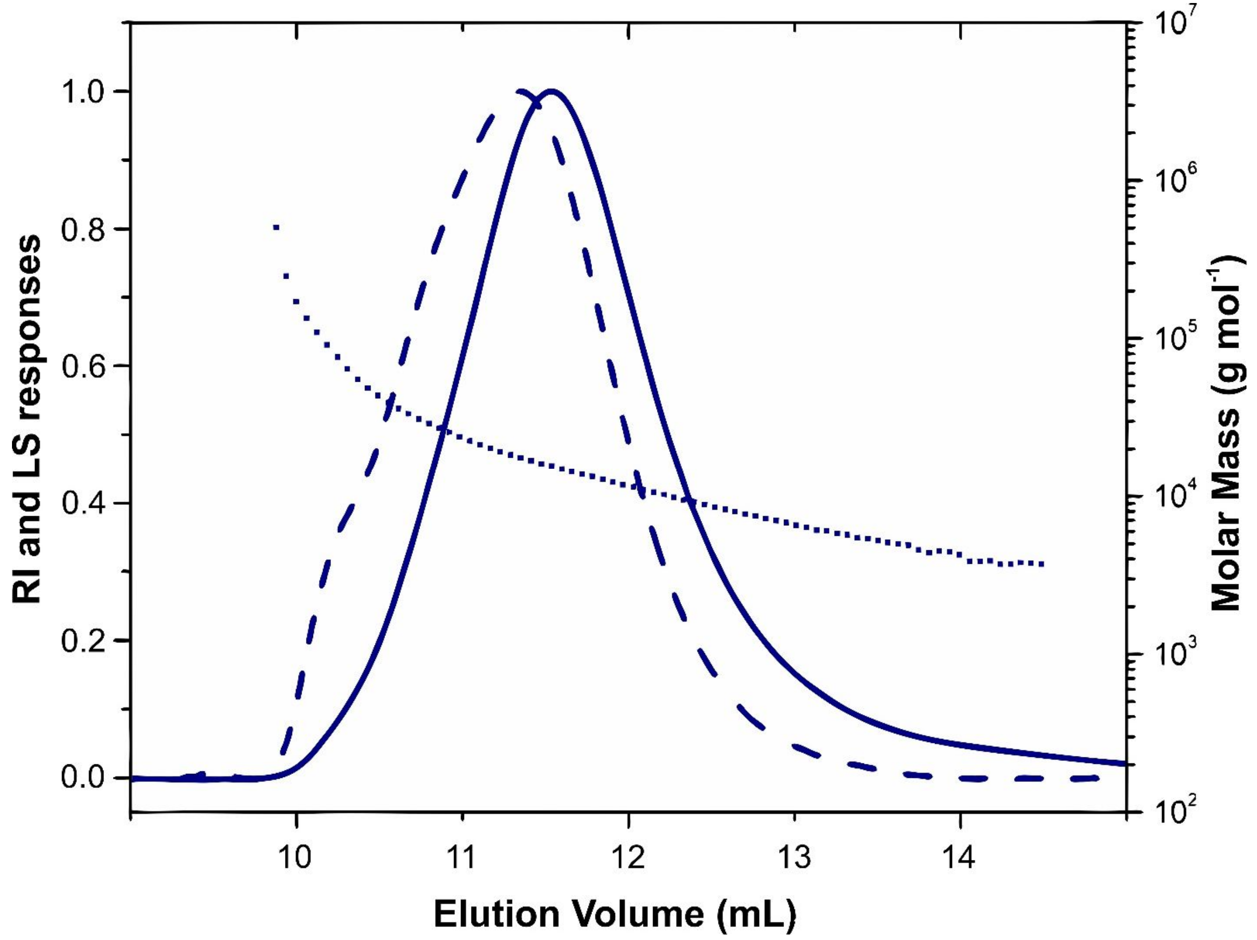

Figure S37. SEC/MALS-RI chromatogram of PCL-*co*-MAPCL copolymer. Solid line: refractive index detector response, dashed line: 90° light-scattering detector response, squares: molar mass vs. elution volume.

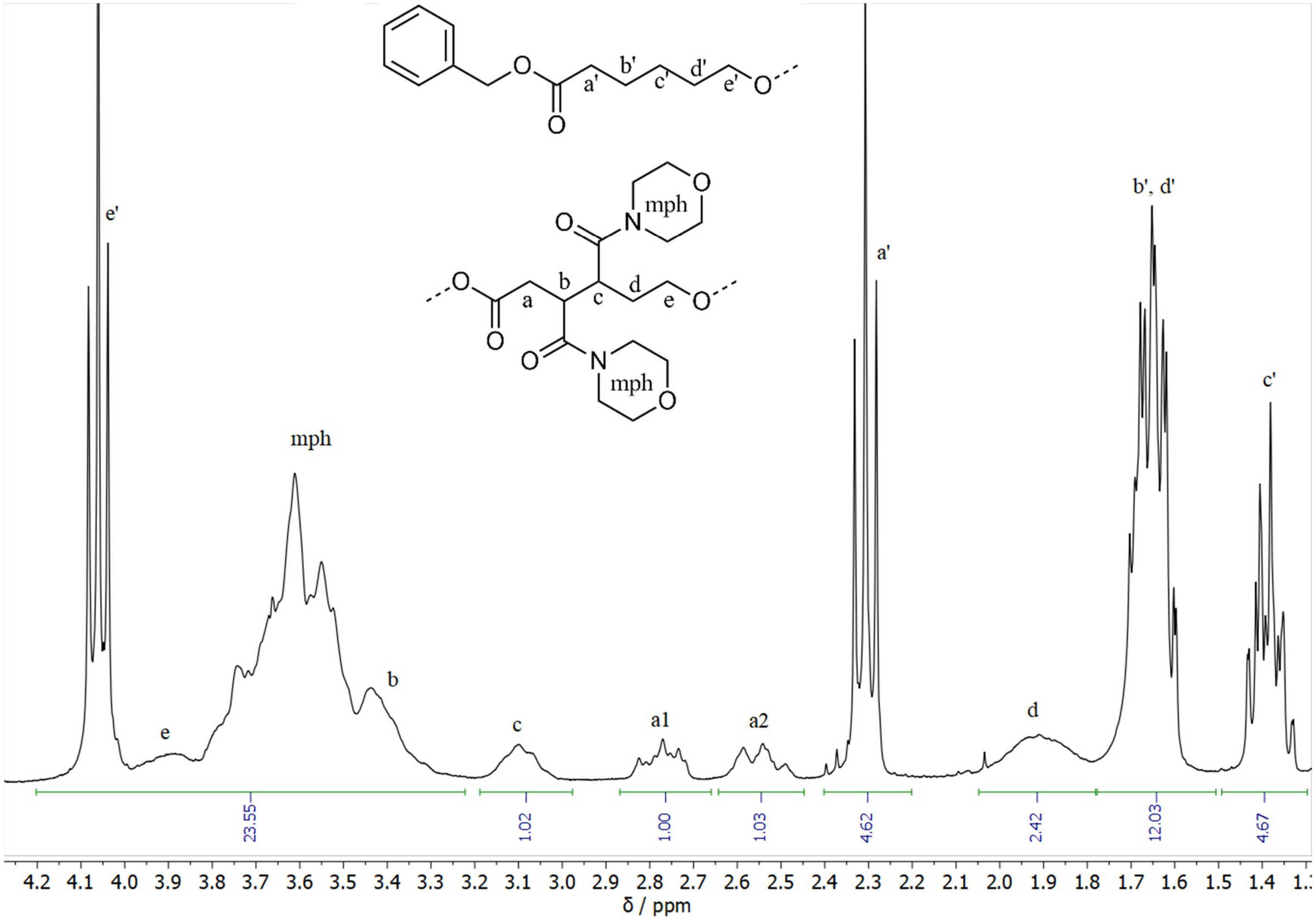

Figure S38. ^{1}H NMR spectrum of PCL-*b*-MAPCL1 block copolymer in $CDCl_3$ at 25 °C.

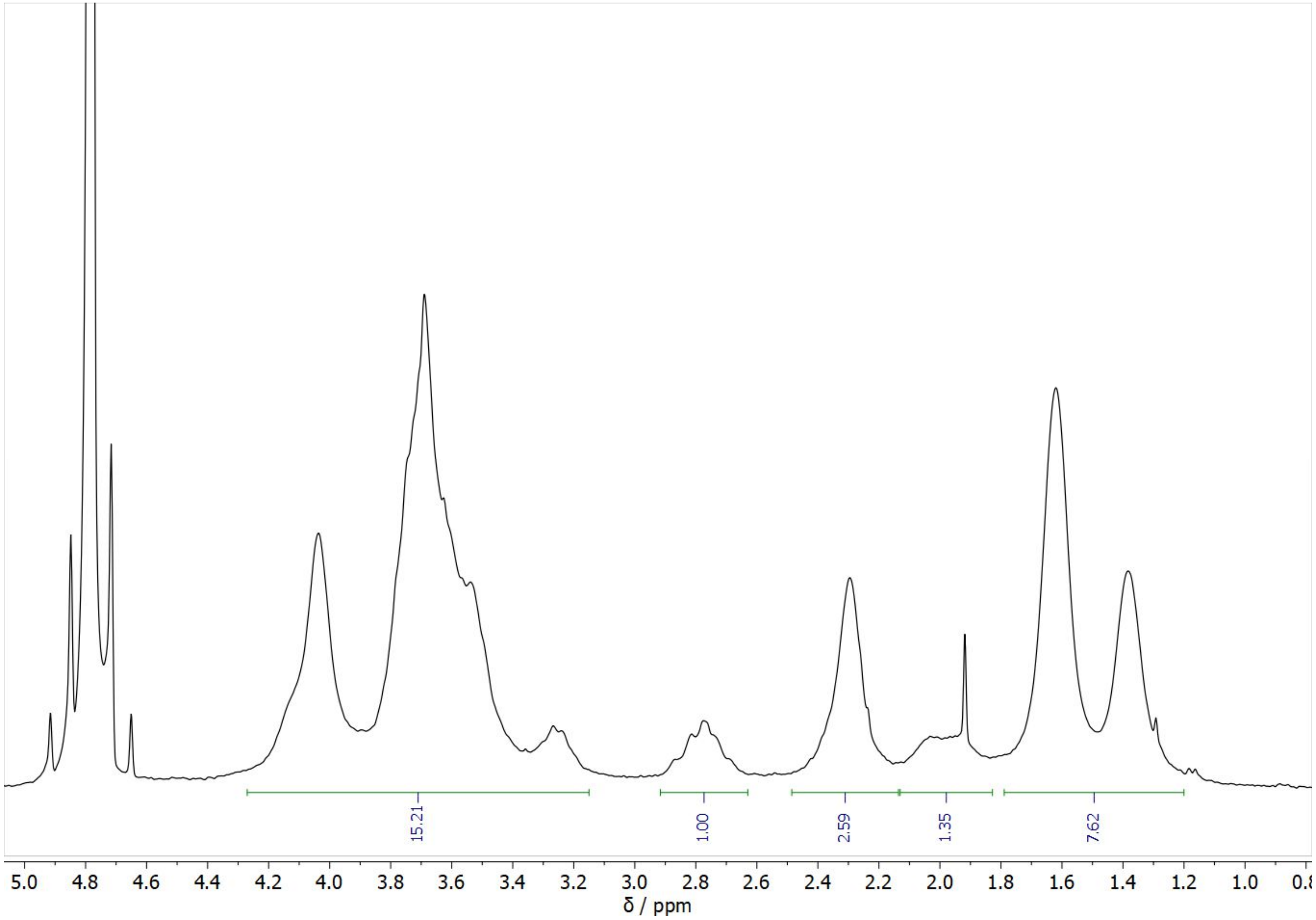

Figure S39. ^{1}H NMR spectrum of PCL-*b*-MAPCL1 block copolymer in D_2O at 25 °C.

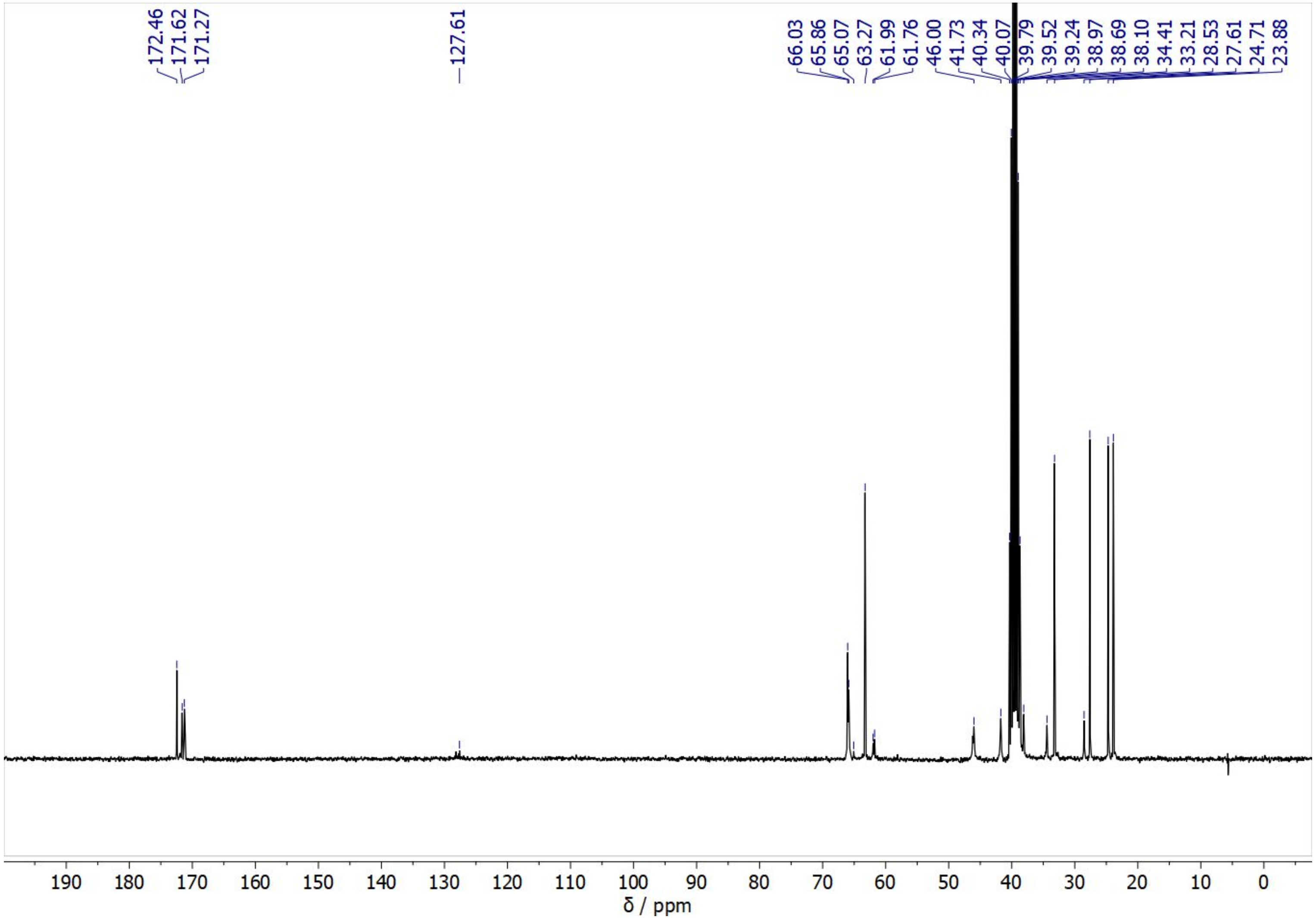

Figure S40. ^{13}C NMR spectrum of PCL-*b*-MAPCL1 block copolymer in DMSO-d_6 at 50 °C.

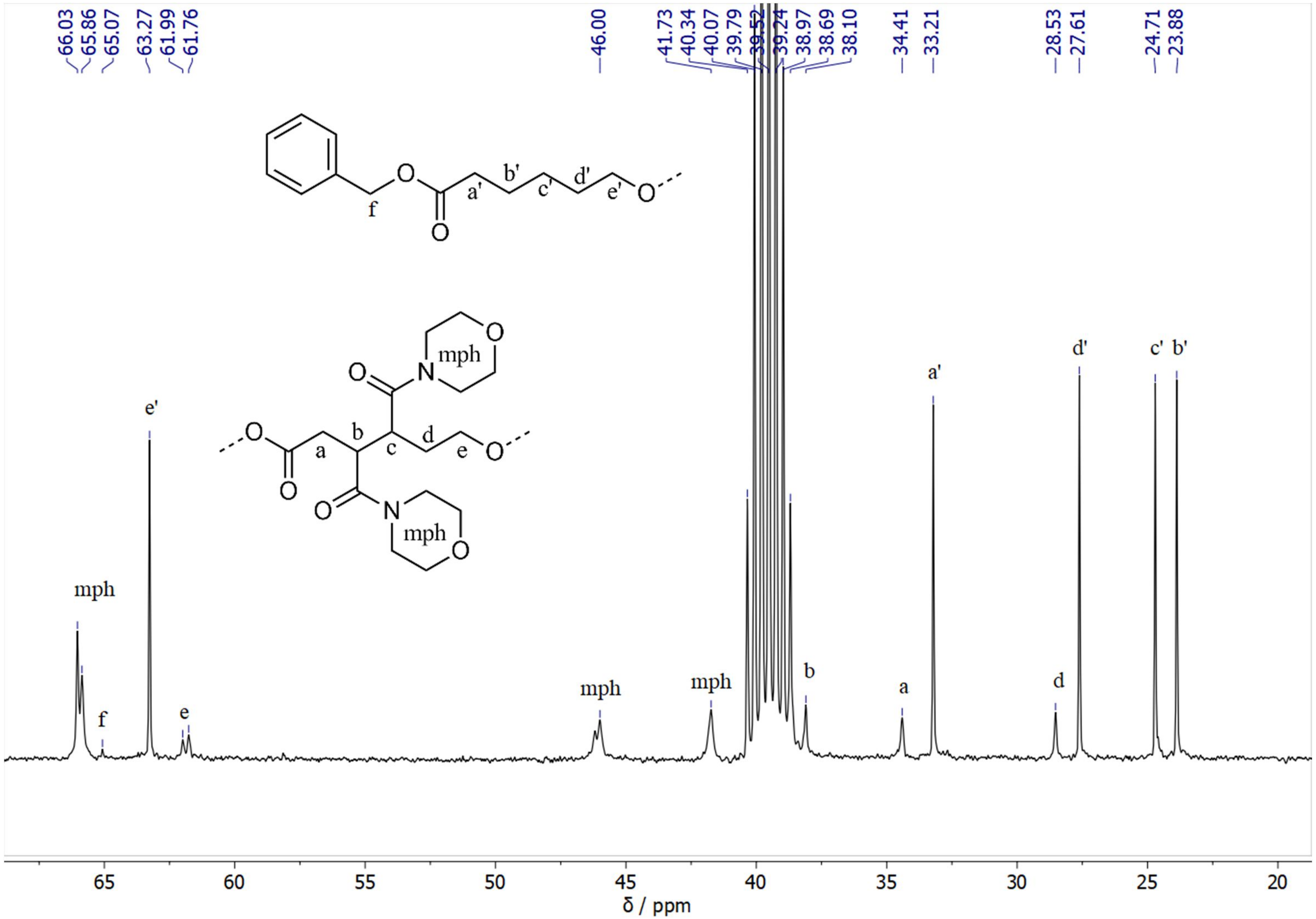

Figure S41. Enlarged ^{13}C NMR spectrum of PCL-*b*-MAPCL1 block copolymer in DMSO-d_6 at 50 °C.

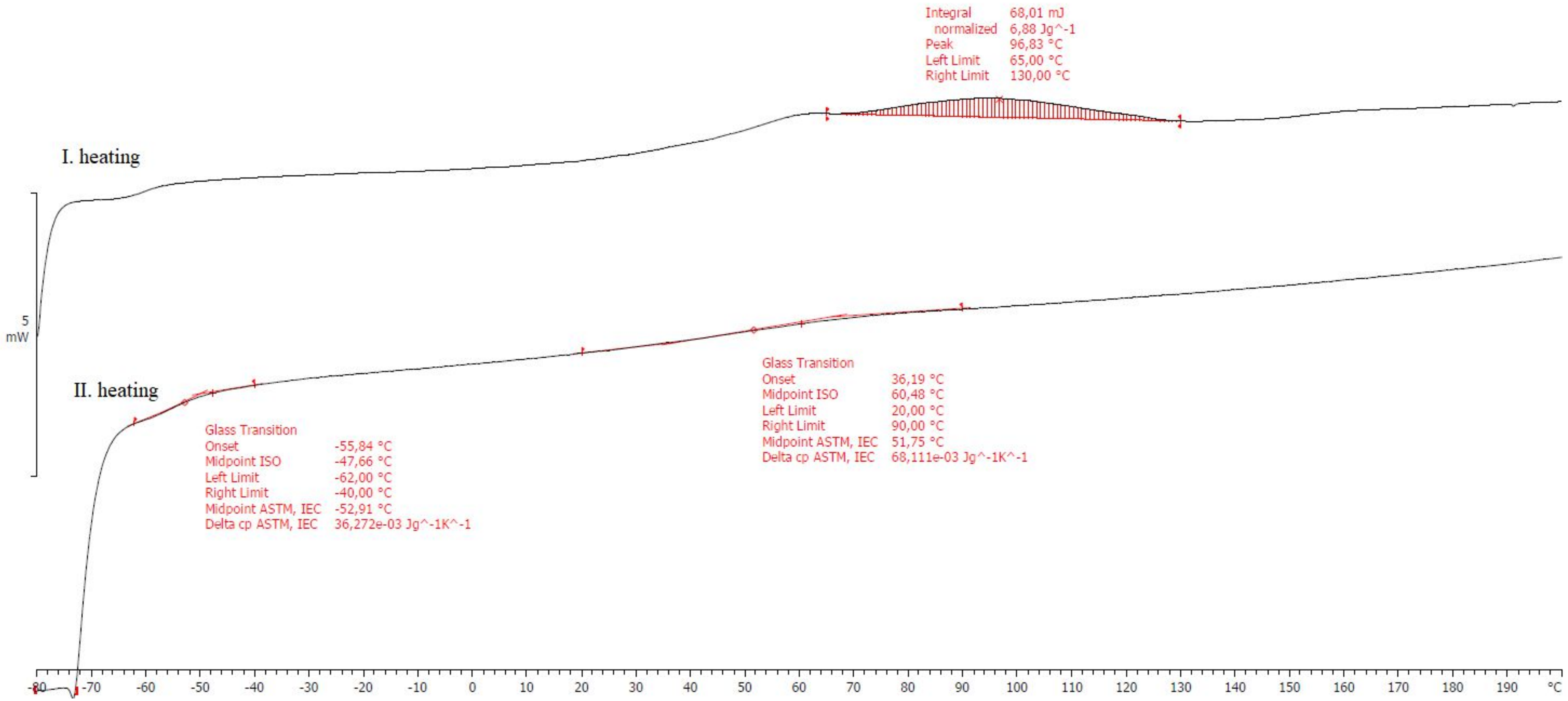

Figure S42. DSC thermogram of PCL-*b*-MAPCL1 block copolymer.

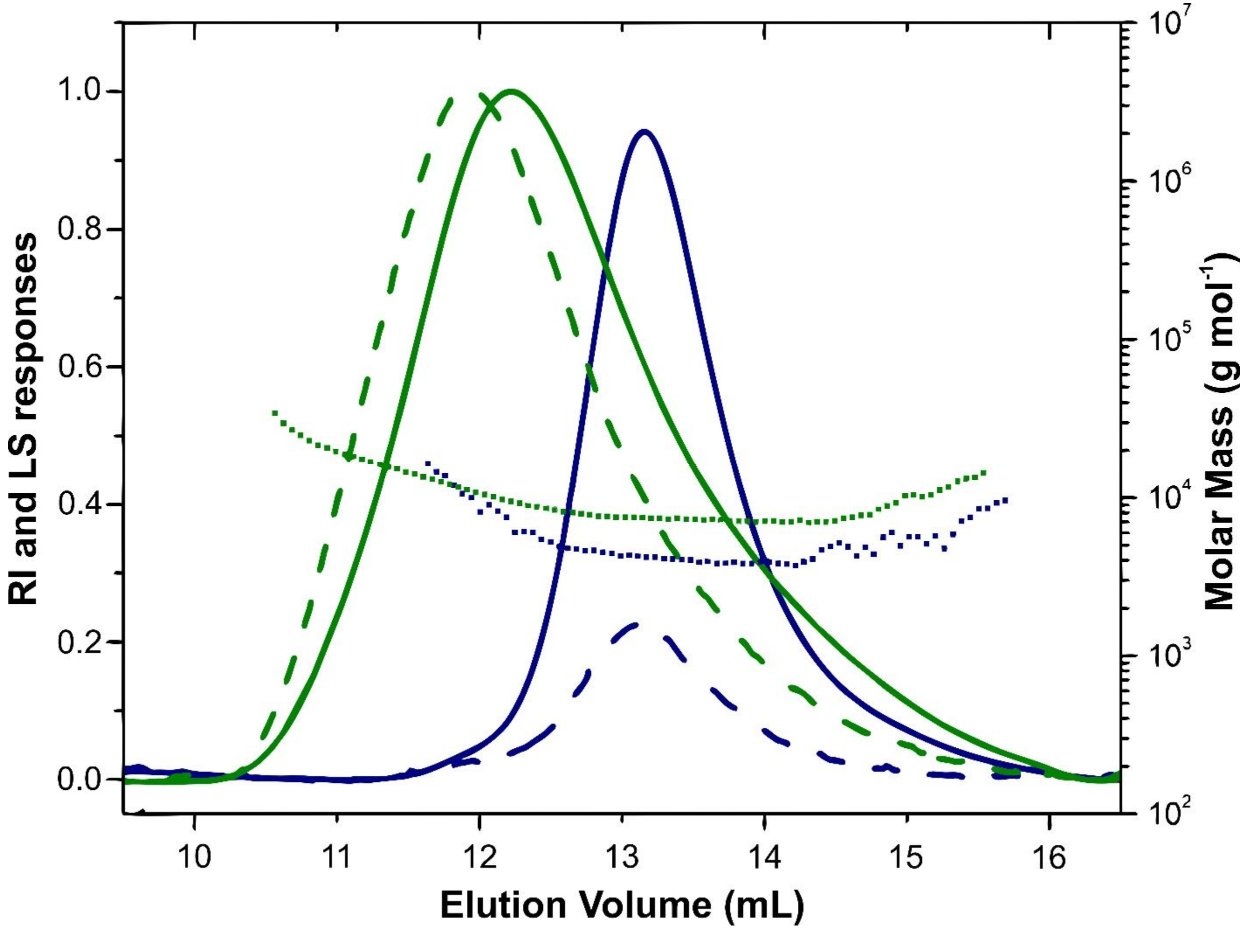

Figure S43. SEC/MALS-RI chromatograms of PCL-*b*-MAPCL1 copolymer (green) and PCL macroinitiator (blue). Solid lines: refractive index detector responses, dashed lines: 90° light-scattering detector responses, squares: molar mass vs. elution volume.

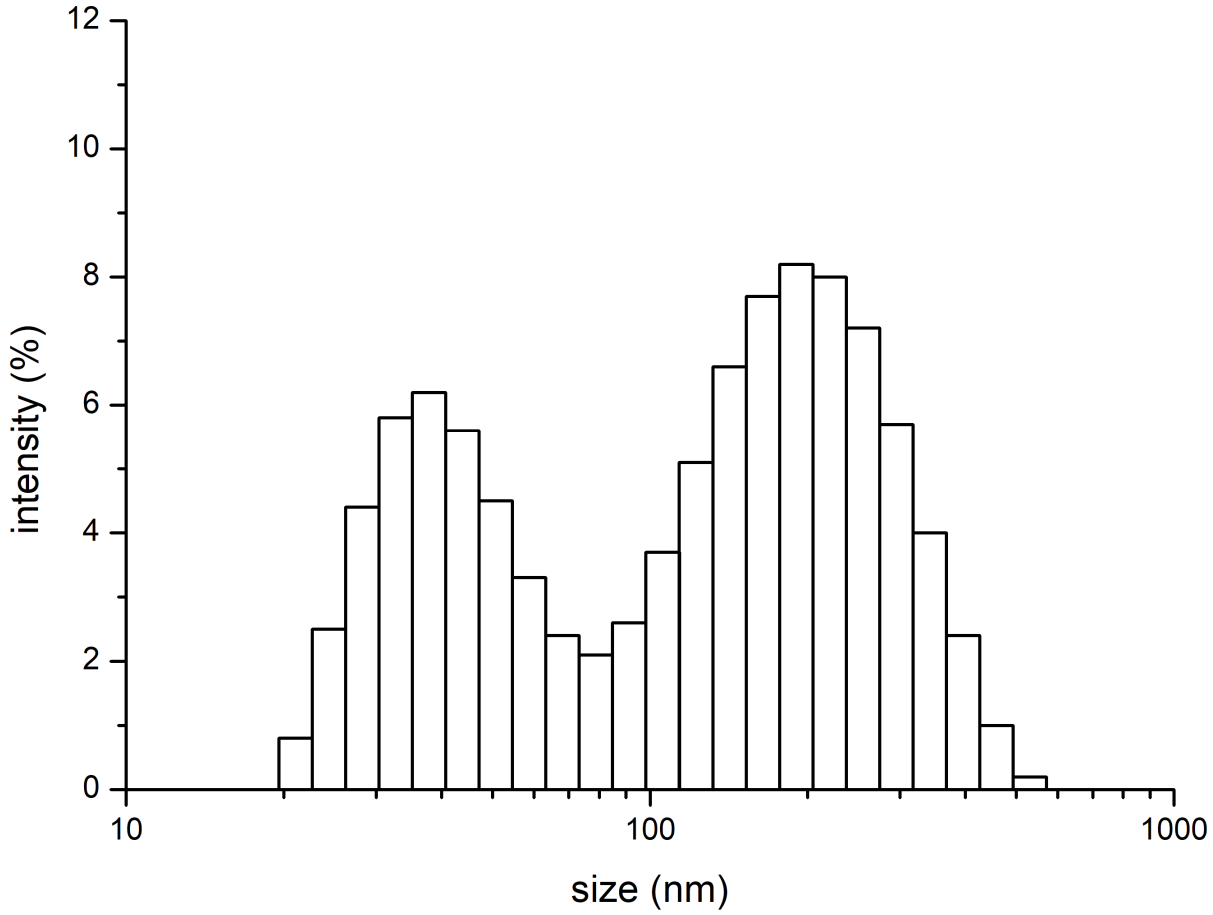

Figure S44. DLS histogram of PCL-*b*-MAPCL1 block copolymer in water at 25 °C.

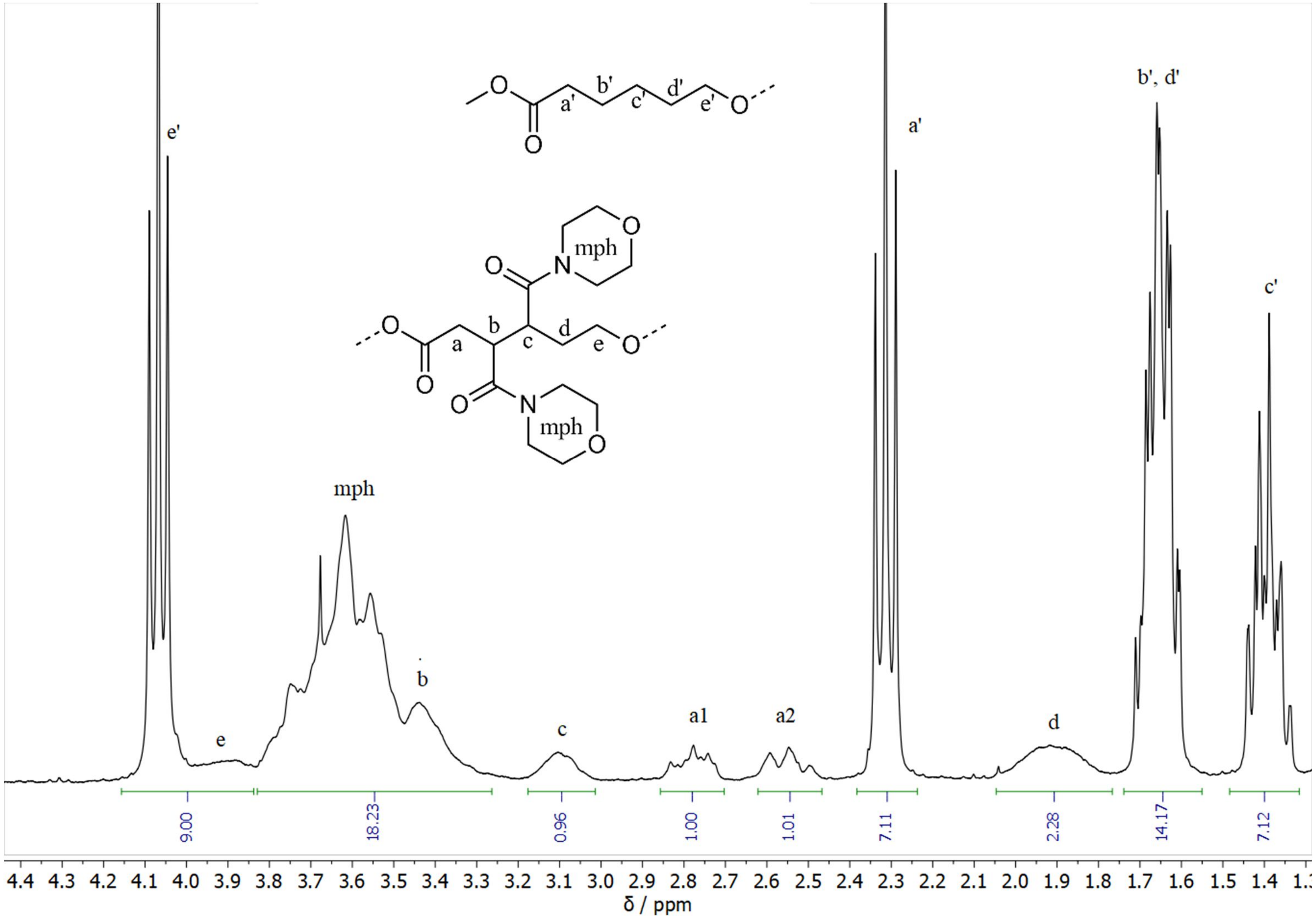

Figure S45. ^{1}H NMR spectrum of PCL-*b*-MAPCL2 block copolymer in $CDCl_3$ at 25 °C.

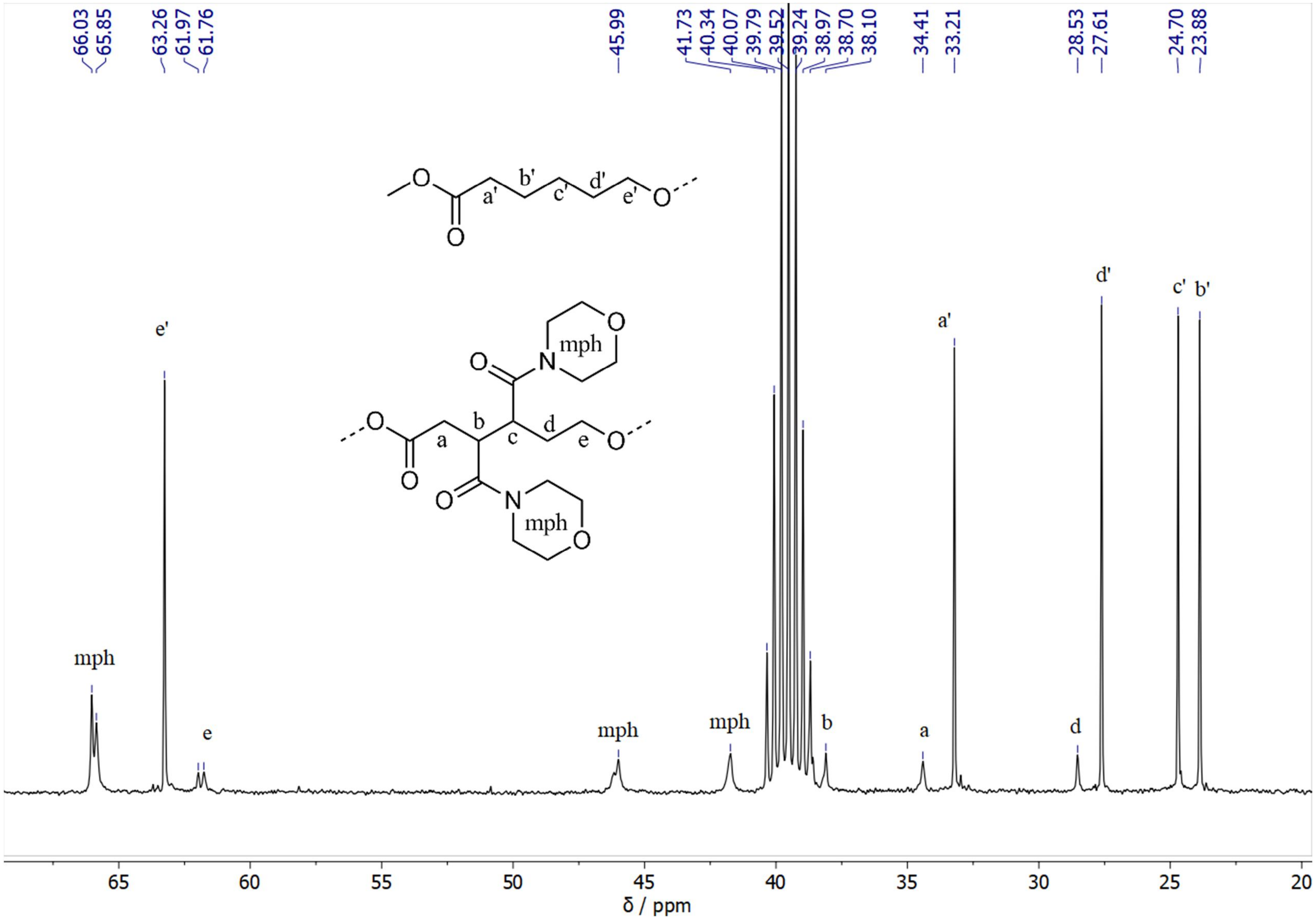

Figure S46. Enlarged ^{13}C NMR spectrum of PCL-*b*-MAPCL2 block copolymer in DMSO-d_6 at 50 °C.

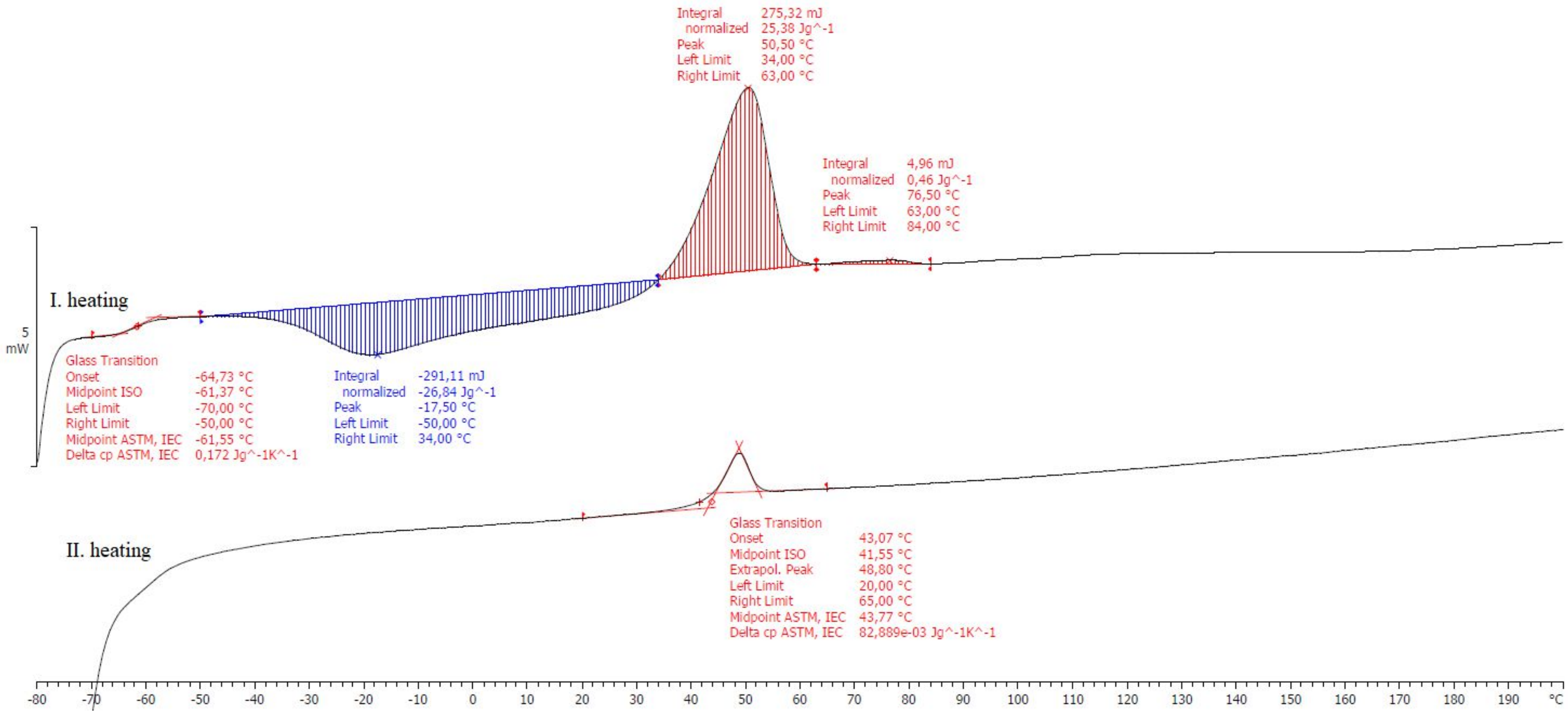

Figure S47. DSC thermogram of PCL-*b*-MAPCL2 block copolymer.

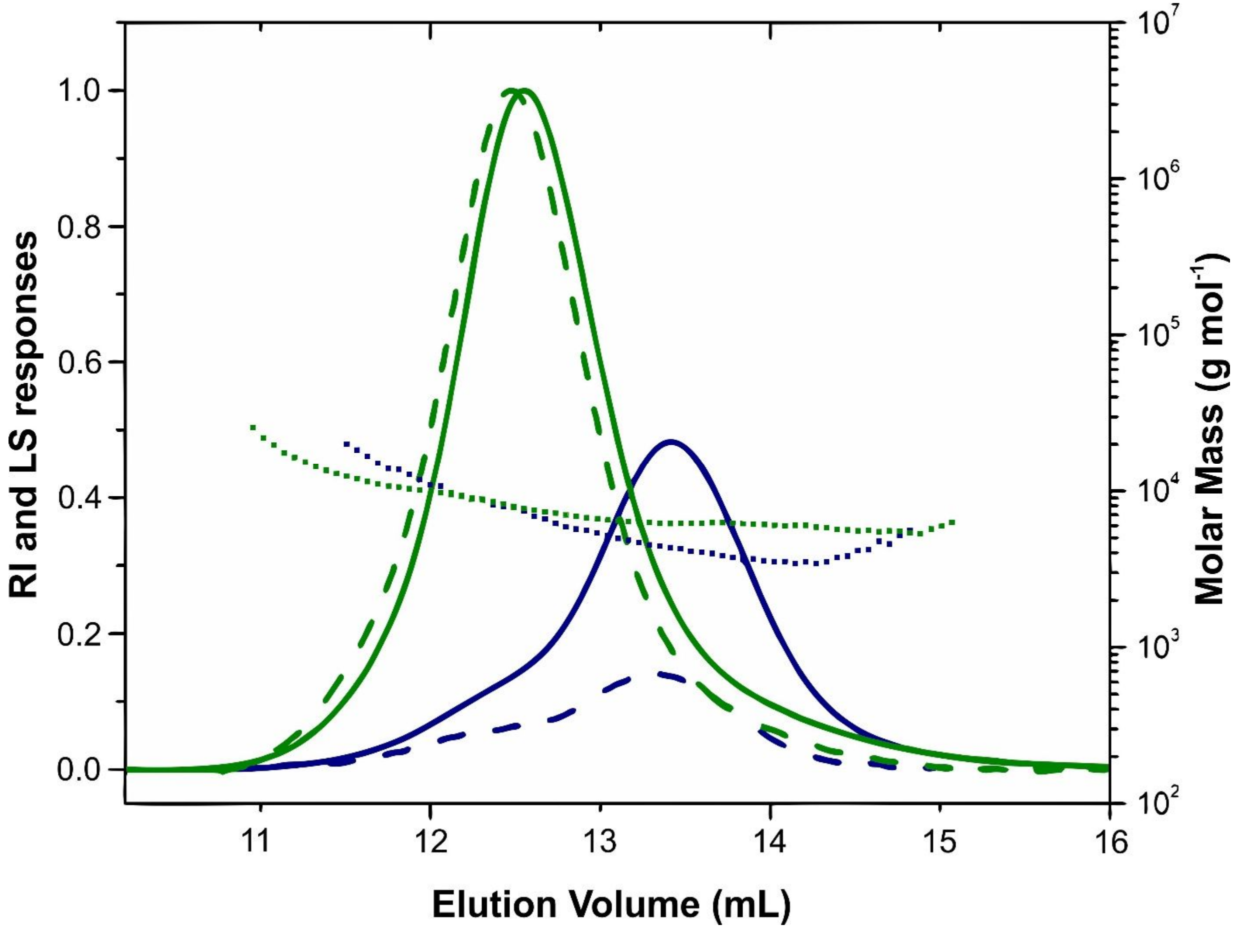

Figure S48. SEC/MALS-RI chromatograms of PCL-*b*-MAPCL2 copolymer (green) and PCL macroinitiator (blue). Solid lines: refractive index detector responses, dashed lines: 90° light-scattering detector responses, squares: molar mass vs. elution volume.

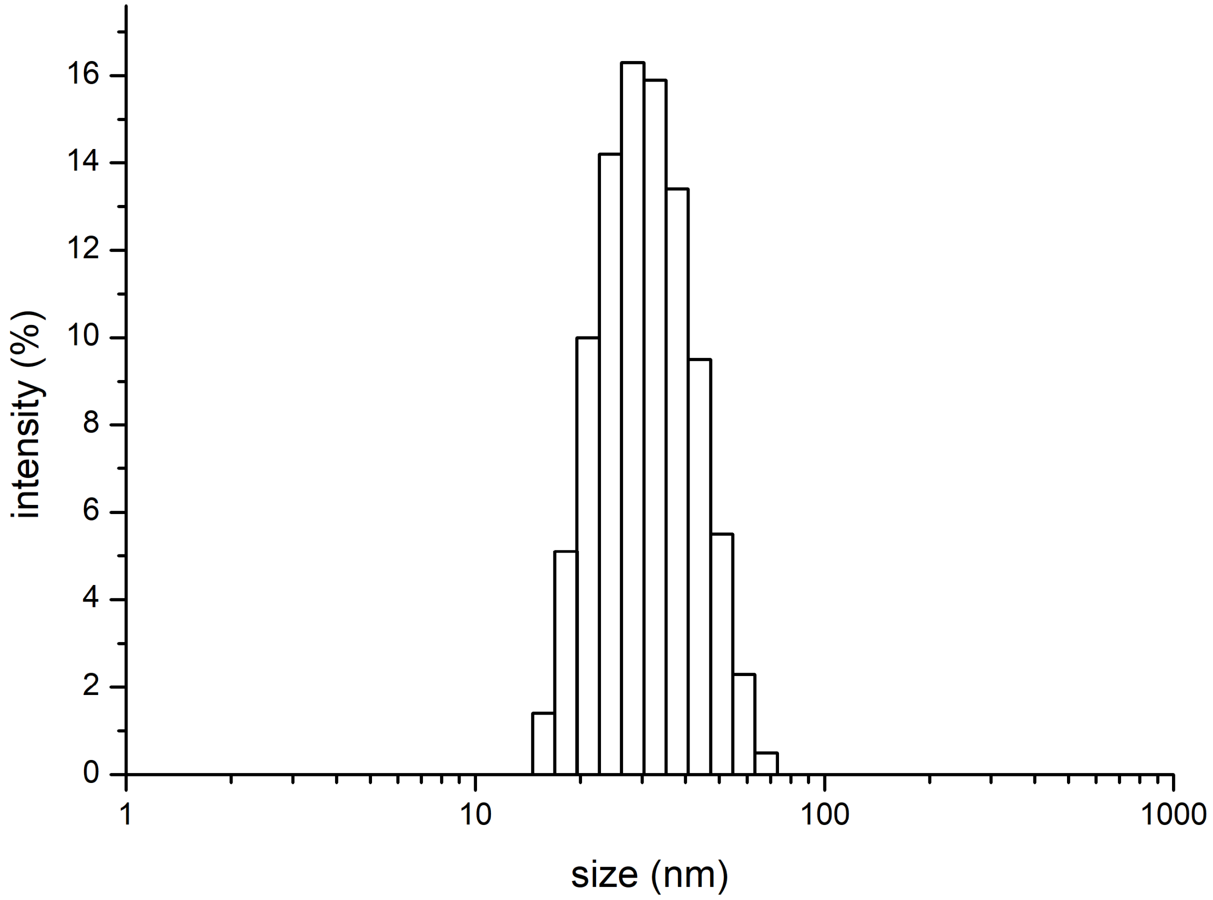

Figure S49. DLS histogram of PCL-*b*-MAPCL2 block copolymer in water at 25 °C.
